# Losartan ameliorates dystrophic epidermolysis bullosa and uncovers new disease mechanisms

**DOI:** 10.15252/emmm.201505061

**Published:** 2015-07-20

**Authors:** Alexander Nyström, Kerstin Thriene, Venugopal Mittapalli, Johannes S Kern, Dimitra Kiritsi, Jörn Dengjel, Leena Bruckner-Tuderman

**Affiliations:** 1 Department of Dermatology, Medical Center - University of FreiburgFreiburg, Germany; 2 ZBSA Center for Biological Systems AnalysisFreiburg, Germany; 3 FRIAS Freiburg Institute for Advanced StudiesFreiburg, Germany; 4 BIOSS Centre for Biological Signalling Studies, University of FreiburgFreiburg, Germany

**Keywords:** collagen VII, dystrophic epidermolysis bullosa, fibrosis, losartan, TGF-β

## Abstract

Genetic loss of collagen VII causes recessive dystrophic epidermolysis bullosa (RDEB)—a severe skin fragility disorder associated with lifelong blistering and disabling progressive soft tissue fibrosis. Causative therapies for this complex disorder face major hurdles, and clinical implementation remains elusive. Here, we report an alternative evidence-based approach to ameliorate fibrosis and relieve symptoms in RDEB. Based on the findings that TGF-β activity is elevated in injured RDEB skin, we targeted TGF-β activity with losartan in a preclinical setting. Long-term treatment of RDEB mice efficiently reduced TGF-β signaling in chronically injured forepaws and halted fibrosis and subsequent fusion of the digits. In addition, proteomics analysis of losartan- vs. vehicle-treated RDEB skin uncovered changes in multiple proteins related to tissue inflammation. In line with this, losartan reduced inflammation and diminished TNF-α and IL-6 expression in injured forepaws. Collectively, the data argue that RDEB fibrosis is a consequence of a cascade encompassing tissue damage, TGF-β-mediated inflammation, and matrix remodeling. Inhibition of TGF-β activity limits these unwanted outcomes and thereby substantially ameliorates long-term symptoms.

## Introduction

Recessive dystrophic epidermolysis bullosa (RDEB) is an inherited skin fragility disorder caused by mutations in the *COL7A1* gene, which encodes collagen VII (C7), an extracellular matrix (ECM) adhesion protein. RDEB skin has greatly reduced mechanical resistance, is injury-prone, and exhibits perturbed wound healing and exaggerated scarring (Nystrom *et al*, 2013). In severe generalized RDEB, perpetual cycles of wounding and scarring lead to fibrotic webbing and, ultimately, fusion of fingers and toes (mitten deformities), joint contractures, generalized soft tissue fibrosis and functional failure of multiple organs (Varki *et al*, 2007). The trauma-exposed, heavily fibrotic sites are prone to develop aggressive squamous cell carcinoma (Ng *et al*, 2012) that constitutes the leading cause of premature death in RDEB (Fine *et al*, 2014).

C7 is expressed at the epidermal–dermal interface, where it forms anchoring fibrils—adhesive structures attaching the epidermal basement membrane to the dermis (Bruckner-Tuderman, 2010; Chung & Uitto, 2010). Functional loss of the fibrils causes epidermal separation, destabilizes the tissue architecture in the papillary dermis and, as a consequence, alters bioavailability of growth factors after tissue damage.

Recessive dystrophic epidermolysis bullosa with its obvious scarring phenotype can be viewed as a fibrotic disorder in which changes in TGF-β activity greatly contribute to disease progression (Leask & Abraham, 2004; Fine *et al*, 2009). TGF-β expression and activity are increased in human RDEB and in mouse models replicating the disorder (Fritsch *et al*, 2008; Ng *et al*, 2012; Kuttner *et al*, 2013; Nystrom *et al*, 2013). Recently, TGF-β was identified as a phenotype modulator in monozygotic twins differentially affected with RDEB (Odorisio *et al*, 2014). Consequently, reducing TGF-β activity may reduce fibrosis, delay development of joint contractures and mitten deformities, and potentially limit lethal squamous cell carcinoma (Fritsch *et al*, 2008; Dietz, 2010; Nystrom *et al*, 2013).

Context- and disease-specific TGF-β-mediated changes have been observed in genetic disorders with increased ECM deposition. Preclinical studies and phase I–III trials to regulate TGF-β with neutralizing isoform-specific antibodies, pan-isoform antibodies, soluble TGF-β receptors as TGF-β traps, blocking the activation of latency-associated peptide-bound TGF-β or decorin (Isaka *et al*, 1996; Akhurst & Hata, 2012) have shown conceptual success for some treatment regimens. However, most of the compounds have not yet found wider clinical use due to issues concerning cost, efficacy, or safety.

Losartan, a small-molecule angiotensin II type 1 receptor antagonist used to treat hypertension, was shown to reduce TGF-β expression and myocardial fibrosis in mice with hypertrophic cardiomyopathy (Lim *et al*, 2001). Subsequently, the efficacy in reducing mechano- and TGF-β-mediated ECM remodeling of aortic roots and heart walls in Marfan syndrome was established (Habashi *et al*, 2006; Ramirez & Rifkin, 2012; Pees *et al*, 2013; Cook *et al*, 2014). The primary effect of losartan on tissue remodeling in Marfan syndrome is likely to be mechanosensitive, mediated through reduction in blood pressure, since treatment with β1 receptor antagonists slows aortic dilatation to a similar degree as losartan (Lacro *et al*, 2014). This is in line with that fibrillin-1, the protein at fault in Marfan syndrome, is part of a tissue-mechanosensing complex (Cook *et al*, 2014). However, in fibrotic conditions more directly dependent on TGF-β activity, for example, in hepatic, renal or interstitial pulmonary fibrosis, clinical trials with losartan have been successful in slowing fibrosis and reducing of circulating TGF-β (Campistol *et al*, 1999; Terui *et al*, 2002; Colmenero *et al*, 2009). Importantly, the effectiveness of targeting TGF-β activity for treatment of fibrosis is largely governed by the organ-specific composition of the ECM and the type of injury. Consequently, it is pivotal to assess the therapeutic applicability of losartan for each individual constellation.

Evidence-based therapies are urgently needed for RDEB. Clinical pilot trials of prospective therapies have shown some promise (Wagner *et al*, 2010; Petrof *et al*, 2013; Venugopal *et al*, 2013), but efforts to achieve safe and effective causal treatments still face substantial hurdles. An alternative approach is to ameliorate the RDEB phenotype by targeting major disease-contributing factors downstream the initial C7 loss, for example, by limiting ECM remodeling and response to tissue damage by modulating TGF-β activity. Although not a cure, such treatments benefit patients by increasing functionality and improving quality of life. Indeed, the development of low risk symptom relief therapies has currently highest priority for patients (Davila-Seijo *et al*, 2014).

Here, we evaluated TGF-β inhibition through losartan in RDEB in a preclinical setting. Losartan effectively reduced TGF-β levels in RDEB cells *in vitro*, and in the skin and the circulation of RDEB mice. Lower Tgf-β activity led to significantly slower progression of fibrotic digit fusion/mitten deformities without major adverse effects. Whole-skin proteomics revealed that losartan effectively normalized the abundance of ECM proteins and the pro-inflammatory milieu in RDEB skin. Collectively, the data indicate that losartan significantly ameliorates RDEB-specific signs and improves the phenotype. Thus, it has the potential as a first-line disease-modulating therapy for RDEB that alleviates symptoms and, ultimately, delays or prevents progression of squamous cell carcinoma.

## Results

### Losartan reduces RDEB fibrosis

As a rationale for the evaluation of losartan treatment, we first showed that patients with RDEB display increased TGF-β levels and activity in wounds, and in the circulation (Supplementary Fig S1) (Ng *et al*, 2012; Kuttner *et al*, 2013; Nystrom *et al*, 2013; Wang *et al*, 2013b; Odorisio *et al*, 2014). *In vitro*, losartan effectively limited the fibrotic potential of RDEB fibroblasts, as measured by cell contractility. The mechanism of action included reduced expression of TGF-β, of thrombospondin-1 (TSP1), an activator of latent TGF-β, and of collagen I, a TGF-β target in the ECM (Supplementary Fig S2).

**Figure 1:**
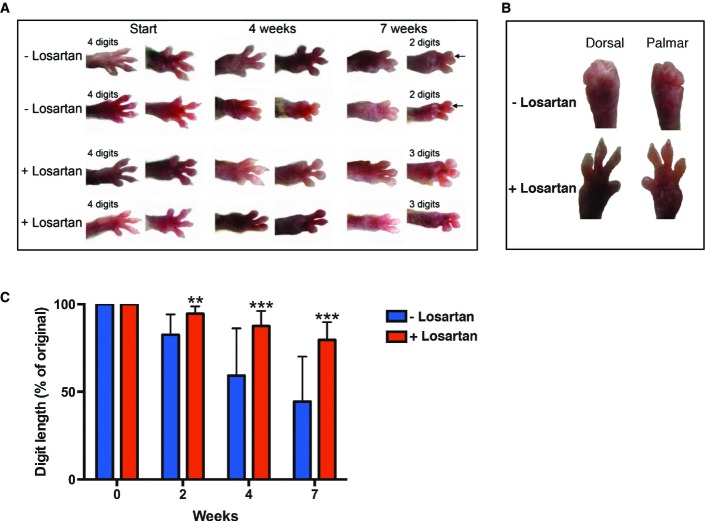
Losartan treatment delays RDEB fibrosis progression *in vivo* Dorsal and palmar view of the right forepaw of the C7-hypomorphic mice. At the start of treatment, the mice were on average 5.5 weeks old. The treated group received 0.6 g losartan per liter drinking water. Shown are photographs of two untreated mice and two mice receiving losartan at the start, after 4 weeks, and at the end of the experiment after 7 weeks. Note the fibrosis-driven loss and fusion of digits with time; the arrows indicate digit fusion.

For the treatment of RDEB mice with losartan, progression of digit fusion in the forepaws was chosen as a primary clinical readout of fibrosis; the final end points were joint contractures and fusion of digits (Fig1A). Losartan treatment was started at the time of the first visible toe length reduction in order to perform the studies in a more homogenous group, since the large majority, but not all, of C7-hypomorphic mice develop mitten deformities (Fritsch *et al*, 2008). On average, the mice were 5.5 weeks old at the beginning of the experiment (control group 39 ± 7 days, treatment group 38 ± 8 days). Losartan was administered in drinking water at a concentration of 0.6 g/l (Habashi *et al*, 2006), estimated average daily dose 200 mg/kg body weight, and the control group was given regular drinking water. The treated group displayed no discomfort or visible signs of adverse events of the drug. Due to extensive mutilating deformities and declining health in the control group, we could not follow control mice for much longer than 7 weeks. Therefore, the mice were followed for 7 weeks—a time point where substantial loss and/or fusion of digits had occurred in the control group (Fig1A). At this time point, the losartan-treated mice in general exhibited significantly lesser fusion of digits (Fig1A). C7-hypomorphic mice displayed variation in the rate of digit webbing/fusion and responsiveness to losartan; Fig1B shows forepaws of a good responder at 7 weeks of losartan treatment vs. a control mouse that showed rapid progression of forepaw deformities. To obtain an unbiased value of the protective effect of losartan, we measured the reduction of the two most pronounced toes on the murine forepaw over time. The data generated from these analyses showed that losartan reduced the rate of digit shortening. Losartan significantly protected against digit loss already after 2 weeks of treatment and continued to do so throughout the observation period (2 weeks, untreated 82.6 ± 11.6% vs. treated 94.5 ± 4.2%; 4 weeks, untreated 59.3 ± 26.9% vs. treated 87.6 ± 8.6%; 7 weeks, untreated 44.5 ± 25.6% vs. treated 79.6 ± 10.0%; for 2 weeks, **P =* *0.0021, and for 4 and 7 weeks, ***P < 0.001). Further, linear regression analysis of the data showed that untreated mice lost 8.5 ± 0.5% digit length per week, and the prediction was that complete digit loss would occur within 11.8 ± 0.7 weeks. In contrast, losartan-treated mice lost only 3.0 ± 0.2% digit length per week, and this would result in complete loss of digits within 33.8 ± 2.3 weeks (*R*^2^ untreated = 0.97, and treated = 0.99).

Forepaws viewed dorsally or palmary after 7 weeks of treatment ± losartan. Shown is a good responder of losartan treatment and one mouse from the control group with rapid mutilation rate.

Bar graph of forepaw digit length in age-matched untreated (blue) vs. losartan-treated C7-hypomorphic mice (red) after 2, 4, and 7 weeks of treatment. The length of digits at the start of the experiment was set to 100%; the quantification procedure is described in detail in the Materials and Methods section. Losartan very potently inhibited the reduction of digit length. Values represent mean ± S.D. Due to the inherent heterogeneity in disease progression, equal variance could not be expected and statistical significance was therefore analyzed by the unpaired *t*-test with Welch’s correction; for 2 weeks, **P =* *0.0021, and for 4 and 7 weeks, ***P < 0.001 (*n* = 14 per group).

**Figure 2:**
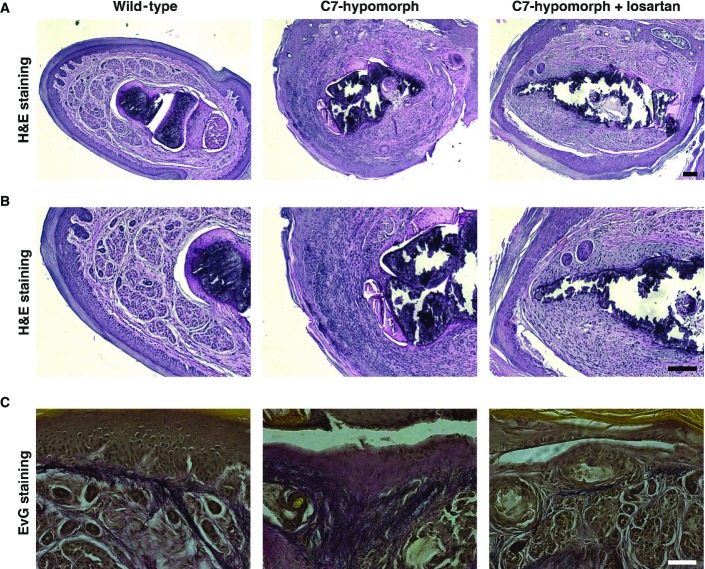
Losartan treatment ameliorates histological signs of RDEB fibrosis Cross sections of paraffin-embedded forepaws of C7-hypomorphic mice treated with losartan for 7 weeks, age-matched untreated C7-hypomorphic mice, and wild-type mice were stained with H&E (A, B) and Elastica van Gieson (EvG) (C).
A, B H&E staining in low (A) and higher (B) magnification of the same forepaw digits. Note widening of the dermis, rich infiltration of inflammatory cells, and deposition of dense material in untreated C7-hypomorphic forepaw digits compared to wild-type. Losartan effectively reduced dermal width, inflammatory infiltrates, and deposition of dense fibrotic material. However, losartan treatment did not protect against friction-induced dermal–epidermal separation visible as epidermal detachment in untreated and losartan-treated C7-hypomorphic digits. Scale bars* *=* *100 μm.

Careful histological examination showed that losartan did not protect C7-deficient paws from blistering but limited subsequent excessive scarring. Untreated paws displayed excessive inflammation, deposition of dense collagenous fibrotic material, disorganization of elastic fibers, and thickening of the dermis, as compared to wild-type paws (Fig2). Although dermal–epidermal separation was still clearly detected in paws of C7-hypomorphic mice treated with losartan for 7 weeks, they exhibited markedly less inflammatory infiltrates, fibrosis, reduced collagen deposition, better arranged elastic fibers, and a tendency to thinner dermis, as compared to untreated C7-hypomorphic paws (Fig2).

C EvG staining of forepaw digits as in (A, B). In wild-type digits, the dermis elastic fibers (black) are densely organized in the papillary dermis. Untreated C7-hypomorphic digits show increased collagen deposition (red) and loosening and disarrangement of elastic fiber organization (black), losartan treatment reduces collagen deposition and improves the appearance of elastic fibers. Scale bar* *=* *50 μm.

**Figure 3:**
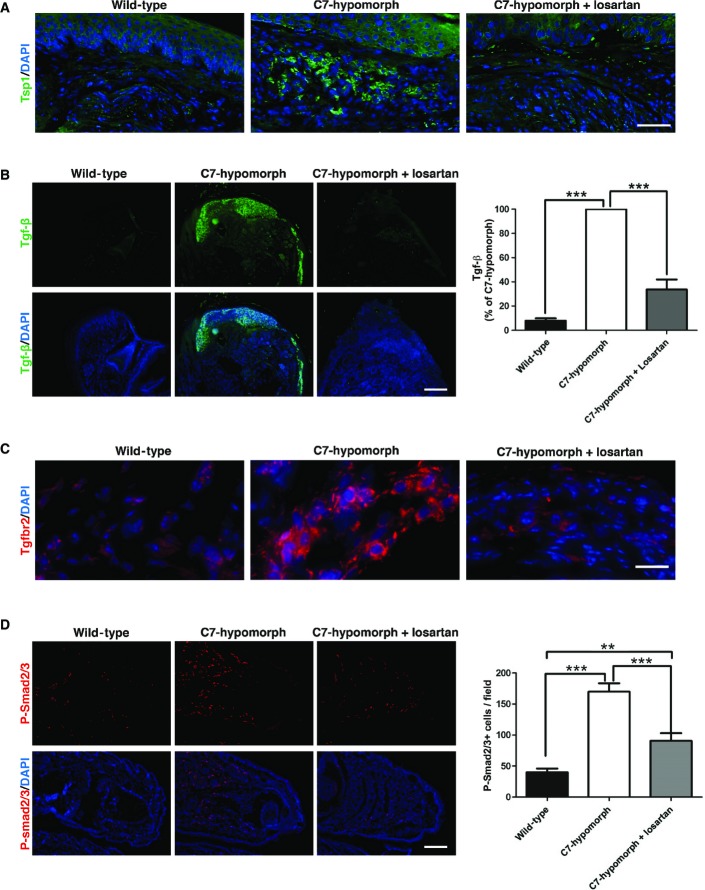
Losartan normalizes Tgf-β activity in C7-hypomorphic mice A–D C7-hypomorphic mice were treated with losartan for 7 weeks, and the forepaws of age-matched untreated, losartan-treated, and wild-type mice were subjected to immunofluorescence staining with antibodies to active thrombospondin 1 (Tsp1, green) (A), active Tgf-β1 (green) (B), TGF-β receptor II (Tgfbr2, red) (C), and phospho-Smad2 and 3 (P-Smad2/3, red) (D). The nuclei were counterstained with DAPI (blue). Images were acquired with a 20× objective (scale bar* *=* *100 μm) (A), with a 4× objective (scale bar* *=* *200 μm) (B, D), and with a 40× objective (scale bar* *=* *50 μm) (C). The bar graphs on the right show quantification of the stainings in the left panel of (B) and (D). Paired values were normalized to the staining intensity of untreated C7-hypomorphic paws, which were set to 100%. Values represent mean ± S.E.M., *n *=* *6, paired Student’s *t*-test, ***P-value wild-type vs. C7-hypomorph receiving no treatment* *=* *0.0004; ***P-value losartan treatment vs. no treatment* *=* *0.0005 (B). Values represent mean ± S.E.M., *n* ≥ 7, unpaired *t*-test with Welch’s correction used, ***P-value wild-type vs. C7-hypomorph < 0.0001; ***P-value C7-hypomorph + losartan vs. C7-hypomorph* *=* *0.0002; **P-value wild-type vs. C7-hypomorph + losartan* *=* *0.0016 (D).

Losartan can attenuate Tgf-β signaling through multiple mechanisms. By inhibiting angiotensin II type 1 receptor signaling, it reduces the expression of activators of latent Tgf-β, such as Tsp1 (Murphy-Ullrich & Poczatek, 2000), and the expression of Tgf-β ligands and receptors (Loeys, 2015). In the C7-hypomorphic mice, losartan treatment effectively lowered the levels of Tsp1 (Fig3A), Tgf-β1, and its cognate receptor Tgfbr2 in the forepaws (Fig3B and C). Losartan treatment also reduced the elevated levels of circulating Tgf-β1 (Supplementary Fig S3A). These changes attenuated Tgf-β downstream signaling, as seen by staining for phosphorylation of the canonical Tgf-β downstream signaling effector molecules Smad2/3 (Fig3D).

**Figure 4:**
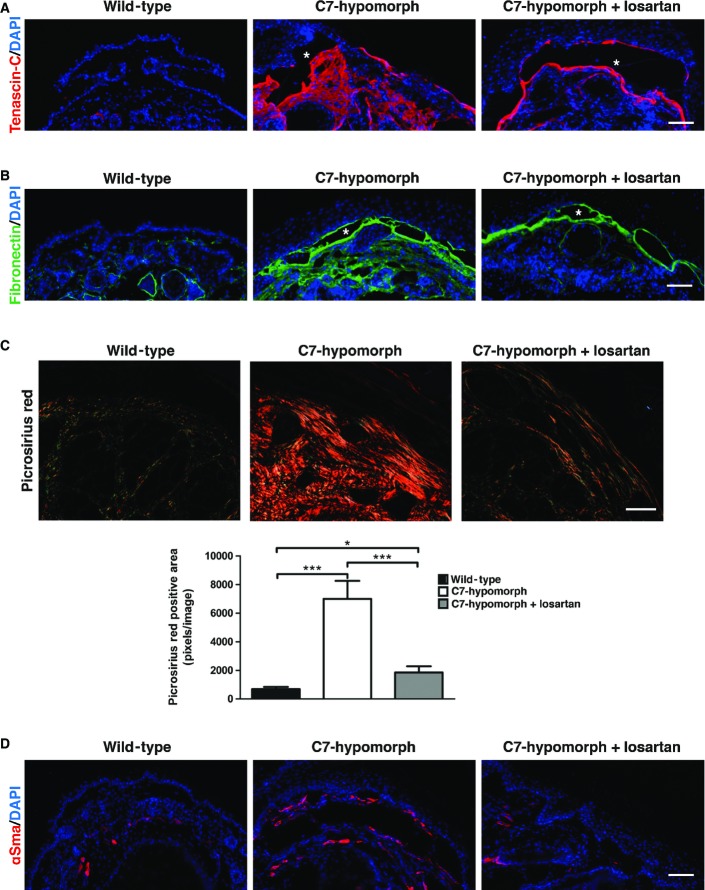
Reduced fibrotic remodeling in losartan-treated C7-deficient forepaws C7-hypomorphic mice were treated with losartan for 7 weeks, and the forepaws of age-matched untreated, losartan-treated, and wild-type mice were subjected to immunofluorescence staining with antibodies to fibrosis markers (A, B) and to picrosirius red staining (C).
A, B Tenascin-C (red) (A), fibronectin (green) (B). The nuclei were visualized with DAPI (blue). Images acquired with a 20× objective, scale bar* *=* *100 μm. Note that losartan did not completely abolish the staining, but effectively limited fibrosis to the site of initial tissue damage, that is, adjacent to the dermal–epidermal blistering (denoted by white asterisks) in C7-hypomorphic skin.

Reduction of Tgf-β activity greatly affected post-injury ECM remodeling in RDEB, specifically the expression of tenascin-C and fibronectin—two indicators of fibrosis. In the forepaws of 13-week-old wild-type mice, tenascin-C was solely present around hair follicles, but age-matched, C7-hypomorphic mice displayed intense tenascin-C expression throughout the dermis. A 7-week losartan treatment downregulated tenascin-C and restricted the expression to the site of original tissue injury in the C7-hypomorphic forepaws (Fig4A). Fibronectin was present at low levels in the dermis of uninjured wild-type mice, but strongly increased in the forepaws of C7-hypomorphic mice. Losartan treatment efficiently reduced the amount of fibronectin in C7-deficient forepaws (Fig4B).

C Picrosirius red staining and visualization of the collagen fibers under cross polarizing light. Under this light, thin fibers appear green and thick rigid collagen bundles orange-red. The staining revealed significantly reduced collagen fiber size in losartan-treated skin, indicating softer tissue similar to wild-type skin. Below, the bar graph shows quantification of picrosirius red-positive areas, *n* ≥ 19 areas quantified, values represent mean ± S.E.M. Unpaired *t*-test with Welch’s correction, ***P-value wild-type vs. C7-hypomorph < 0.0001; ***P-value C7-hypomorph + losartan vs. C7-hypomorph* *=* *0.0004; *P-value wild-type vs. C7-hypomorph + losartan* *=* *0.0189. Images acquired with a 20× objective, scale bar* *=* *50 μm.

D Immunofluorescence staining of forepaws as above with an antibody to αSma (red). αSma is present both around blood vessels and in myofibroblasts. Note the increase of αSma^+^ myofibroblasts in C7-hypomorphic paws and reduced number of αSma^+^ cells in losartan-treated C7-hypomorphic forepaws. Images acquired with a 20× objective, scale bar* *=* *100 μm.

During progressive fibrosis, remodeling of the ECM increases tissue stiffness. An important process in this context is cross-linking of collagens to thick and rigid bundles. TGF-β stimulates the expression of both collagen I and the enzymes involved in posttranslational processing of collagens such as BMP-1/mTolloid proteinases and lysyl oxidases (Lee *et al*, 1997; Uzel *et al*, 2001). To gain information on how losartan treatment affected biomechanically relevant ECM remodeling in the skin, picrosirius red staining was employed to assess collagen fiber sizes. Low staining intensity indicates normal fiber diameter, and bright staining intensity correlates with increased fiber diameter. In wild-type forepaws, most collagen fibers were thin, but in the forepaws of untreated C7-hypomorphic mice, the diameter was drastically increased (Fig4C). Intriguingly, losartan treatment effectively abolished fibril thickening (Fig4C), indicating that the drug inhibits progressive tissue stiffening in injured C7-deficient skin. This was corroborated by assessment of αSma-positive myofibroblasts. The conversion of fibroblasts to myofibroblasts is jointly promoted by increased tissue stiffness and TGF-β signaling (Hinz, 2009). αSma-positive myofibroblasts were plentiful in untreated C7-hypomorphic paws (Fig4D), but losartan treatment effectively limited the fibroblast–myofibroblast conversion (Fig4D).

**Figure 5:**
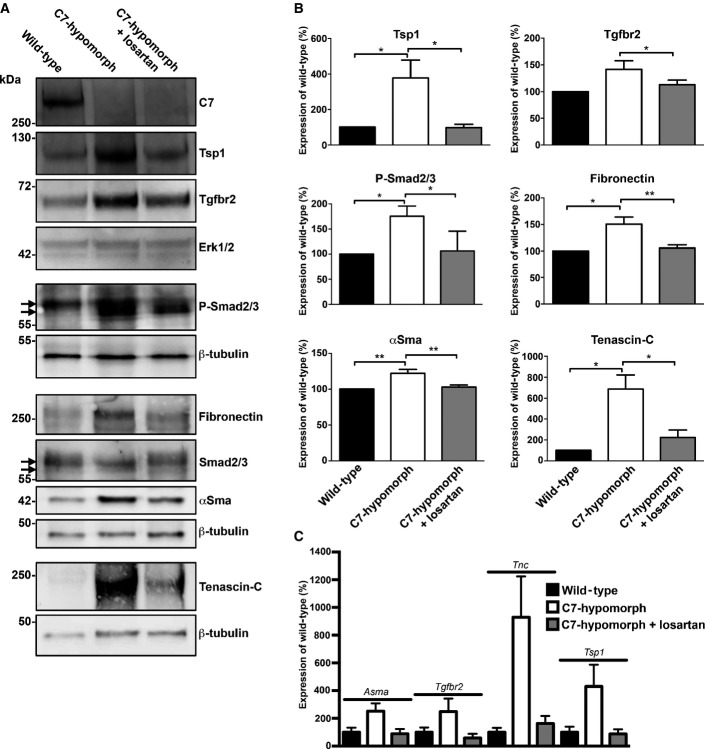
Long-term losartan treatment reduces Tgf-β signaling and expression of fibrosis-associated proteins in C7-deficient forepaws Representative Western blots of whole forepaw skin lysates from age-matched wild-type, untreated C7-hypomorphic, and C7-hypomorphic mice treated with losartan for 7 weeks, probed for proteins analyzed in Figs3 and 4. Erk1/2 or β-tubulin was used as a loading control. While losartan did not increase the expression of C7, it effectively attenuated fibrosis by reducing Tgf-β signaling and subsequent Tgf-β-regulated protein expression. Arrows point to bands corresponding to Smad2 and Smad3.

To further support the protective effect of losartan treatment on fibrotic progression in RDEB detected by immunohistological analyses, we performed Western blots on whole-skin lysates from forepaws. Notably, the Western blots confirmed the immunohistological observations of Tsp1, Tgfbr2, P-Smad2/3, fibronectin, αSma, and tenascin-C (Fig5A and B). To gain insight on at which level losartan interference with angiotensin II type 1 receptor signaling regulated the profibrotic gene expression, we performed qPCR analyses. We had already shown that losartan treatment reduced *TGFB1* transcripts, and the same had been reported for fibronectin in other fibrotic conditions (Gay-Jordi *et al*, 2013). Our analyses revealed that abundance differences of all analyzed proteins were also subject to transcriptional regulation (Fig5C), which is in line with *in vitro* findings (Wolf *et al*, 1999; Naito *et al*, 2004). Collectively, these data demonstrate that limiting TGF-β activity through antagonizing the angiotensin II type 1 receptor by losartan effectively ameliorates the RDEB phenotype *in vivo*.

Densitometric quantification of Western blots as in (A) from multiple different mice per group (*n* ≥ 3). Expression was normalized to a loading control (Erk1/2 or β-tubulin) and protein level expressed as percentage of wild-type. Values represent mean ± S.E.M. The data were analyzed by Student’s paired *t*-test; *P*-values wild-type vs. C7-hypomorph: Tsp1 *P =* *0.0490, Tgfbr2 *P *=* *0.0812, P-Smad2/3 *P =* *0.0346, fibronectin *P =* *0.0115, αSma *P =* *0.0038, tenascin-C *P =* *0.0228; *P*-values C7-hypomorph + losartan vs. C7-hypomorph: Tsp1 *P =* *0.0415, Tgfbr2 *P =* *0.0483, P-Smad2/3 *P =* *0.0272, fibronectin **P =* *0.0025, αSma **P =* *0.0038, tenascin-C *P =* *0.0134; *P*-values wild-type vs. C7-hypomorph + losartan: all not significant.

Quantitative real-time PCR (qPCR) analysis of RNA isolated from whole forepaw skin of mice as in (A). Expression of *Asma* (*Acta2*), *Tgfbr2*, *Tsp1* (*Thsb1*), and *Tnc* normalized to the expression of *Gapdh* and shown as the percentage of wild-type expression. Losartan treatment downregulated the expression of all four genes that were elevated in untreated C7-hypomorphic mouse paws. The reduction of *Asma*, *Tgfbr2*, and *Tsp1* did not reach statistical significance in one or two conditions due to large variation in the samples. Values represent mean ± S.E.M., unpaired *t*-test with Welch’s correction used; *P*-values wild-type vs. C7-hypomorph: *Asma P *=* *0.059, *Tgfbr2 P *=* *0.17*, Tnc* *P =* *0.019, *Tsp1 P *=* *0.098; *P*-values C7-hypomorph + losartan vs. C7-hypomorph: Asma *P =* *0.048, *Tgfbr2 P *=* *0.089*, Tnc* *P =* *0.028, *Tsp1 P *=* *0.087; *P*-values wild-type vs. C7-hypomorph + losartan: *Asma P *=* *0.81, *Tgfbr2 P *=* *0.36*, Tnc P *=* *0.33, *Tsp1 P *=* *0.81; *n* ≥ 5 different paws per group. Source data are available online for this figure.

### Global proteomics reveals injury repair and inflammation as targets of losartan

**Figure 6:**
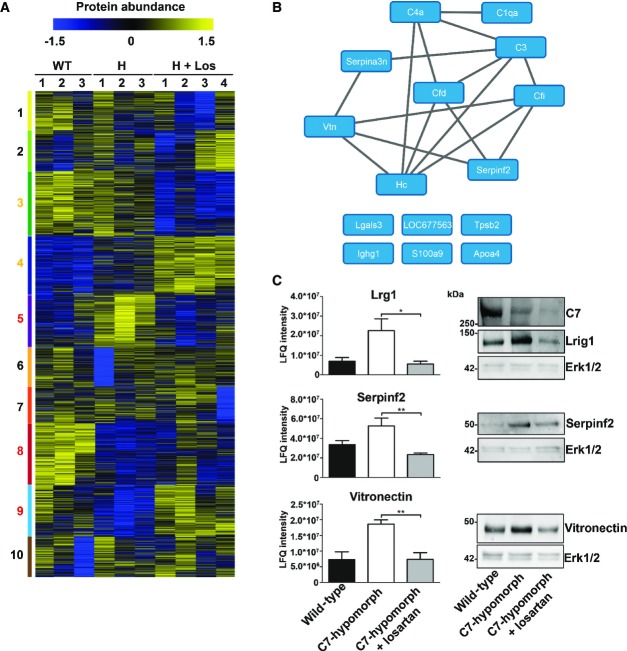
Proteomics analysis reveals losartan-mediated reversion of early fibrotic changes in RDEB Heat map of cluster analysis of protein abundances determined by label-free quantification mass spectrometry. Extracted ion currents were used to determine protein abundances, and respective intensities were log2-transformed and normalized (*z*-score). Samples were clustered hierarchically, and protein abundances were clustered by k-means. Cluster sizes are indicated by color code on the left. Clusters 3, 4, 5, 8, and 9 highlight losartan-induced changes. The general patterns of protein abundance in clusters 5, 8, and 9 are similar in wild-type and losartan-treated C7-hypomorphic mice and thus contain downstream targets of losartan involved in RDEB disease progression. WT, wild-type; H, hypomorphic; H + Los, losartan-treated C7-hypomorphic skin.

To understand how losartan-mediated reduction of TGF-β activity influenced molecular processes underlying disease progression in RDEB in general, we performed unbiased mass spectrometry-based proteomics analyses of whole skin of wild-type, C7-hypomorphic, and losartan-treated C7-hypomorphic mice. Back skin, which is protected from strong frictional damage by the fur, was chosen for the analyses since it represents an early stage of soft tissue fibrosis. Ten tissue specimens, three from wild-type, three from C7-hypomorphic, and four from losartan-treated C7-hypomorphic mice, were lysed in SDS buffer, proteins were separated by SDS–PAGE and digested in-gel, and the resulting peptide fractions were analyzed by label-free, quantitative mass spectrometry. In total 5,038 proteins were identified, of which 4,028 could be quantified in minimally one specimen sample. Of these, 2,242 were present in all samples and employed for further analysis (Supplementary Table S1). To identify groups of co-regulated proteins, the common proteins were clustered. The dataset was separated into 10 clusters of similar size representing the major regulation profiles (clusters 1–10; Fig6A).

Proteins from cluster 5 carrying GO terms related to inflammation were short-listed and analyzed on potential interactions using default settings in STRING DB (confidence score 0.4) (Szklarczyk *et al*, 2015).

Bar graphs show abundance of selected representative proteins in cluster 5 that were normalized by losartan treatment. Shown to the left are the mean ± S.E.M. of the normalized protein abundance (LFQ intensity) of groups of individual mice corresponding to wild-type, untreated, and losartan-treated C7-hypomorphic mice as indicated in the figure. Unpaired *t*-test was used to calculate significance. Abundance of Lrg1: *P-value C7-hypomorphic vs. losartan-treated C7-hypomorphic mice* *=* *0.022; abundance of serpin f2: **P-value untreated C7-hypomorphic vs. losartan-treated C7-hypomorphic mice* *=* *0.0084; abundance of vitronectin: **P-value untreated C7-hypomorphic vs. losartan-treated C7-hypomorphic mice* *=* *0.0094. Right, validation of proteomics analysis by Western blotting of independent biological replicates. Representative Western blots of skin lysates from wild-type, untreated C7-hypomorphic, and 7-week losartan-treated C7-hypomorphic mice not used for proteomics. Blots were probed with antibodies against proteins as indicated. Erk1/2 and β-tubulin served as loading controls. The analysis shows that there is a good correlation between proteomics data and abundance detected by Western blotting (*n* = 3 per group). Source data are available online for this figure.

The analysis revealed remarkable, global effects of losartan treatment on C7-deficient back skin. Losartan normalized elevated Tsp1 abundance, although the changes did not reach statistical significance due to high levels of variation in all three groups (Supplementary Table S1). Clusters 3 and 4 were related to the effects of losartan treatment, but not to RDEB disease progression, as wild-type and C7-hypomorphic samples were regulated similarly. These clusters contained proteins related to intracellular processes such as metabolism, transcription, and RNA processing (Supplementary Table S2). Proteins in clusters 5, 8, and 9 displayed aberrant abundance resulting from loss of C7, which was normalized by losartan treatment. This was most striking in clusters 5 and 9. Gene ontology (GO) enrichment analysis indicated that cluster 9 was abundant in proteins involved in ubiquitin and ubiquitin-like modifier processing (Supplementary Table S2). Cluster 5 was significantly enriched in GO terms associated with tissue inflammation (e.g., antimicrobial, complement, and coagulation cascades, and innate immunity; *P* < 0.05 BH corrected, Supplementary Table S2). Underlying proteins were analyzed on potential interactions to uncover responsible deregulated cellular pathways; we could construct a network consisting of nine proteins important for complement activation and immune and inflammatory responses (Fig6B). Interestingly, of these proteins, serpin f2 (α2-antiplasmin or plasmin inhibitor) has also been shown to promote TGF-β expression and experimentally induced fibrosis (Kanno *et al*, 2007). Losartan effectively limited serpin f2 abundance in C7-hypomorphic back skin as shown by Western blot and quantitative mass spectrometry (Fig6C). Also, vitronectin expression is linked to inflamed injured tissue (Seiffert, 1997; Tsuruta *et al*, 2007). Vitronectin stimulates dermal healing (Jang *et al*, 2000), is transiently upregulated during scar formation, and has been identified as a marker of hepatic fibrosis (Koukoulis *et al*, 2001; Montaldo *et al*, 2014). It was highly abundant in C7-deficient skin. Again, losartan treatment attenuated its abundance (Fig6C). The murine gene encoding vitronectin is not responsive to Tgf-β, but to the pro-inflammatory cytokine interleukin-6 (IL-6) (Seiffert *et al*, 1996), revealing a role of IL-6 in RDEB skin. In line with this, analysis of IL-6 in serum demonstrated significantly increased levels in RDEB mice and patients (Supplementary Fig S3A and B). Losartan significantly lowered the circulating Il-6 levels in RDEB mice (Supplementary Fig S3A).

Cluster 5 was also enriched in proteins connected to the development of fibrosis and regulating TGF-β signaling and expression. For example, leucine-rich alpha-2-glycoprotein 1 (Lrg1) is known to interact with the TGF-β co-receptor endoglin and to modulate TGF-β signaling (Wang *et al*, 2013a). Its expression is connected with TGF-β1 and TGF-β receptor II expression (Sun *et al*, 1995). Lrg1 was highly increased in C7-hypomorphic back skin, and losartan potently downregulated its abundance (Fig6C). Taken together, the comparison of protein abundance changes in C7-hypomorphic and wild-type skin underlines the role of fibrosis and TGF-β signaling in RDEB pathophysiology and points to a previously unappreciated role of pro-inflammatory factors among the molecules determining disease progression.

### Tissue inflammation in RDEB is reduced by losartan treatment

**Figure 7:**
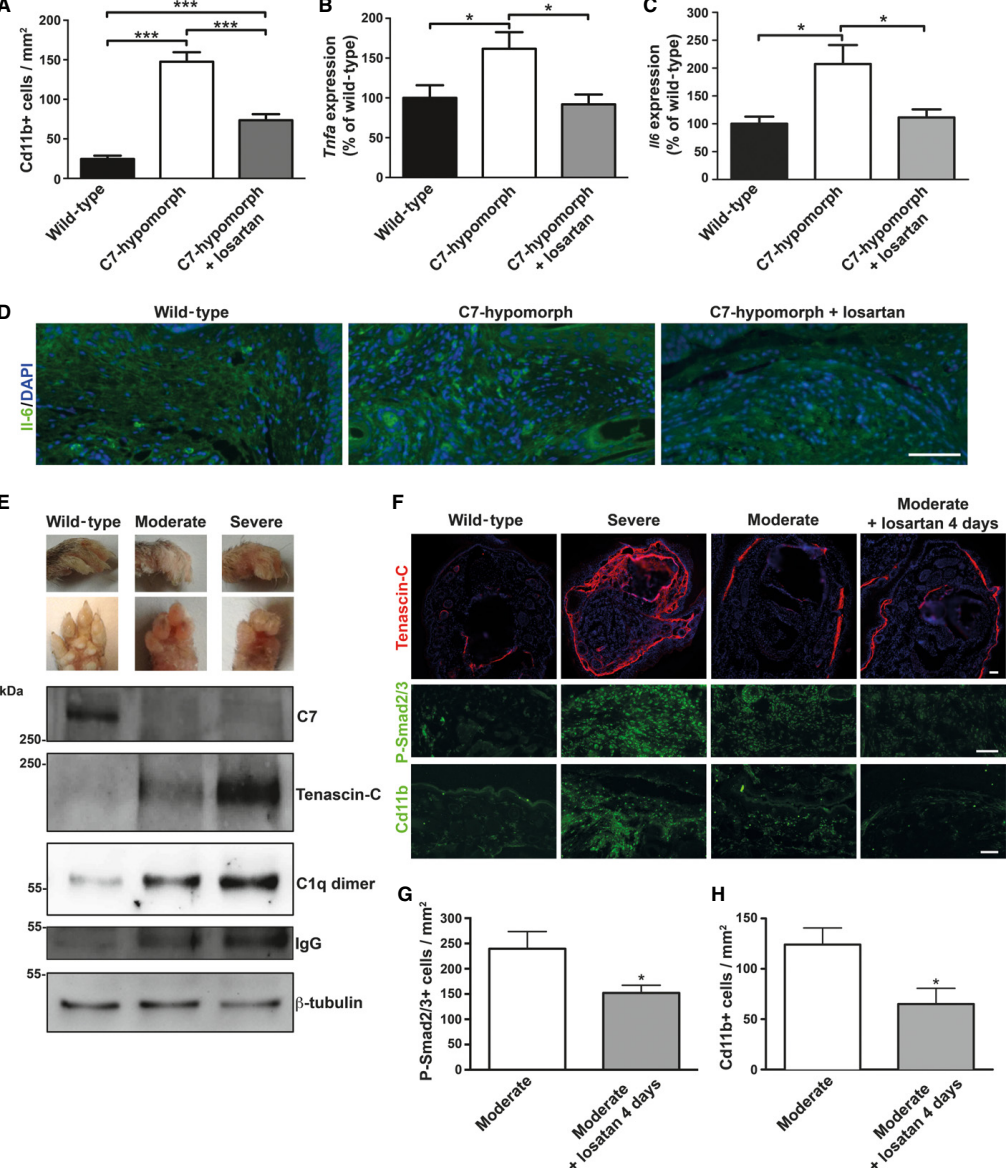
Tgf-β inhibition through losartan effectively relieves inflammation in RDEB A Quantification of Cd11b-positive cells shows that losartan treatment significantly reduced the number of these cells in C7-hypomorphic paws. Values represent mean Cd11b-positive cells per 1 mm^2^ ± S.E.M. Unpaired *t*-test with Welch’s correction was used, ***P-value wild-type vs. C7-hypomorph < 0.0001; ***P-value C7-hypomorph + losartan vs. C7-hypomorph* *=* *0.0008; ***P-value wild-type vs. C7-hypomorph + losartan < 0.0001 (*n* = 5). 

Next, we assessed Cd11b-positive cells as markers of inflammation in chronically damaged RDEB skin. In contrast to wild-type forepaw skin, the number of Cd11b-positive cells was strongly increased in untreated C7-hypomorphic paws. Losartan treatment significantly reduced the number of these cells, indicating that the drug alleviated inflammation (Fig7A). Similar, but milder, changes were observed in the back skin of C7-hypomorphic mice (Supplementary Fig S4).

B, C qPCR analysis of *Tnfa* and *Il6* mRNA expression in forepaws, normalized to the housekeeping gene *Gapdh*. Treatment with losartan significantly lowered the elevated expression of both genes in C7-hypomorphic paws. Values are expressed as percentage of expression in age-matched wild-type forepaws and represent mean ± S.E.M. Unpaired *t*-test with Welch’s correction used for data analysis. (B) *P-value wild-type vs. C7-hypomorph* *=* *0.033; *P-value C7-hypomorph +losartan vs. C7-hypomorph* *=* *0.015; *P*-value wild-type vs. C7-hypomorph + losartan* *=* *0.57, *n *=* *5. (C) *P-value wild-type vs. C7-hypomorph* *=* *0.69; *P-value C7-hypomorph + losartan vs. C7-hypomorph* *=* *0.029; *P*-value wild-type vs. C7-hypomorph + losartan* *=* *0.57, *n* = 5.

D Immunofluorescence staining of age-matched wild-type, untreated and losartan-treated C7-hypomorphic forepaws with an antibody to Il-6. The number of Il-6-positive bright cells (green) is clearly increased in untreated C7-deficient skin, and nearly normalized after 7-week losartan treatment. Nuclei visualized with DAPI. Images acquired with a 20× objective, scale bar = 100 μm.

E Correlation of tissue inflammation with disease progression in RDEB. Photographs of wild-type and moderately and severely affected C7-hypomorphic forepaws. These paws were processed for Western blotting shown below. The blots were probed with antibodies detecting C7, tenascin-C, C1q, and IgG. β-tubulin was used as a loading control. Shown for C1q is a dimeric form (Wing *et al*, 1993). Note the difference between moderately and severely affected paws. The C7 expression does not differ, but the severely affected paw with more extensive fibrosis and tenascin-C expression indicating remodeling also displays more tissue inflammation, as shown by increased C1q and IgG levels.

F Short-term losartan treatment rapidly alleviated inflammation through reduction of Tgf-β signaling. Sections of forepaws as in (E) plus sections of forepaws of C7-hypomorphic mice with moderately affected paws treated with losartan for 4 days were stained for tenascin-C, P-Smad2/3, and Cd11b. There is a clear correlation between the extent of fibrosis, as revealed by tenascin-C staining; Tgf-β signaling, as detected by P-Smad2/3; and inflammation, as indicated by Cd11b^+^ cells in the C7-hypomorphic forepaws. The 4-day losartan treatment efficiently reduced Tgf-β signaling (P-Smad2/3) and inflammation (Cd11b^+^) in moderately affected paws, as compared to untreated C7-hypomorphic paws with similar degree of fibrosis. Collectively, the data show that TGF-β-mediated inflammation is a driver of disease progression in RDEB and a major losartan target. Scale bars* *=* *100 μm.

G, H Quantification of stainings of moderately affected C7-hypomorphic forepaws with or without a 4-day losartan treatment as in (F). Positively stained cells were quantified after background had been subtracted by applying equal threshold. The values were expressed as positive cells per mm^2^. Values represent mean ± S.E.M. Data were analyzed with unpaired *t*-test with Welch’s correction. (G) P-Smad2/3 staining, *P =* *0.033. (H) Cd11b^+^ cells, *P =* *0.027. *n* = 3 different mice per group. Source data are available online for this figure.

Since proteomics analysis clearly indicated that a major effect of losartan on RDEB disease progression was mediated by reduced inflammatory activity, it was of interest to assess changes in the inflammatory response. To this end, the expression of pro-inflammatory cytokines in RDEB mice was analyzed after 7 weeks of losartan treatment. *Tnfa* gene expression was significantly upregulated in C7-hypomorphic forepaws compared to wild-type mice and, importantly, treatment with losartan effectively normalized *Tnfa* expression (Fig7B). C7-hypomorphic mice also displayed increased serum level of Tnf-α, which was significantly reduced by losartan (Supplementary Fig S3A). Notably, *Il6* followed the same pattern as *Tnfa*. Its gene expression was elevated in untreated C7-hypomorphic paws, and losartan reduced it to wild-type levels (Fig7C). The differences on the mRNA level were validated with immunofluorescence staining, which revealed an increased number of Il-6-positive cells in untreated RDEB forepaws and a decrease after losartan treatment (Fig7D).

To affirm the causative link of TGF-β signaling, tissue inflammation, and disease severity in RDEB, we devised a strategy where we analyzed age-matched paws from C7-hypomorphic mice differently affected with disease. Moderately affected mice had forepaws with four clearly visible digits, and severely affected mice displayed forepaws with shorter digits, extensive digit fusion, and fewer than three digits (Fig7E). The paws were subjected to biochemical and histological analysis for markers of inflammation and fibrosis. The dermis of both groups of C7-hypomorphic mice showed increased Tgf-β signaling, as determined by P-Smad2/3, and more signs of tissue inflammation than that of wild-type mice (Fig7E and F). Western blotting confirmed the increased expression of C1q in C7-hypomorphic skin found by quantitative mass spectrometry analysis (Figs6B and 7E). Importantly, Tgf-β signaling and inflammation were less in moderately affected paws than in severely affected paws. Notably, this correlated with tenascin-C expression (Fig7E and F). Thus, there was a clear relationship between Tgf-β signaling, inflammation, and extent of fibrosis, supporting the link between inflammation and disease progression in RDEB.

Lastly, since it was possible that the losartan-mediated effect on reduction of inflammation was not a direct effect, but a consequence of reduced fibrotic remodeling in C7-hypomorphic dermis, we analyzed the effect of short-term losartan treatment. C7-hypomorphic mice with moderately affected paws received losartan for 4 days. This time was sufficient to significantly reduce dermal Tgf-β signaling without affecting fibrotic deposition, as compared to similarly affected untreated paws (Fig7F and G). Importantly, the short-term losartan treatment also effectively promoted resolution of inflammatory infiltrates (Fig7F and H), establishing a close link between losartan-induced silencing of Tgf-β signaling and clearance of inflammation. Collectively, these results indicate that losartan-mediated attenuation of TGF-β-driven inflammation and immune dysregulation substantially contribute to phenotypic improvement in RDEB.

## Discussion

Here, we report a new evidence-based approach to attenuate the RDEB phenotype using a repurposed drug. So far, most efforts on therapy development for RDEB have concentrated at reintroducing C7 into the skin using gene-, cell- or protein-based therapy strategies (Hsu *et al*, 2014). However, given a host of challenges with such approaches, clinical implementation is likely to be years away. Therefore, we took an alternative approach aimed at amelioration of symptoms, instead of cure, by targeting mechanisms downstream of C7. The rationale is based on the findings that TGF-β activity is greatly increased both in the skin and in the circulation of patients with RDEB. This is presumably a consequence of the combination of altered dermal tissue architecture that releases matrix-bound TGF-β, and of inflammation following tissue damage. We chose losartan as a TGF-β inhibitor, since it is an approved drug; clinical implementation could follow relatively fast in case of positive preclinical findings.

In addition to the immediately visible signs of C7 deficiency, skin fragility, and trauma-induced blister formation, secondary disease mechanisms play a significant role in genotype–phenotype correlations of RDEB. The present study concentrated in elucidation of the molecular mechanisms and changes the concept of RDEB as a cutaneous mechanobullous disorder into that of a systemic fibrotic disease and shows that restraining TGF-β activity markedly improves the phenotype at tissue, cellular, and molecular levels, that is, diminishes inflammation, excessive ECM accumulation, and tissue stiffness. Clinically, this is reflected by substantially reduced fibrotic changes of the skin.

Unbiased global proteomics analyses were used to connect the obvious phenotypic improvement of RDEB upon losartan treatment to molecular events. Proteomics analysis of the back skin of adult C7-hypomorphic mice, which displayed early fibrotic changes, provided insights into processes associated with initial stages of RDEB scarring. These turned out to be more complex than anticipated. On the one hand, the analysis uncovered highly abundant proteins in the skin that were connected to TGF-β signaling, tissue damage, or early-stage fibrotic changes, validating the approach of TGF-β blockage to delay fibrosis in RDEB. On the other hand, the role of inflammation in RDEB pathology was underscored. Some of the abundant proteins in RDEB skin were associated with both innate and adaptive immunity and indicated involvement of hitherto unappreciated cellular and molecular mechanisms. For example, elevated vitronectin levels were reduced by losartan. The fact that the murine gene is not responsive to TGF-β, but to IL-6 (Seiffert *et al*, 1996), indicates that this pro-inflammatory cytokine is involved in the pathology of RDEB. Indeed, losartan significantly lowered the IL-6 expression in wounded RDEB mouse paws. Another example of an increased protein whose abundance was reduced by losartan is galectin-3 (Lgals3) that is known to be increased in autoimmune disorders and tissue inflammation (Henderson & Sethi, 2009; Radosavljevic *et al*, 2012). Lastly, losartan reduced the expression of TNF-α in RDEB paws in line with a preliminary suggestion that TNF-α is involved in the pathophysiology of RDEB (Gubinelli *et al*, 2010), and additionally, TNF-receptor signaling has recently been implicated to promote formation of wound-induced SCCs (Hoste *et al*, 2015). These observations place inflammation before extensive fibrotic development in RDEB and indicate that immune/inflammatory reactions represent a major target of losartan.

TGF-β plays a complex-, context-, and concentration-dependent role in inflammation (Kim *et al*, 2006; Dietz, 2010), and it can act in both pro- and anti-inflammatory manner. Bursts of TGF-β activity, as after tissue injury in RDEB, stimulate macrophage recruitment, whereas prolonged TGF-β stimulus decreases their migration (Kim *et al*, 2006). In RDEB, losartan-mediated TGF-β inhibition effectively alleviated signs of tissue inflammation.

Context-dependent complexity of secondary disease mechanisms has been observed also in other inherited disorders with structural proteins at fault. An illustrative example is osteogenesis imperfecta (OI), caused by mutations in collagen I, in which three different mechanisms can contribute to the biological phenotype: structural alterations in the secreted collagen, unfolded protein response, and TGF-β dysregulation (Kojima *et al*, 1998; Lisse *et al*, 2008; Grafe *et al*, 2014). In OI, changed bioavailability of TGF-β results from impaired protein–protein interactions in the ECM. Mutated collagen I cannot efficiently bind decorin, a strong TGF-β ligand, and unbound TGF-β activates macrophage lineage-derived osteoclasts which dismantle the bone (Grafe *et al*, 2014). Intriguingly, decorin is also a natural modulator of the RDEB phenotype (Odorisio *et al*, 2014) and, in analogy to OI, increased TGF-β activity contributes to disease progression in RDEB.

Clinically, losartan treatment is promising for RDEB. A major limitation is, however, that it does not reduce skin blistering, the primary manifestation of the disorder. However, in light of current lack of causal treatment options, the severity of RDEB calls for urgent translation of potential therapies to attenuate symptoms and improve functionality and quality of life of affected individuals (Davila-Seijo *et al*, 2014). Given its long-term safe use in children and adults (Pees *et al*, 2013), losartan seems an ideal repurposed drug for the first disease-modulating therapy of RDEB. The delay of mitten deformity formation by a relatively short 7-week treatment in the mice corresponds roughly to a delay of 2 years in patients with severe RDEB (Fritsch *et al*, 2008), suggesting that continuous or repeated treatments at regular intervals will generate beneficial long-term effects in patients. Apart from the efficacy in inhibiting soft tissue fibrosis, reducing inflammation is likely to alleviate pain associated with the blisters and wounds. It can be surmised that the earlier the treatment with losartan can be initiated, the better the protective effects of the therapy will be. Since the action spectrum of losartan on TGF-β signaling is broad, future studies will answer whether the use of compounds directed at specific targets in the TGF-β pathway may be more effective. Ultimately, the overall efficacy of losartan or its analogs for RDEB, and optimal treatment and dosage regimens have to be determined in clinical trials.

In conclusion, this study changes the concept of RDEB physiopathology by demonstrating that it is a systemic, chronic inflammatory fibrotic disease, and by describing evidence-based efficacy of losartan treatment on events related to TGF-β-mediated dysregulation of inflammation and ECM remodeling. Given the large contribution of these processes to RDEB, our findings show that losartan and/or analogous compounds will provide urgently needed attenuation of symptoms in RDEB until a cure becomes possible.

## Materials and Methods

### Studies using animals and patient material

Studies using mice were approved by the regional review board (Regierungspräsidium Freiburg, Freiburg, Germany; approval no. 35/9185.81/G10-118). The C7-hypomorphic mice (Fritsch *et al*, 2008) on mixed C57BL/6 129sv background and wild-type littermates were kept in a pathogen-free facility at the University of Freiburg and given food and water *ad libitum*; C7-hypomorphic mice also had access to a special soft-food diet (Fritsch *et al*, 2008). Mice used for the experiments were kept with cagemates. After weaning, the C7-hypomorphic mice were followed weekly for appearance of forepaw deformities. At the first sign of toe loss, the mice were randomized into control or active treatment groups. Sample size for the studies was estimated after consultation with the Institute for Medical Biometry and Statistics, University of Freiburg, using a log-rank test with a power of 80% and for a *P*-value < 5%. The control group received regular water, and the active treatment group was given 0.6 g losartan (losartan potassium; Sandoz, Istanbul, Turkey) per liter drinking water (Habashi *et al*, 2006). By estimating the average individual water consumption, the daily dose per mouse was approximated to 200 mg/kg body weight. This value represents the average consumption per mouse regardless of genotype and not accounting for differences in consumption due to genotype. The water consumption was estimated in cages with mixed C7-hypomorphic and wild-type mice, in most cages 1 C7-hypomorphic and 4 wild-type mice or 2 C7-hypomorphic and 3 wild-type mice. Water bottles were weighed before attachment to cages, and after 1 day, to account for fluid loss from evaporation, reference bottles were attached to empty cages. The weight loss was converted to volume and divided per the number of mice in the cages to approximate the average daily water consumption per mouse. The mice were monitored daily and photographed weekly after treatment start. Fourteen mice were followed per group (8 females and 6 males for the control group and 7 females and 7 males for the losartan-treated group). At treatment start, the mice were on average 39 ± 7 days old in the control group and 38 ± 8 days old in the losartan-treated group. After 7 weeks, the mice were euthanized with CO_2_ inhalation, organs were collected and embedded in Tissue-Tek CRYO-OCT and snap-frozen or fixed in 10% formalin and paraffin embedded.

Progression of forepaw mutilation was quantified using ImageJ (NIH, Bethesda, MD, USA); both forepaws were used for measurements. The length of the two middle digits was measured and normalized to the width of a fixed zone on the wrist that is not altered by fibrosis. Changes in the digit:wrist ratio were followed and normalized to the digit:wrist ratio at the start of the experiment which was set to 100%.

Paraffin-embedded specimens from normal human skin, acute wounds, chronic venous ulcers, and RDEB wounds were sectioned and deparaffinized, and antigen retrieval was performed with citrate buffer.

Studies using patient material were approved by the ethics committee of the University of Freiburg (approval no. 293/14). The patients gave informed consent before their participation, and the study was performed in accordance with the Declaration of Helsinki.

### Cell culture

Dermal fibroblasts from normal human donors and patients with severe generalized RDEB were isolated and cultured as previously described (Sprenger *et al*, 2013) and designated NHF and RDEBF, respectively. For experiments with losartan, the optimal dose was determined by its ability to limit ERK phosphorylation in fibroblasts (Habashi *et al*, 2011). 10 μM was the concentration that maximally reduced phosphorylation without affecting cell survival. This concentration was used in all subsequent studies.

The fibroblasts were kept at 70–80% confluence and cultured with 50 mg/l ascorbic acid added fresh daily. A total of 10 μM losartan dissolved in growth medium was added for 24 or 48 h. Thereafter, proteins from cell layers and their ECM were extracted with RIPA buffer and analyzed by Western blotting as described below.

### PCR

RNA was extracted from fibroblasts by NucleoSpin RNA isolation kit (Macherey-Nagel) according to the manufacturer’s instructions. For isolation of RNA from the skin, forepaw skin was first carefully removed from paws and snap-frozen in liquid nitrogen, and 30 mg tissue was crushed with mortar and pestle. RNA was extracted with RNeasy Fibrous Tissue Kit (Qiagen) according to the manufacturer’s instructions. The RNA was reverse-transcribed to cDNA using First Strand cDNA Synthesis Kit (Thermo Fisher Scientific). qPCR analysis was performed with SYBR green labeling and a CFX96 Real-Time system (Bio-Rad). The primers used were as follows: *TGFB1F*: AAGTTGGCATGGTAGCCCTT; *TGFB1R:* CCCTGGACACCAACTATTGC; *GAPDHF*: CCCATCACCATCTTCCAG; *GAPDHR*: ATGACCTTGCCCACAGCC; *Il6F*: TGGTACTCCAGAAGACCAGAGG; *Il6R*: AACGATGATGCACTTGCAGA; *TnfaF*: ATGAGAGGGAGGCCATTTG; *TnfaR*: CAGCCTCTTCTCATTCCTGC; *Asma* (*Acta2*)*F*: GTTCAGTGGTGCCTCTGTCA; *Asma* (*Acta2*)*R*: ACTGGGACGACATGGAAAAG; *Tgfr2F*: TGTCGCAAGTGGACAGTCTC; *Tgfr2R*: GGACCATCCATCCACTGAAA; *TncF*: ATCCCTTCATCAGCAGTCCA; *TncR*: GCATCCGTACCAAAACCATC; *Tsp1* (*Thsb1*)*F*: GTCCACTCAGACCAGGGAGA; *Tsp1* (*Thsb1*)R: AAAGGTGTCCTGTCCCATCA; *GapdhF*: TTGATGGCAACAATCTCCAC; and *GapdhR*: CGTCCCGTAGACAAAATGGT.

### Antibodies and Western blotting

The following antibodies were used: mouse anti-human TSP1 clone A6.1, rabbit anti-P-SMAD2/3 (sc-11769R), rabbit anti-SMAD2/3 (FL-425), and goat anti-LRG1 (P-16) (Santa Cruz Biotechnology, Santa Cruz, CA, USA); rabbit anti-human collagen I (R1038X) (Acris Antibodies, Herford, Germany); rabbit anti-active TGF-β1 (Promega, Madison, WI, USA); mouse anti-human β-actin clone AC-15, mouse anti-human total ERK1/2 clone ERK-NP2, mouse anti-human P-ERK1/2 clone ERK-PT 115, rat anti-mouse tenascin-C clone 578, and mouse anti-human serpin F2 (mAb1470; R&D systems, Minneapolis, MN, USA); rabbit anti-human IL-6 (ab6672), rabbit anti-human fibronectin (ab2413), rabbit anti-human TGFBR2 (ab28382), and rabbit anti-β-tubulin (ab6046) (Abcam, Cambridge, UK); rabbit anti-P-SMAD3 (pS465/467) (138D4) (Cell Signaling, Danvers, MA, USA); rabbit anti-P-SMAD2 (pS423/425) (EP823Y) (Epitomics, Burlingame, CA, USA); rat anti-mouse Cd11b clone M1/70 (BD Biosciences, Heidelberg, Germany); rat anti-mouse C1q clone 7H8 (Hycult Biotech, Uden, the Netherlands); rabbit anti-collagen VII (234192) (Merck Millipore, Billerica, MA, USA); rabbit anti-vitronectin (NBP2-20866) (Novus Biologicals, Littelton, CO, USA); rabbit anti-SERPINF2 (orb101685) (Biorbyt, Cambridge, UK); Cy3-conjugated mouse anti-αSMA clone 2B1 (Sigma-Aldrich, St. Louis, MO, USA); HRP-conjugated goat anti-rabbit IgG and HRP-conjugated goat anti-mouse IgG (Jackson ImmunoResearch, West Grove, PA, USA); and Alexa 488- or 546-conjugated goat anti-rat, anti-rabbit, or anti-mouse IgG (Molecular Probes, Eugene, OR, USA).

Back skin and carefully isolated skin from forepaws were snap-frozen in liquid nitrogen and crushed with mortar and pestle. A total of 30 mg crushed tissue was directly added to 200 μl boiling hot 4× Laemmli loading buffer containing 8 M urea, boiled for 20 min, and loaded on 4–12% gradient Tris–glycine polyacrylamide gels. After separation, proteins were electrotransferred onto nitrocellulose membranes in Tris–borate–EDTA buffer. Membranes were blocked in 5% milk in TBS or 3% BSA in TBS and incubated with primary and secondary antibodies in blocking buffer. Blots were developed using ECL substrate (Thermo Scientific, Rockford, IL) and the Fusion SL system (Peqlab, Erlangen, Germany). Blots were densitometrically quantified using ImageJ, and to compare the expression in multiple mice analyzed on multiple blots, values were expressed as the percentage of wild-type after normalization to loading control.

For the analysis of circulating Tgf-β, Il-6, and Tnf-α, dot blots were performed. Mouse sera were diluted 1:10 in PBS and dotted on nitrocellulose membranes. The membrane was dried, wetted in TBS, and blocked with 5% milk in TBS. The membranes were probed with anti-TGF-β, anti-IL-6, or anti-TNF-α antibodies and then HRP-conjugated secondary antibody.

Sera of normal human controls and patients with genetically confirmed completely C7-deficient RDEB were diluted 1:10, boiled in Laemmli sample buffer, loaded and separated on a 4–20% gradient SDS–PAGE, and blotted to PVDF membranes for the analysis of TGF-β1 expression. Membranes were stained with Ponceau S (Sigma-Aldrich) to ensure equal loading.

### Collagen lattice contraction assay

Collagen lattice contraction assays were performed essentially as described (Odorisio *et al*, 2014). Fibroblasts were harvested by trypsinization and resuspended in DMEM containing 0.1% FCS. In each 12-well plate, 250,000 cells per well were mixed with 0.4 mg/ml rat tail collagen and DMEM with 0.1% FCS, and a final volume 10 μM losartan was added to the gels receiving losartan. The gel cell slurry was poured into the wells and allowed to gel at 37°C for 1 h in a cell incubator with 5% CO_2_. Then, 1 ml DMEM containing 0.1% FCS ± 10 μM losartan was added and the plate further incubated in the cell incubator for 2 h after which the edges were cut and gels released using a 200-μl pipette tip. Pictures were taken immediately after the release of the gels and after 24 h. Contraction of gels was quantified using ImageJ.

### Immunofluorescence analysis

Cryosections fixed in acetone or 4% paraformaldehyde, or paraffin sections after steps of rehydration and antigen retrieval with 0.5% pepsin or 10 mM sodium citrate were blocked in 4% BSA–PBS and stained with primary and secondary antibodies diluted in blocking buffer. Sections were analyzed with an Axioplan2 fluorescence microscope (Zeiss) and images captured with a black and white Axiocam camera. Images were captured using identical settings and exposure time and further processed using the Zeiss Zen 2010 software without alterations of signal ranges. Stainings were quantified with ImageJ either by directly measuring intensity of the fluorescent signal or by counting positive cells after the application of constant threshold and conversion to binary images.

### Histological analysis and picrosirius red staining

Paraffin-embedded mouse forepaws were sectioned and stained with H&E or EvG as previously described (Nystrom *et al*, 2013). Photographs were captured with an Axioplan2 microscope (Zeiss) equipped with a black and white Axiocam camera. For picrosirius red staining, paraffin sections were stained with Weigert’s hematoxylin for 8 min and counterstained with 0.1% picrosirius red (Direct Red 80; Sigma-Aldrich) for 1 h. The sections were serially imaged using Axioplan2 fluorescence microscope (Zeiss) equipped with an analyzer and polarizer and Axiocam camera. Images were captured using identical settings and exposure time. Quantification of collagen fibril density was performed on images taken with orthogonally oriented polarized light using ImageJ. 50 × 50 mm regions in each image at the dermal area, just below the epidermis, were selected for the quantification. A minimal threshold was set and maintained for all images within each experiment. The area (in pixel) of the brightness of threshold light was calculated from a minimum of 19 images per condition.

### Proteomics analysis

#### Sample preparation

Skin specimens were obtained from back skin of eight age-matched C7-hypomorphic (3), wild-type (3), and C7-hypomorphic mice treated with losartan for 7 weeks (2). Technical replicates were made from the losartan-treated skin resulting in four samples. After sacrifice, the back skin was shaved and snap-frozen. For protein extraction, 200 mg skin was thawed in PBS containing protease inhibitors (Roche). The specimen was carefully cleaned from adipose tissue, cut into small pieces, and homogenized in 2 ml lysis buffer (4% SDS in 0.1 M Tris–HCl, pH 7.6) with an ULTRA-TURRAX homogenizer. Lysed skin samples were heated in SDS–PAGE loading buffer, reduced with 1 mM DTT (Sigma-Aldrich) for 5 min at 95°C, and alkylated using 5.5 mM iodoacetamide (Sigma-Aldrich) for 30 min at 20°C. The protein mixtures were separated by 4–12% gradient SDS–PAGE (NuPAGE; Invitrogen). The gel lanes were cut into 10 equal slices, the proteins were in-gel digested with trypsin (Promega) (Shevchenko *et al*, 2006), and the resulting peptide mixtures were processed on STAGE tips (Rappsilber *et al*, 2007) and analyzed by LC-MS/MS.

#### Mass spectrometry

Mass spectrometry (MS) measurements were performed on an LTQ Orbitrap XL mass spectrometer (Thermo Fisher Scientific) coupled to an Agilent 1200 nanoflow HPLC system (Agilent Technologies GmbH, Waldbronn, Germany) (Zarei *et al*, 2013). HPLC-column tips (fused silica) with 75 μm inner diameter (New Objective, Woburn, MA, USA) were self-packed with Reprosil-Pur 120 ODS-3 (Dr. Maisch, Ammerbuch, Germany) to a length of 20 cm. Samples were applied directly onto the column without a precolumn. A gradient of A (0.5% acetic acid (high purity; LGC Promochem, Wesel, Germany) in water) and B (0.5% acetic acid in 80% acetonitrile (LC-MS grade; Wako, Germany) in water) with increasing organic proportion was used for peptide separation (loading of sample with 2% B; separation ramp: from 10–30% B within 80 min). The flow rate was 250 nl/min, and for sample application, 500 nl/min. The mass spectrometer was operated in the data-dependent mode and switched automatically between MS (max. of 1 × 10^6^ ions) and MS/MS. Each MS scan was followed by a maximum of five MS/MS scans in the linear ion trap using normalized collision energy of 35% and a target value of 5,000. Parent ions with a charge state from *z* = 1 and unassigned charge states were excluded for fragmentation. The mass range for MS was *m*/*z* = 370–2,000. The resolution was set to 60,000. MS parameters were as follows: spray voltage 2.3 kV; no sheath and auxiliary gas flow; ion-transfer tube temperature 125°C.

#### Identification of proteins and protein ratio assignment using MaxQuant

The MS raw data files were uploaded into the MaxQuant software version 1.4.1.2 (Cox and Mann, 2008) for peak detection, generation of peak lists of mass error corrected peptides, and database searches. A full-length UniProt mouse database additionally containing common contaminants such as keratins and enzymes used for in-gel digestion (based on UniProt mouse FASTA version September 2012) was used as reference. Carbamidomethylcysteine was set as fixed modification; methionine oxidation and protein amino-terminal acetylation were set as variable modifications and label-free was chosen as quantitation mode. Three miss cleavages were allowed, enzyme specificity was trypsin/P, and the MS/MS tolerance was set to 0.5 Da. The average mass precision of identified peptides was in general < 1 ppm after recalibration. Peptide lists were further used by MaxQuant to identify and relatively quantify proteins using the following parameters: Peptide and protein false discovery rates, based on a forward–reverse database, were set to 0.01; minimum peptide length was set to 7; the minimum number peptides for identification and quantitation of proteins was set to two of which one must be unique; minimum ratio count was set to two; and identified proteins were requantified. The “match-between-run” option (2 min) was used.

#### Data analysis

To obtain a list of proteins significantly affected by the treatment with losartan, the data were processed using the freely available Perseus software (Cox *et al*, 2011). Label-free protein abundance values based on extracted ion currents of peptides were log2-transformed and *z*-normalized. The 10 samples analyzed were hierarchically clustered, and protein abundances were k-means-clustered into 10 discrete clusters of similar size. To address the biological implications of the proteins in each cluster, Cellular Compartment, Biological Process and Molecular Function GO terms were retrieved. Significantly enriched GO terms in each cluster were determined using Benjamini and Hochberg-adjusted *P*-values (< 0.05).

The mass spectrometry proteomics data have been deposited to the ProteomeXchange Consortium via the PRIDE partner repository with the dataset identifier PXD002134.

### Statistical analysis

Sample size was computed with log-rank test for a power of 0.8. The GraphPad Prism 5.03 software was used for statistical analysis, and analyses were performed using linear regression, unpaired *t*-test with Welch’s correction, and paired or unpaired two-tailed Student’s *t*-test as indicated. Data were tested for normality by the D’Agostino–Pearson omnibus normality test and similar variance by F test. Beta function and t-value were used to calculate the probability values for the difference between two slopes. For the analysis of proteomics data, significantly enriched GO terms in each cluster were determined using Benjamini and Hochberg-adjusted *P*-values (< 0.05). For all analyses, *P *<* *0.05 was considered statistically significant.

**The paper explained**

**Problem**

Recessive dystrophic epidermolysis bullosa (RDEB) is a severe inherited skin disease manifested with chronic skin fragility and disabling progressive soft tissue fibrosis. No cure exists for this devastating disorder and, therefore, biologically valid therapies are urgently needed. Different gene-, cell-, and protein replacement-based approaches have shown some promise, but massive hurdles must still be mounted and clinical implementation is likely to be in distant future. On a shorter perspective, instead of cure, symptom-relieving treatments are highly prioritized by patients. Indeed, improved knowledge of molecular disease mechanisms in RDEB allows design of alternative symptom-ameliorating therapies, which have the potential to reach clinics relatively fast if constructed by repurposing already approved drugs.

**Results**

Based on the observation that TGF-β activity is highly increased in injured RDEB skin, we used a repurposed drug, losartan, to target TGF-β activity in a preclinical setting. We found that losartan lessens disease burden in RDEB mice by attenuating fibrotic scarring and delaying formation of mutilating forepaw deformities. Unbiased proteomics analyses yielded new information about mechanisms activated in RDEB disease progression and identified processes related to tissue inflammation as significant contributors to disease manifestations. Also these processes were targeted by losartan.

**Impact**

Our study shifts the view of RDEB from a skin disease to a systemic fibrotic disorder with multiorgan involvement, and the results strongly suggest that losartan has the potential to become a much-needed, systemic disease-modulating therapy for RDEB in humans. The study significantly increases the understanding about disease mechanisms and progression by uncovering RDEB-related fibrosis as a consequence of a cascade encompassing tissue damage, TGF-β-mediated inflammation, and matrix remodeling. This knowledge opens up novel targets for RDEB symptom-relieving therapies.

## Supporting Information

**Supplementary Table S2**

**Review Process File**

**Source Data for Figure 5A**

**Source Data for Figure 6C**

**Source Data for Figure 7E**
