# Supplementary material for: Losartan ameliorates dystrophic epidermolysis bullosa and uncovers new disease mechanisms
Source: EMBO Mol Med. 2015 Jul 20;7(9):1211–28. doi: 10.15252/emmm.201505061 (PMC4568953; doi:10.15252/emmm.201505061)
Supplement: Supplementary file 1 [file emmm0007-1211-sd1.pdf]

Supplementary Information

Title: Treatment with losartan ameliorates symptoms and uncovers new disease mechanisms in dystrophic epidermolysis bullosa

Alexander Nyström, Kerstin Thriene, Venugopal Mittapalli, Johannes S Kern, Dimitra Kiritsi, Jörn Dengjel, Leena Bruckner-Tuderman

Table of Contents

Supplementary Table and Figures Legends Page: 2-4

Supplementary Figures S1-S4 Page: 5-8

Supplementary Table S1: Whole skin lysate proteomes of wild type (WT) and collagen VII (C7)-hypomorphic mice

Supplementary Table S2: Enriched GO terms in each cluster.

Supplementary Figure S1 Elevated TGFβ levels in skin wounds and serum of patients with RDEB. A, Granulation tissue in acquired chronic wounds (venous ulcers) and in chronic RDEB wounds stained with antibodies to active TGFβ (red) and phospho-SMAD2/3 (green). TGFβ was highly activated in RDEB wounds. Nuclei counterstained with DAPI (blue). Images acquired with a 40X objective, scale bar = 50 µm. **B**, Western blot of TGFβ in the serum of two patients with genetically confirmed C7-deficient RDEB and of two patients with acquired chronic wounds (venous ulcers). The double band corresponding to the 12 and 25 kDa forms of TGFβ is shown. Equal loading was confirmed by Ponceau S staining. Note the elevated levels of circulating TGFβ in RDEB serum.

Supplementary Figure S2 Losartan reduces human RDEB fibroblast activity. A, *TGFB1* mRNA expression in RDEB fibroblasts grown as in A, analyzed with qPCR and normalized to the housekeeping gene *GAPDH.* The relative expression level of untreated RDEB fibroblasts was set to 1.0, n = 4, values represent mean ± S.E.M, data analyzed by Student's paired *t*-test, ** $P = 0.0088$. **B**, Representative Western blots of protein lysates from normal human fibroblasts (NHF) or RDEB fibroblasts (RDEBF) grown in 10% FCS with or without 10 µM losartan for 48 h. Membranes were probed for TSP1, collagen I α1 chain and β-actin as a loading control. TSP1 expression is regulated by the angiotensin II type 1 receptor. It is

highly expressed in RDEB fibroblasts and is efficiently downregulated by losartan in fibroblasts of both genotypes. The high collagen I expression in RDEB fibroblasts, which is largely dependent on TGFβ, was effectively reduced by losartan. In contrast, losartan treatment did not affect collagen I expression in NHF. **C**, Losartan effectively inhibited RDEB fibroblast contractility. Pictures of free-floating collagen lattices populated with 250,000 RDEB fibroblasts 24 h after start of contraction. Treatment ± 10 μM losartan as indicated. Losartan effectively inhibited contraction of the collagen lattice. Dotted lines indicate the circumference of the gels. The bar graph shows the mean lattice contraction after 24 h from 4 different experiments; values represent mean ± S.E.M, data analyzed by Student's unpaired *t*-test ** $P = 0.0038$.

Supplementary Figure S3. Elevated levels of circulating growth factors and cytokines in RDEB are significantly reduced by losartan treatment. **A**, The bar graphs show Tgfβ1, Tnfα, Il6 content in serum, respectively, of C7-hypomorphic mice treated for 7 weeks with losartan and of age-matched untreated C7-hypomorphic and wild type mice. Tgfβ1 content was measured by dot blots and quantified using Image J. Wild type values were set to 1, and the values of the C7-hypomorphic mice normalized to this. Values represent mean ± S.E.M. Data analyzed by Student's unpaired *t*-test, Tgfβ1, ***P* wild type vs. C7-hypomorph = 0.0002; **P* C7-hypomorph + losartan vs. C7-hypomorph = 0.0435, **P* wild type vs. C7-hypomorph + losartan = 0.30; Il6, ***P* wild type vs. C7-hypomorph = 0.0031; **P* C7-hypomorph + losartan vs. C7-hypomorph = 0.046, **P* wild type vs. C7-hypomorph + losartan = 0.25; Tnfα **P* wild type vs. C7-hypomorph = 0.024; **P* C7-hypomorph + losartan vs. C7-hypomorph = 0.029, **P* wild type vs. C7-hypomorph + losartan = 0.14. **B**, Circulating IL6 is significantly elevated in RDEB patient serum. Serum from unaffected controls and patients with genetically verified C7-deficient RDEB was analyzed by dot blots for IL6 expression.

Blots were quantified as in A, and values normalized to expression in control serum, which was set to 1. Values represent mean ± S.E.M. n = 5, Student's unpaired *t*-test used, $^{*}P$ = 0.0119.

Supplementary Figure S4 Losartan reduces inflammation in C7 deficient skin. Back skin of wild type, C7-hypomorphic and losartan-treated C7-hypomorphic mice stained for Cd11b (green). Nuclei visualized with DAPI (blue), scale bar = 100 μm. Below, the bar graph shows quantification of Cd11b positive cells; values represent mean ± S.E.M, n = 3. Data analyzed by Student's unpaired *t*-test, $^{**}P$ wild type vs. C7-hypomorph = 0.0071; $^{**}P$ C7-hypomorph + losartan vs. C7-hypomorph = 0.0066, *P* wild type vs. C7-hypomorph + losartan = 0.19.

A

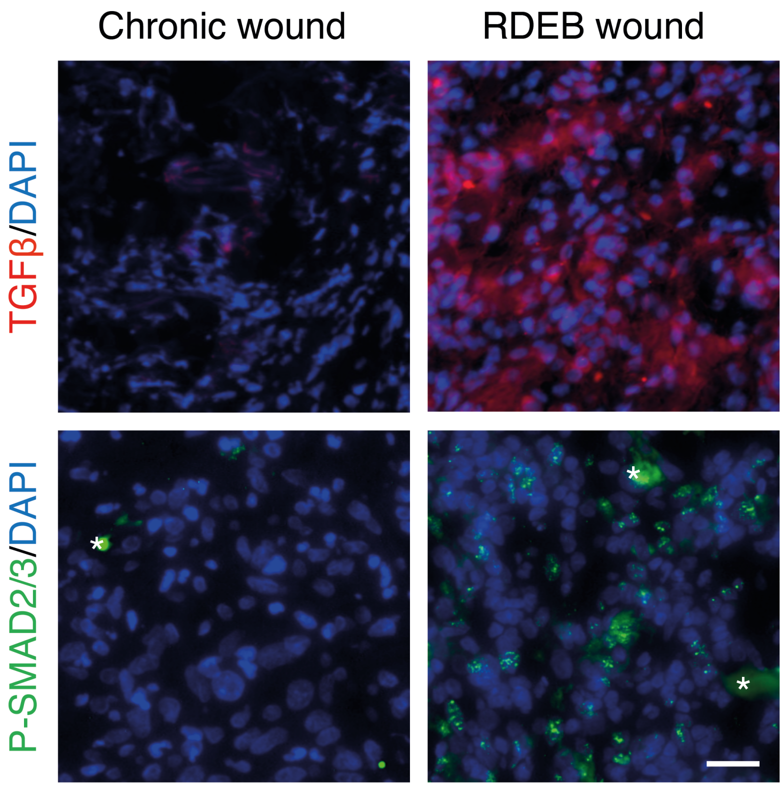

Chronic wound
RDEB wound
TGFβ/DAPI
P-SMAD2/3/DAPI
*
*
*

B

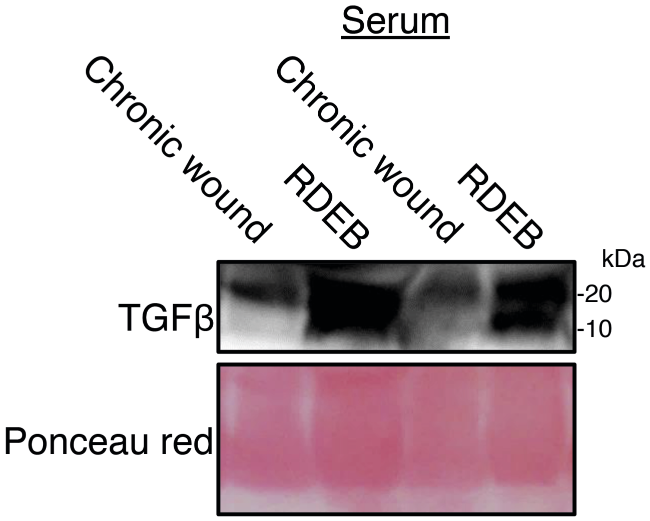

Serum
Chronic wound
RDEB
Chronic wound
RDEB
kDa
TGFβ
-20
-10
Ponceau red

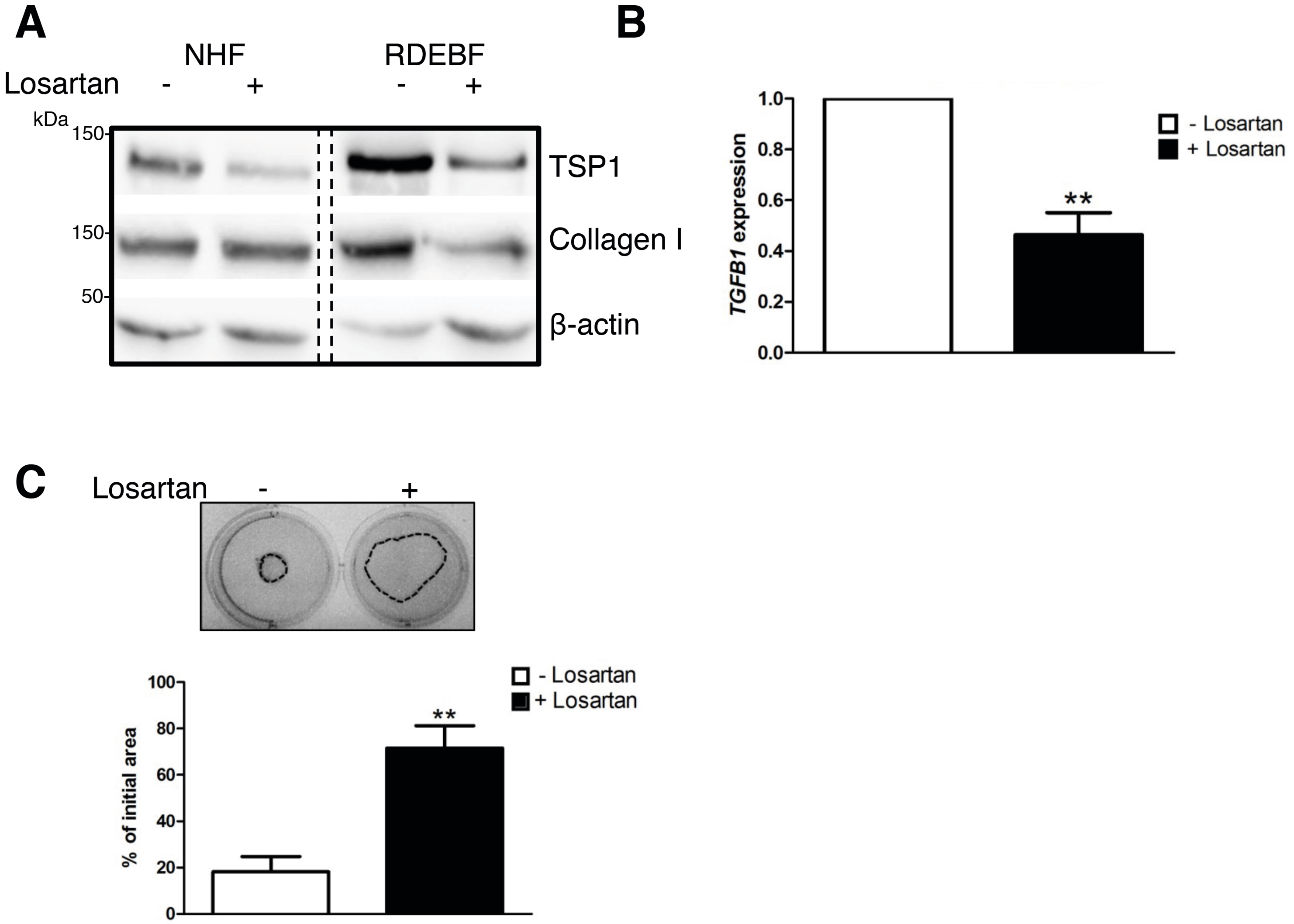
A
NHF
RDEBF
Losartan - + - +
kDa
150
150
50
TSP1
Collagen I
β-actin
B
1.0
0.8
0.6
0.4
0.2
0.0
TGFB1 expression
- Losartan
+ Losartan
**
C
Losartan - +
100
80
60
40
20
0
% of initial area
- Losartan
+ Losartan
**

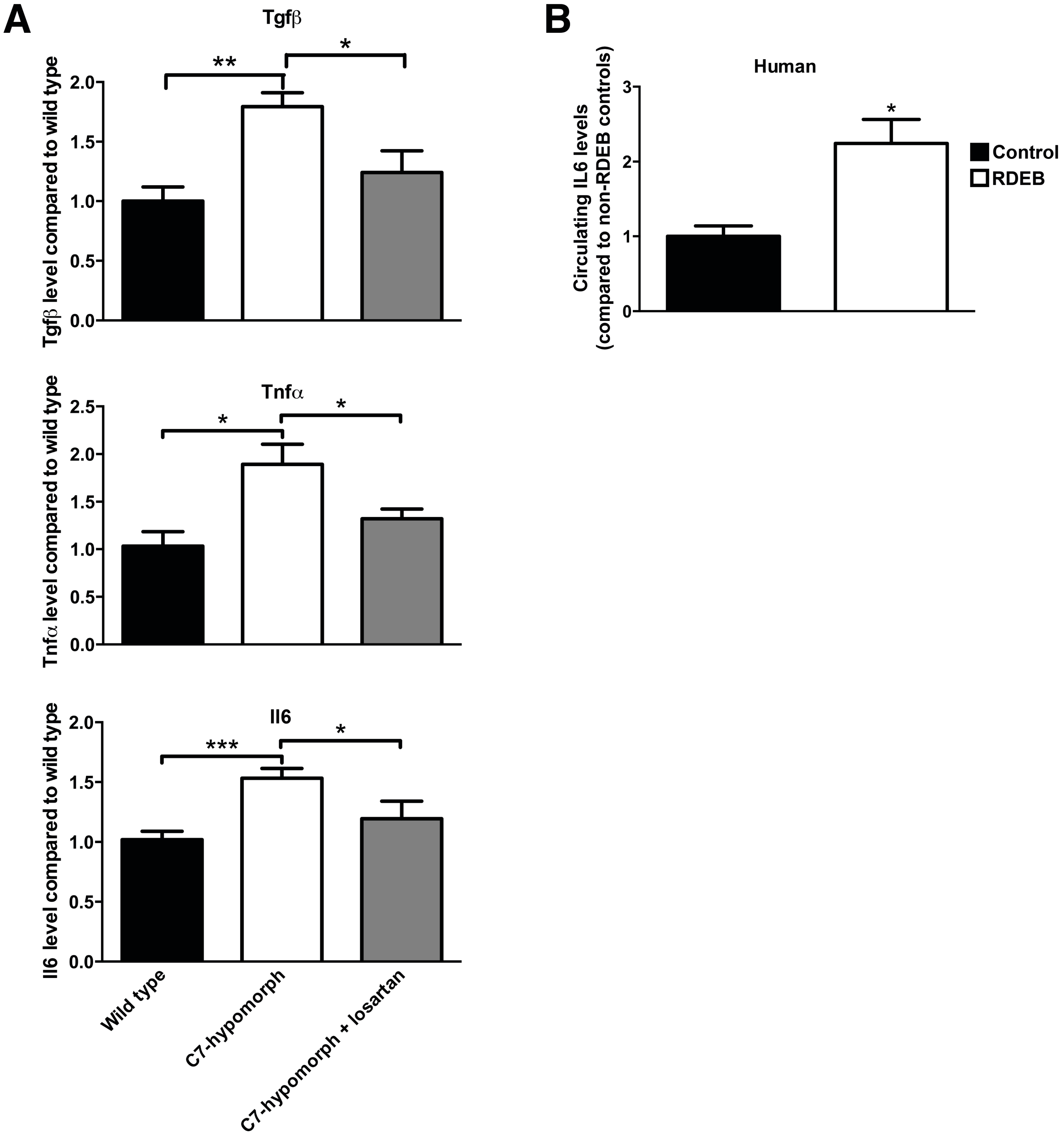
A
Tgfβ
Tgfβ level compared to wild type
2.0
1.5
1.0
0.5
0.0
**
*
Tnfα
Tnfα level compared to wild type
2.5
2.0
1.5
1.0
0.5
0.0
*
*
Il6
Il6 level compared to wild type
2.0
1.5
1.0
0.5
0.0
***
*
Wild type
C7-hypomorph
C7-hypomorph + losartan
B
Human
Circulating IL6 levels
(compared to non-RDEB controls)
3
2
1
0
*
Control
RDEB

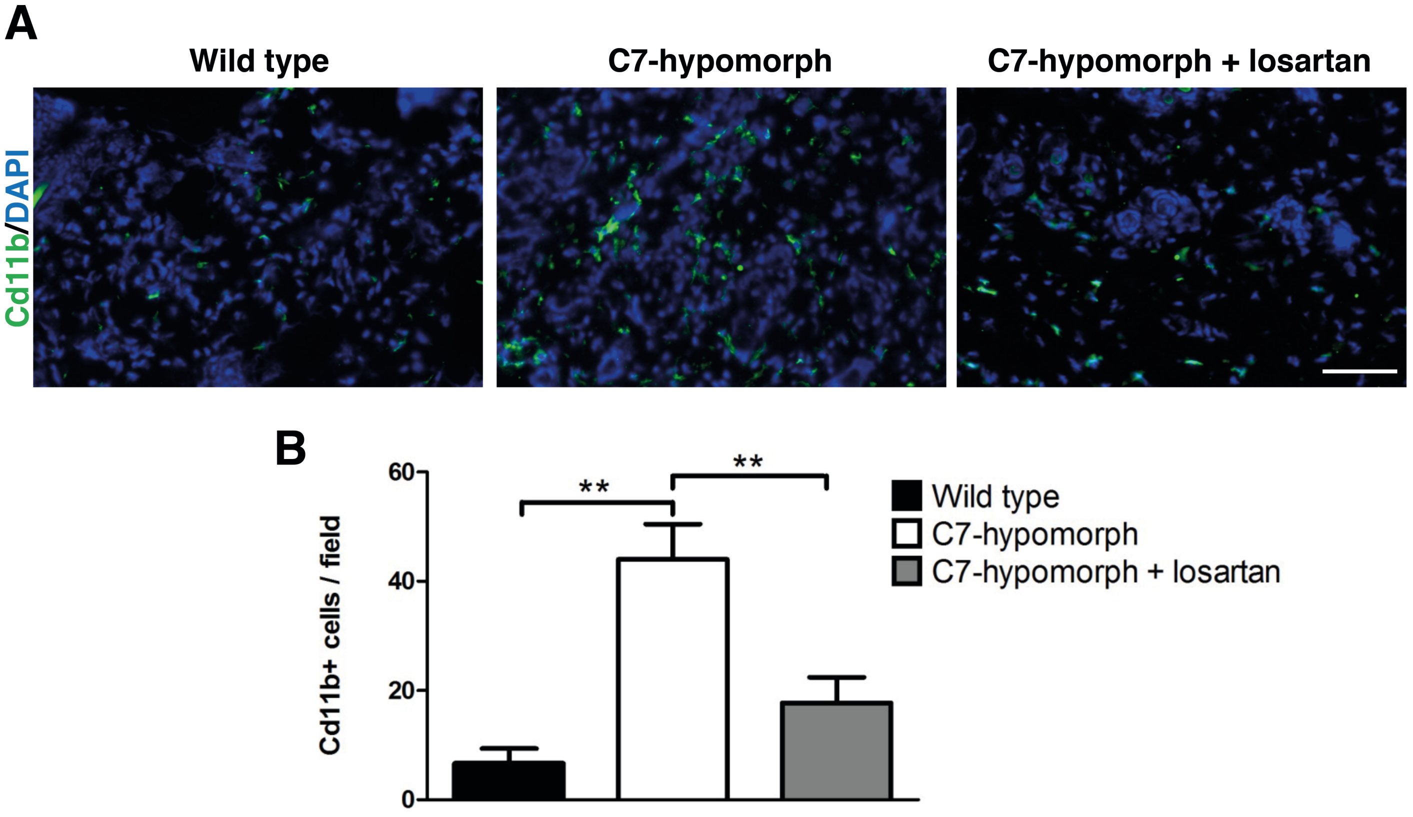
A
Wild type
C7-hypomorph
C7-hypomorph + losartan
Cd11b/DAPI
B
Cd11b+ cells / field
60
40
20
0
**
**
Wild type
C7-hypomorph
C7-hypomorph + losartan
