# Supplementary material for: Losartan ameliorates dystrophic epidermolysis bullosa and uncovers new disease mechanisms
Source: EMBO Mol Med. 2015 Jul 20;7(9):1211–28. doi: 10.15252/emmm.201505061 (PMC4568953; doi:10.15252/emmm.201505061)
Supplement: Supplementary file 4 [file emmm0007-1211-sd4.pdf]

EMBO Molecular Medicine

Losartan ameliorates dystrophic epidermolysis bullosa and uncovers new disease mechanisms

Alexander Nyström, Kerstin Thriene, Venugopal Mittapalli, Johannes S Kern, Dimitra Kiritsi, Jörn Dengjel, Leena Bruckner-Tuderman

Corresponding author: Leena Bruckner-Tuderman, University of Freiburg

Review timeline:

Submission date:	23 January 2015
Editorial Decision:	23 February 2015
Revision received:	01 May 2015
Editorial Decision:	03 June 2015
Accepted:	18 June 2015

Transaction Report:

(Note: With the exception of the correction of typographical or spelling errors that could be a source of ambiguity, letters and reports are not edited. The original formatting of letters and referee reports may not be reflected in this compilation.)

Editor: Roberto Buccione

1st Editorial Decision 23 February 2015

Thank you for the submission of your manuscript to EMBO Molecular Medicine. We have now received comments from the Reviewers whom we asked to evaluate your manuscript.

You will see that the Reviewers are supportive of your work, although they do express a number of partially overlapping concerns that prevent us from considering publication at this time. I will not dwell into much detail, as the evaluations are self-explanatory. I would like, however, to highlight a few main points.

Reviewer 1 is globally positive and suggests, among other things, that the proteomic screen for losartan-affected proteins should be better presented and capitalised on.

Reviewer 2 is also positive and offers many valuable suggestions, which require your action. For instance, s/he finds that the data presented in a number of figures should be improved by providing western blot quantifications and that it should be also investigated whether regulation is transcriptional or post-transcriptional. Reveiwer 2 also lists a number of other items for your action.

Reviewer 3 while generally positive, is more reserved. His/her concerns are that evidence that losartan directly affects inflammatory responses is lacking and suggests a number of approaches through which this could be better ascertained, including mechanistic follow-up on a number of candidate proteins arising from your analysis. I agree that evidence that Losartan indeed improves the symptoms via reduction of Tgf-beta levels is of a correlative nature and that this requires further work. Reviewer 3 also notes, as does Reviewer 1, the lack of convincing statistical analysis on the proteomics data and, similarly to Reviewer 2, would like to see more western blotting-based analysis.

In conclusion, while publication of the paper cannot be considered at this stage, we would be pleased to consider a suitably revised submission, provided that the Reviewers' concerns are addressed as outlined above, including with additional experimental data where indicated.

Please note that it is EMBO Molecular Medicine policy to allow a single round of revision only and that, therefore, acceptance or rejection of the manuscript will depend on the completeness of your responses included in the next, final version of the manuscript.

As you know, EMBO Molecular Medicine has a "scooping protection" policy, whereby similar findings that are published by others during review or revision are not a criterion for rejection. Although I clearly do not foresee such a delay in this case, I do ask you to get in touch with us after three months if you have not completed your revision, to update us on the status. Please also contact us as soon as possible if similar work is published elsewhere.

Please note EMBO Molecular Medicine now requires a complete author checklist (http://embomolmed.embopress.org/authorguide#editorial3) to be submitted with all revised manuscripts. Provision of the author checklist is mandatory at revision stage; The checklist is designed to enhance and standardize reporting of key information in research papers and to support reanalysis and repetition of experiments by the community. The list covers key information for figure panels and captions and focuses on statistics, the reporting of reagents, animal models and human subject-derived data, as well as guidance to optimise data accessibility. Please make sure that the relevant information is also included in the main manuscript text.

I look forward to receiving your revised manuscript as soon as possible.

***** Reviewer's comments *****

Referee #1 (Comments on Novelty/Model System):

1. Generally very good quality, but I thought that the proteomic data are analysed rather superficially. Rather than just showing cluster analysis it would be good to see some pathway/gene ontology analysis - I think this would make the data more accessible for the general reader, as well as being of more interest to the specialist.

2. The study is novel and interesting - it repurposes a known drug and details some exciting aspects of RDEB pathology.

3. The study identifies a completely new potential therapeutic approach to treating RDEB.

4. The study uses a range of complementary models - 2d and 3d cell culture, clinical samples, mouse models.

Referee #1 (Remarks):

The authors present an interesting and well written study, detailing a novel therapeutic approach to the treatment of RDEB - using an angiotensin II type 1 receptor antagonist. They provide an excellent introduction - with good background and well-explained rationale for the study.
Losartan has remarkable effects in their mouse model of paw deformity. One minor query was the dose - did they determine this from the average water intake of RDEB mice or from a normal control mouse? Many mouse models with barrier defects show dramatically increased water intake as a result - and this might mean the mice are getting a larger dose than predicted. Would be good to clarify this.
Moving on from the phenotypic change, they show convincing molecular data implicating TGFbeta driven fibrosis in RDEB fibrosis, and build on other studies to show that Losartan acts via reducing TGFbeta signalling.
In addition to a range of convincing stainings/expression analysis for known markers of fibrosis and the TGFbeta pathway, they perform proteomic screening for losartan regulated proteins. This

unveils some interesting proteins, but having performed an unbiased screen they then seem to cherry pick genes rather than showing any measure of the level of significance of each protein, as they might have done. Here I feel they miss a trick - 1. not showing detailed pathway analysis of the data that would be interesting for general readership as well as specialists, and 2. missing the chance to describe really novel proteins which may be involved.
That said, they provide evidence for a clear role for inflammation - though I don't see that as being particularly novel or surprising.
Very minor point is repeated mis-spelling of hypomorphic on page 9.
The figures are excellent - it would be good to have more interpretation of proteomic data, rather than the graphs in 4b-e, which have very odd y-axe values - they need clear breaks in the axes if they are going to represent the data like this.

Referee #2 (Comments on Novelty/Model System):

The authors describe a novel treatment method for RDEB using losartan to reduce the symptoms of fibrosis and fused digits using a mouse model of RDEB. The results presented are promising, but the manuscript could be improved as outlined below.

Referee #2 (Remarks):

This is a preclinical trial that evaluates the treatment effect of losartan, a repurposed drug already shown effective in a mouse model of Marfan syndrome. The authors focus on TGF -mediated ECM remodeling that occurs in RDEB patients and causes fusion of fingers or squamous cell carcinomas. Losartan was administered to collagen VII hypomorphic mice and alleviated symptoms resulting from ECM remodeling through TGF inhibition.

The data presented suggest an alternative treatment approach to alleviate some symptoms in RDEB patients.

The manuscript could be improved as follows:

1. Page 5 line 19 "Losartan reduces RDEB fibrosis", in addition to the gross findings that look promising, the authors should include histological analysis (H & E staining) after treatment to further document a reduction in fibrosis.

2. Page 11, line 10~ "TNF gene expression ~" and Figure 5-B and C, mRNA seems to have been extracted from the tissue, but this reviewer could not find the extraction method anywhere.

3. Figure 2-C,D, 3-D, S1-A, and S5, the expression levels of proteins that are associated with the TGF pathway or fibrosis/wound healing were analyzed by immunofluorescent staining. However, the images shown are not good enough to validate the quantification. The effect of losartan on modulating the expression levels of these proteins should be quantified Western blotting. To document whether this is occurring post-transcriptionally or at the transcriptional level, the authors should include an analysis of the expression levels of mRNA encoding these proteins.

4. Figure S3, this graph shows a reduction in the serum levels of TGF in losartan treated mice, and Figure S6 shows an increase in the serum levels of IL6 in RDEB mice, were the serum levels of IL6 and TNF reduced following losartan treatment?

5. The data presented needs to be better organized to improve the readability. Several of the supplemental figures can be combined and some are redundant.

© EMBO

Referee #3 (Remarks):

This paper describes preclinical proof-of-concept studies on the use of the angiotensin II type I receptor antagonist losartan for the treatment of a mouse model for recessive dystrophic epidermolysis bullosa (RDEB). In addition, the authors conduct proteomic studies to analyze the pathways that are affected by this treatment. Overall, the work provides strong evidence that the disease progression of RDEB is slowed-down by losartan. Although the proteomic analysis indeed provides potential new insights into the mechanisms involved in this activity of losartan, I think that more work will be needed to indeed show that those pathways are targets of losartan.

Specifically, there is no evidence that losartan directly affects inflammatory responses nor whether this down-regulation is actually a result of its inhibitory function on the TGF-beta pathway. To get more insights into the mechanisms, one could for example look at temporal changes on those pathways during the progression of RDEB. Such experiments could be done using proteomics or Western blot analysis of some selected candidate proteins. Moreover, short-term-treatment with losartan or a more selective TGF-beta antagonist may further reveal the causal relationship between inflammation and TGF-beta signaling.

Additional remarks:
- The presentation of the data in Figure 1C is misleading because the authors use a line generated by linear regression from 3 data points to suggest a linear progression of the disease. The linear progression in the shortening of the digits is in fact not shown in the paper and thus such conclusion should be avoided. The linear regression implies that this has been measured. I therefore propose to present the data as a histogram instead. The interpretation with the linear progression could still be discussed in the text.

- The differential regulation of proteins shown in Figure 4 should also be confirmed using Western Blot analysis and/or immunohistochemical stainings.

- It was not clear to me as to how the proteomic data were statistically analyzed. Are the significance values shown based on the difference between individual mice, between the different skin sections or between skin sections including the technical replicates? This should be explicitly stated.
In addition it was not clear whether the 5,038 proteins identified and the 4,028 proteins quantified were observed in all of the samples or they appeared at least in one of the samples. To avoid this difficulty, more details should be given in the material and method section.

1st Revision - authors' response 01 May 2015

Below is a summary of changes to the manuscript as suggested by the Reviewers,

1. Figure 1C is now presented as a bar graph.

2. We have added histological analysis of fibrosis (**new Fig 2**).

3. Extensive Western blot and qPCR analyses of forepaws have been performed (**new Fig 5**).

4. The presentation of the proteomics data has been significantly revised; pathway analysis has been performed and the differential regulation of proteins shown confirmed by Western blotting (**new Fig 6**).

6. We have performed extensive analysis of links between disease progression and inflammation in RDEB and confirmed that inflammation is a direct major target of losartan (**new Fig 7E-H**).

7. All data generated by proteomics analysis are presented (**new Supplementary Table S1**).

8. Serum concentration of Tnfα and Il6 has been measured (**new Supplementary Fig S3A**).

9. The Supplementary Material has been substantially reorganized: the old Supplementary Figs S3 and S6 were merged into **new Supplementary Fig S3,** and the old Supplementary Figs S4 and S5 were omitted.

Point-by-point reply.

******* Reviewer's comments *******

Referee #1 (Comments on Novelty/Model System):

1. Generally very good quality, but I thought that the proteomic data are analysed rather superficially. Rather than just showing cluster analysis it would be good to see some pathway/gene ontology analysis - I think this would make the data more accessible for the general reader, as well as being of more interest to the specialist.

We added more information regarding the proteomics data processing and quality into the text. Importantly we added a new Supplementary Table 1 giving details about all proteins consistently quantified in all samples and a new Supplementary Table 2 highlighting all enriched GO terms in all clusters.

To better present our line of analysis and argumentation, as suggested by the reviewer, we added a new protein interaction network as Figure 6B. This Figure highlights proteins involved in the deregulation of immune modulatory/inflammatory process in RDEB. A subset of these proteins was analyzed by western blot, as shown in Figure 6C.

2. The study is novel and interesting - it repurposes a known drug and details some exciting aspects of RDEB pathology.

3. The study identifies a completely new potential therapeutic approach to treating RDEB.

4. The study uses a range of complementary models - 2d and 3d cell culture, clinical samples, mouse models.

Referee #1 (Remarks):

The authors present an interesting and well written study, detailing a novel therapeutic approach to the treatment of RDEB - using an angiotensin II type 1 receptor antagonist. They provide an excellent introduction - with good background and well-explained rationale for the study.

We thank the reviewer for the interest in our work and the positive remarks.

1. Losartan has remarkable effects in their mouse model of paw deformity. One minor query was the dose - did they determine this from the average water intake of RDEB mice or from a normal control mouse? Many mouse models with barrier defects show dramatically increased water intake as a result - and this might mean the mice are getting a larger dose than predicted. Would be good to clarify this.

This is an insightful comment. Adult RDEB mice, which were used for these studies, do not display a widespread severe barrier defect, since the fur protects the skin from damage. Blistering and scarring occur preferentially and most extensively at unprotected, friction-exposed sites such as paws and face. The RDEB mice were kept together with unaffected littermates for social reasons. To estimate the water consumption we weighed the water bottles before attaching them to cages and after 24 hours, reference bottles attached to empty cages served as control for leakage. The weight loss was converted to water volume and to estimate the average consumption per mouse divided by the number of mice in the cage. Thus, value represents the average consumption per mouse regardless of genotype and not accounting for differences in consumption due to genotype. In most cages 1 C7-hypomorphic and 4 wild type mice or 2 C7-hypomorphic and 3 wild type mice and the water consumption was similar in these cages. We have now included a more detailed description of the dose determination in the **Materials and Methods** section. Page 18 lines 7-14.

2. Moving on from the phenotypic change, they show convincing molecular data implicating TGFbeta driven fibrosis in RDEB fibrosis, and build on other studies to show that Losartan acts via reducing TGFbeta signalling. In addition to a range of convincing stainings/expression analysis for known markers of fibrosis and the TGFbeta pathway, they perform proteomic

screening for losartan regulated proteins. This unveils some interesting proteins, but having performed an unbiased screen they then seem to cherry pick genes rather than showing any measure of the level of significance of each protein, as they might have done. Here I feel they miss a trick - 1. not showing detailed pathway analysis of the data that would be interesting for general readership as well as specialists, and 2. missing the chance to describe really novel proteins which may be involved.

We had originally chosen to show principal proteins found in cluster analysis, but agree with the reviewer that more could be done with the data. Now, the selected proteins found by cluster analysis are still shown but we have also majorly revised this section (**new Figure 6** (old Figure 4)). We have performed additional pathway analysis and also chosen to present the complete lists of all hits as Supplementary Table 1. Deregulated proteins from cluster 5 were analyzed on interactions by STRING DB, and an interaction network was added as **Figure 6B**. Enriched GO terms from all clusters are shown in Supplementary Table 2. The new data are accordingly presented in **the Results** section, page 11, lines 1-25 and page 12, lines 1-3.

3.That said, they provide evidence for a clear role for inflammation - though I don't see that as being particularly novel or surprising.

It is true that in many fibrotic disorders inflammation is involved in the pathology. However, recessive dystrophic epidermolysis bullosa (RDEB), as consequence of its immediately visible primary skin fragility, has been widely viewed as a skin blistering disorder rather than a fibrotic disorder. Therefore, secondary disease mechanisms have generally not been considered. Our description of the role of inflammation for RDEB pathology might not be surprising from the general view of a fibrotic disorders, but it is novel and important for RDEB. This description will have significant impact on how RDEB diseases mechanisms will be viewed in the future and will have great impact on therapy development.

4. Very minor point is repeated mis-spelling of hypomorphic on page 9.

Thank you for noticing the mistake. This has now been corrected.

5. The figures are excellent - it would be good to have more interpretation of proteomic data, rather than the graphs in 4b-e, which have very odd y-axe values - they need clear breaks in the axes if they are going to represent the data like this.

As stated above, the proteomics part has been completely revised and we have included increased interpretation of the proteomics data. As proteomics analysis measures protein abundance, in contrast to classical biochemical analysis that rather measures concentration, we presented the data in the old graphs in 4B-E with constant interval between the ticks on the y-axes in a way to illustrate difference in abundance of the selected proteins and their changes in the groups. However, we agree with the reviewer that this unconventional way of presenting data can be confusing and have now replotted the graphs (new **Figure 6C**) with constant length of the y-axes.

Referee #2 (Comments on Novelty/Model System):

The authors describe a novel treatment method for RDEB using losartan to reduce the symptoms of fibrosis and fused digits using a mouse model of RDEB. The results presented are promising, but the manuscript could be improved as outlined below.

Referee #2 (Remarks):

This is a preclinical trial that evaluates the treatment effect of losartan, a repurposed drug already shown effective in a mouse model of Marfan syndrome. The authors focus on TGFβ-mediated ECM remodeling that occurs in RDEB patients and causes fusion of fingers or squamous cell carcinomas. Losartan was administered to collagen VII hypomorphic mice and alleviated symptoms resulting from ECM remodeling through TGFβ inhibition.

The data presented suggest an alternative treatment approach to alleviate some symptoms in RDEB patients.

The manuscript could be improved as follows:

1. Page 5 line 19 "Losartan reduces RDEB fibrosis", in addition to the gross findings that look promising, the authors should include histological analysis (H & E staining) after treatment to further document a reduction in fibrosis.

This is a very good suggestion. We have performed detailed histological analysis of forepaws of control and losartan-treated RDEB mice. H&E and Elastica van Gieson stainings are shown in the new ***Figure 2****. These morphological analyses show that losartan clearly reduces signs of fibrosis also on the histological level. It effectively limited presence of inflammatory infiltrates, deposition of dense fibrotic material and expansion of the dermis. The findings are detailed in the* ***Results*** *section, page 7 lines 16-23.*

2. Page 11, line 10~ "TNFα gene expression ~" and Figure 5-B and C, mRNA seems to have been extracted from the tissue, but this reviewer could not find the extraction method anywhere.

We apologize for neglecting to include the important information on how mRNA was isolated. We have now added to the **Materials and Methods** section a detailed description of mRNA extraction from paws. Page 20, lines 3-6.

3. Figure 2-C,D, 3-D, S1-A, and S5, the expression levels of proteins that are associated with the TGFβ pathway or fibrosis/wound healing were analyzed by immunofluorescent staining. However, the images shown are not good enough to validate the quantification. The effect of losartan on modulating the expression levels of these proteins should be quantified Western blotting. To document whether this is occurring post-transcriptionally or at the transcriptional level, the authors should include an analysis of the expression levels of mRNA encoding these proteins.

We agree that Western blotting may represent more accurate means of quantification of proteins involved in profibrotic processes. Therefore, we performed additional Western blot analyses of all proteins shown in old Figures 2 and 3, except TGFβ (TGFβ was not analyzed due to concerns regarding accuracy and false positive/negative results, since its active form is a small easily diffusible protein, and the protein extraction from paws involved a dissection step). The Western blot analyses are now presented together with densitometric quantifications in new **Figure 5A** and **B** and in the **Results** section page 9, lines 12-15. Importantly, these extended analyses corroborated the initial immunofluorescence findings. Further, by the advice of the reviewer we determined expression by qPCR; we had previously shown regulation of *TGFB1* by losartan (Supplementary Figure S2B). The new qPCR analysis suggested that in general the effect on regulation seems to occur on the transcriptional level which is in line with previous reports on losartan's mode of action in other fibrotic conditions (Gay-Jordi et al, 2013). We now present data from these analyses in new **Figure 5C** and the **Results** section page 9, lines 15-21.

Supplementary Figures S1A and S5 showed patient material. The biopsies were collected for clinical diagnostics using histopathology and no special caution was taken for RNA analyses. Thus, the quality of the tissue preservation for use in other assays cannot be guaranteed. Indeed, lack of RNAase inhibitors and handling at room temperature had facilitated RNA degradation, and the RNA isolated from sections was not of sufficient quality for reliable qPCR analysis. It would be unethical to subject the patients to new biopsies to collect material for Western blotting and qPCR, especially considering the inherently perturbed wound healing and intensive pain connected to such procedures in patients with chronic wounds or RDEB. Importantly, our new additional analyses (new **Figure 5** and new **Figure 6**) on mouse tissue nicely supported the data generated by patient materials. - Additionally, by the advice of the reviewer we have significantly restructured the supplementary data presentation which has led to the omission of former Supplementary Figure S5.

4. Figure S3, this graph shows a reduction in the serum levels of TGFβ in losartan treated mice, and Figure S6 shows an increase in the serum levels of IL6 in RDEB mice, were the serum levels of IL6 and TNFα reduced following losartan treatment?

Given that RDEB patients show elevated levels of circulating IL6 (old Supplementary Figure S6 and new Supplementary Figure S3) it is fair to ask whether this is also seen in RDEB mice. We have now measured Il6 and Tnfα in serum in wild type, RDEB mice and RDEB mice receiving losartan. Both Il6 and TNFα were elevated in RDEB mice. Notably, losartan treatment reduced these levels, supporting our observation that inflammation is a major target of losartan. These data have been included in new **Supplementary Figure S3** and also included in the **Results** section page 11 lines 17-19 and page 12 lines 18-19.

5. The data presented needs to be better organized to improve the readability. Several of the supplemental figures can be combined and some are redundant.

By the suggestion of the reviewer we have rearranged the Supplementary Material. Former Supplementary Figures S3 and S6 have been merged to a new **Supplementary Figure S3** and Supplementary Figure S4 omitted due to the fact that the data corresponding to this figure are now included in the new Supplementary Table 1. Also, old Supplementary Figure S5 has been deleted in order to limit redundancies and to increase readability of the manuscript.

Referee #3 (Remarks):

This paper describes preclinical proof-of-concept studies on the use of the angiotensin II type I receptor antagonist losartan for the treatment of a mouse model for recessive dystrophic epidermolysis bullosa (RDEB). In addition, the authors conduct proteomic studies to analyze the pathways that are affected by this treatment. Overall, the work provides strong evidence that the disease progression of RDEB is slowed-down by losartan.

1. Although the proteomic analysis indeed provides potential new insights into the mechanisms involved in this activity of losartan, I think that more work will be needed to indeed show that those pathways are targets of losartan. Specifically, there is no evidence that losartan directly affects inflammatory responses nor whether this down-regulation is actually a result of its inhibitory function on the TGF-beta pathway. To get more insights into the mechanisms, one could for example look at temporal changes on those pathways during the progression of RDEB. Such experiments could be done using proteomics or Western blot analysis of some selected candidate proteins. Moreover, short-term-treatment with losartan or a more selective TGF-beta antagonist may further reveal the causal relationship between inflammation and TGF-beta signaling.

We thank the reviewer for the perceptive comment and suggestions.

The new pathway analysis of the proteomics data presented as new **Figure 6B** provides further evidence that inflammation is connected to disease progression in RDEB and that it is a major target of losartan. In addition, we used Western blot analysis (new **Figure 7E**) to validate the deregulation of one of the identified proteins (C1q) in RDEB and thus provided further evidence that tissue inflammation is a disease modifier in RDEB. Increased levels of C1q and IgG, both indicative of tissue inflammation, corresponded to a more severe fibrosis and mitten deformities (new **Figure 7E** and **F**). As expected, TGFβ signaling correlated with disease severity and extent of fibrosis (new **Figure 7F**). These observations are now shown in new **Figures 6C**, **Figure 7E** and **F**, and presented in the **Results** section page 13 lines 1-14.

To further corroborate these observations and clarify the mode of action of losartan we took the reviewer's advice and performed short-term treatments with losartan. The effects of these treatments were analyzed with immunofluorescence staining in order to address the processes specifically in the dermis. Analysis by Western blotting would only provide information on the average TGFβ and inflammation activity in the whole tissue and not differentiate between the skin layers and areas. As shown in the new **Figure 5**, we confirmed by Western blotting that losartan treatment effectively attenuates TGFβ signaling. In the new **Figure 7F** and **G** we show that a short-term (4-day) treatment with losartan effectively lowered Smad2/3 phosphorylation in injured dermis, as compared to untreated dermis in paws with similar degree of injury and fibrosis. Importantly, there was a concomitant reduction in inflammation as determined by the number of Cd11b positive cells (new **Figure 7F** and **H**). - These additional experiments established more firmly the close relationship between losartan treatment, silencing of TGFβ signaling and reduction in inflammation, since the time between start and end of treatment was too short to affect visible fibrosis. The results suggest

that losartan rapidly silences elevated TGFβ signaling in the injured dermis, which, in turn, alleviates injury-driven and TGFβ-promoted inflammation. Importantly, our work collectively supports the hypothesis that lowering the inflammatory burden slows progression and ameliorates disease in RDEB. These additional results have been included in the **new Figures 7F, G** and **H**, and described in the **Results** section page 13 lines 16-24.

Please note: We opted not to add other specific TGFβ antagonists as this would diffuse the study and could not be directly relevant in the present context. The fact that losartan strongly opposed TGFβ signaling was already shown (new **Figures 3** and **5**, and Supplementary Figure S2). In addition, the effect of losartan on TGFβ signaling and the connection between the renin-angiotensin system and TGFβ signaling have been well documented in studies from independent research groups (e.g. Acuna et al, 2014; Habashi et al, 2006; Lim et al, 2001; Matt et al, 2009)

Additional remarks:

2. - The presentation of the data in Figure 1C is misleading because the authors use a line generated by linear regression from 3 data points to suggest a linear progression of the disease. The linear progression in the shortening of the digits is in fact not shown in the paper and thus such conclusion should be avoided. The linear regression implies that this has been measured. I therefore propose to present the data as a histogram instead. The interpretation with the linear progression could still be discussed in the text.

To generate the lines in Figure 1C we used 4 data points 0, 2, 4, and 7 weeks. Paws of the same mice were measured at the different time points; extended recording was unfortunately not possible due to the deteriorating health and severity of disease progression in the control group. When analyzed by linear regression the R^2 for untreated was 0.97 and for treated 0.99 clearly suggesting that the progression on average in the groups could be described as linear. However, as there is some individual deviation from the linear progression, we agree that a line graph may not be the best presentation of the data. As we compare two conditions at four different time points we think that presentation in the form of a histogram would be less accessible than a bar graph. Therefore, we now present our data in the form of the latter (revised **Figure 1C)** and have updated the corresponding part in the **Results** section accordingly, page 7 lines 5-10.

3. - The differential regulation of proteins shown in Figure 4 should also be confirmed using Western Blot analysis and/or immunohistochemical stainings.

We thank the reviewer for the good suggestion. Our previous work has shown that there is generally a very good correlation between protein abundance determined by mass spectrometry-based proteomics and protein amount determined by Western blotting (Kuttner et al., 2013). As the reviewer suggested, to strengthen the present results using different techniques and additional biological replicates, we performed Western blot analysis of selected proteins in skin protein extracts from mice not used for the proteomics analysis. The results verified the proteomics data and the regulation of the selected proteins. These data have been included in the revised new **Figure 6C** and presented in the **Results** section page 11, lines 8-25.

4. - It was not clear to me as to how the proteomic data were statistically analyzed. Are the significance values shown based on the difference between individual mice, between the different skin sections or between skin sections including the technical replicates? This should be explicitly stated.

All reported proteins and peptides were filtered on a FDR<1%. For the generation of the heat map, protein abundance values were log2 transformed and z normalized generating a normally distributed dataset. Thus, parametric test could be used to determine significantly changed proteins. The selected proteins in **Figure 6C** were analyzed by t-test ($p<0.05$). The significance values are shown based on different mice. We added a respective statement to the figure **legend**.

5. In addition it was not clear whether the 5,038 proteins identified and the 4,028 proteins quantified were observed in all of the samples or they appeared at least in one of the samples. To avoid this difficulty, more details should be given in the material and method section.

We apologize, this was indeed misleading. In total we identified 5,308 proteins of which we could quantify 4,028 in minimally one sample. The consistently quantified dataset comprised 2,242 proteins. We added a corresponding statement into the Materials and Methods section, as requested.

References

Acuna MJ, Pessina P, Olguin H, Cabrera D, Vio CP, Bader M, Munoz-Canoves P, Santos RA, Cabello-Verrugio C, Brandan E (2014) Restoration of muscle strength in dystrophic muscle by angiotensin-1-7 through inhibition of TGF-beta signalling. Hum Mol Genet 23**:** 1237-1249

Gay-Jordi G, Guash E, Benito B, Brugada J, Nattel S, Mont L, Serrano-Mollar A (2013) Losartan prevents heart fibrosis induced by long-term intensive exercise in an animal model. PLoS One 8**:** e55427

Habashi JP, Judge DP, Holm TM, Cohn RD, Loeys BL, Cooper TK, Myers L, Klein EC, Liu G, Calvi C et al (2006) Losartan, an AT1 antagonist, prevents aortic aneurysm in a mouse model of Marfan syndrome. Science 312**:** 117-121

Lim DS, Lutucuta S, Bachireddy P, Youker K, Evans A, Entman M, Roberts R, Marian AJ (2001) Angiotensin II blockade reverses myocardial fibrosis in a transgenic mouse model of human hypertrophic cardiomyopathy. Circulation 103**:** 789-791

Matt P, Schoenhoff F, Habashi J, Holm T, Van Erp C, Loch D, Carlson OD, Griswold BF, Fu Q, De Backer J et al (2009) Circulating transforming growth factor-beta in Marfan syndrome. Circulation 120**:** 526-532

2nd Editorial Decision 03 June 2015

Thank you for the submission of your revised manuscript to EMBO Molecular Medicine. We have now received the enclosed reports from the referees that were asked to re-assess it. As you will see the reviewers are now globally supportive and I am pleased to inform you that we will be able to accept your manuscript pending the following final amendments:

1) Could you please state the gender of the mice used in the experiments? Please also mention their age in the Materials and Methods section (the latter in addition to the figure legends, which you have already done)? The red lettering in the manuscript is no longer needed and therefore it can be removed.

2) We are now encouraging the publication of source data, particularly for electrophoretic gels and blots, with the aim of making primary data more accessible and transparent to the reader. Would you be willing to provide a PDF file per figure that contains the original, uncropped and unprocessed scans of all or at least the key gels used in the manuscript? The PDF files should be labeled with the appropriate figure/panel number, and should have molecular weight markers; further annotation may be useful but is not essential. The PDF files will be published online with the article as supplementary "Source Data" files. If you have any questions regarding this just contact me.

3) Every published paper now includes a 'Synopsis' to further enhance discoverability. Synopses are displayed on the journal webpage and are freely accessible to all readers. They include a short sandfirst as well as 2-5 one sentence bullet points that summarise the paper. Please provide the synopsis including the short list of bullet points that summarise the key NEW findings. The bullet points should be designed to be complementary to the abstract - i.e. not repeat the same text. We encourage inclusion of key acronyms and quantitative information. Please use the passive voice.

Please attach this information in a separate file or send them by email, we will incorporate it accordingly. You are also welcome to suggest a striking image or visual abstract to illustrate your article. If you do please provide a jpeg file 550 px-wide x 400-px high.

Please submit your revised manuscript within two weeks.

***** Reviewer's comments *****

Referee #1 (Comments on Novelty/Model System):

The authors have addressed all my original concerns, as well as several other excellent points, and present a significantly improved manuscript.

Referee #1 (Remarks):

All comments addressed

Referee #2 (Remarks):

The revised manuscript is greatly improved and suitable for publication.

Referee #3 (Comments on Novelty/Model System):

The authors have addressed all of my comments adequately. Overall, the manuscript is greatly improved and thus suitable for publication.

Referee #3 (Remarks):

I have carefully read the revised version and found that all of my suggestions have adequately been addressed by the authors. The work is clearly improved and thus I do recommend acceptance.

© EMBO
