# Supplementary material for: Losartan ameliorates dystrophic epidermolysis bullosa and uncovers new disease mechanisms
Source: EMBO Mol Med. 2015 Jul 20;7(9):1211–28. doi: 10.15252/emmm.201505061 (PMC4568953; doi:10.15252/emmm.201505061)
Supplement: Supplementary file 5 [file emmm0007-1211-sd5.pdf]

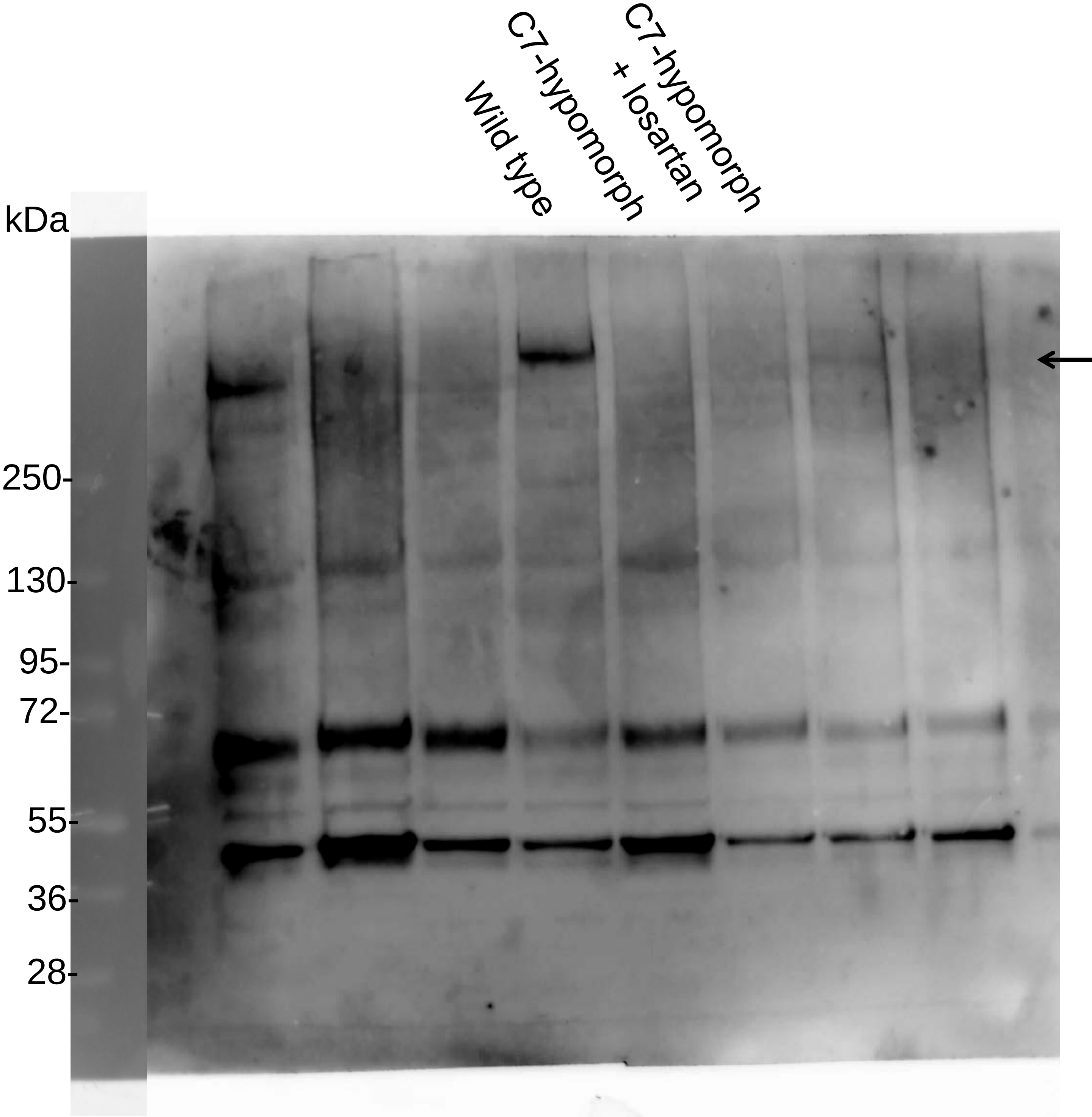
Wild type
C7-hypomorph
C7-hypomorph
+ losartan
kDa
250-
130-
95-
72-
55-
36-
28-

Figure 5A. C7

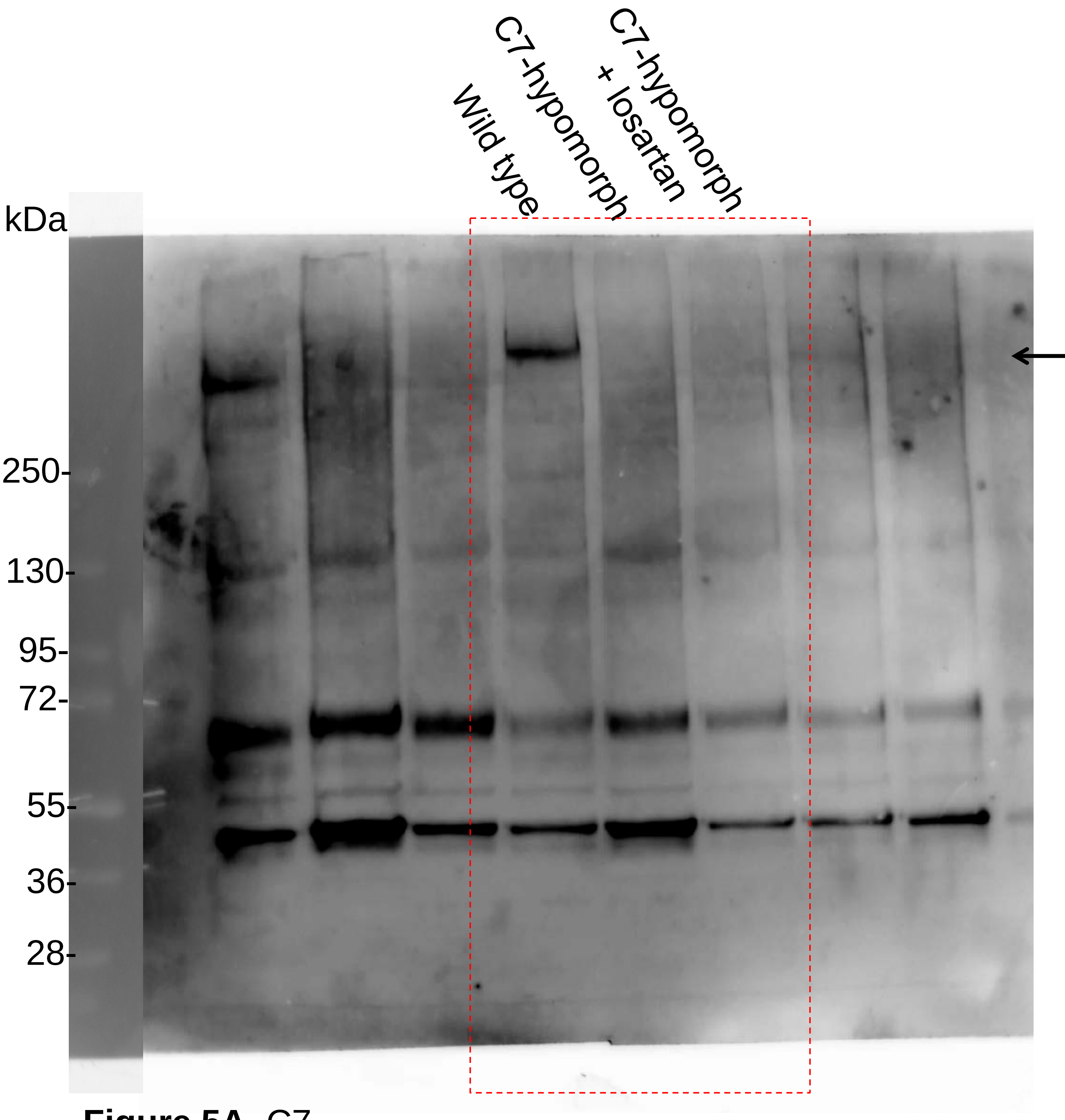

kDa
250-
130-
95-
72-
55-
36-
28-
Wild type
C7-hypomorph
C7-hypomorph
+ losartan

Figure 5A. C7

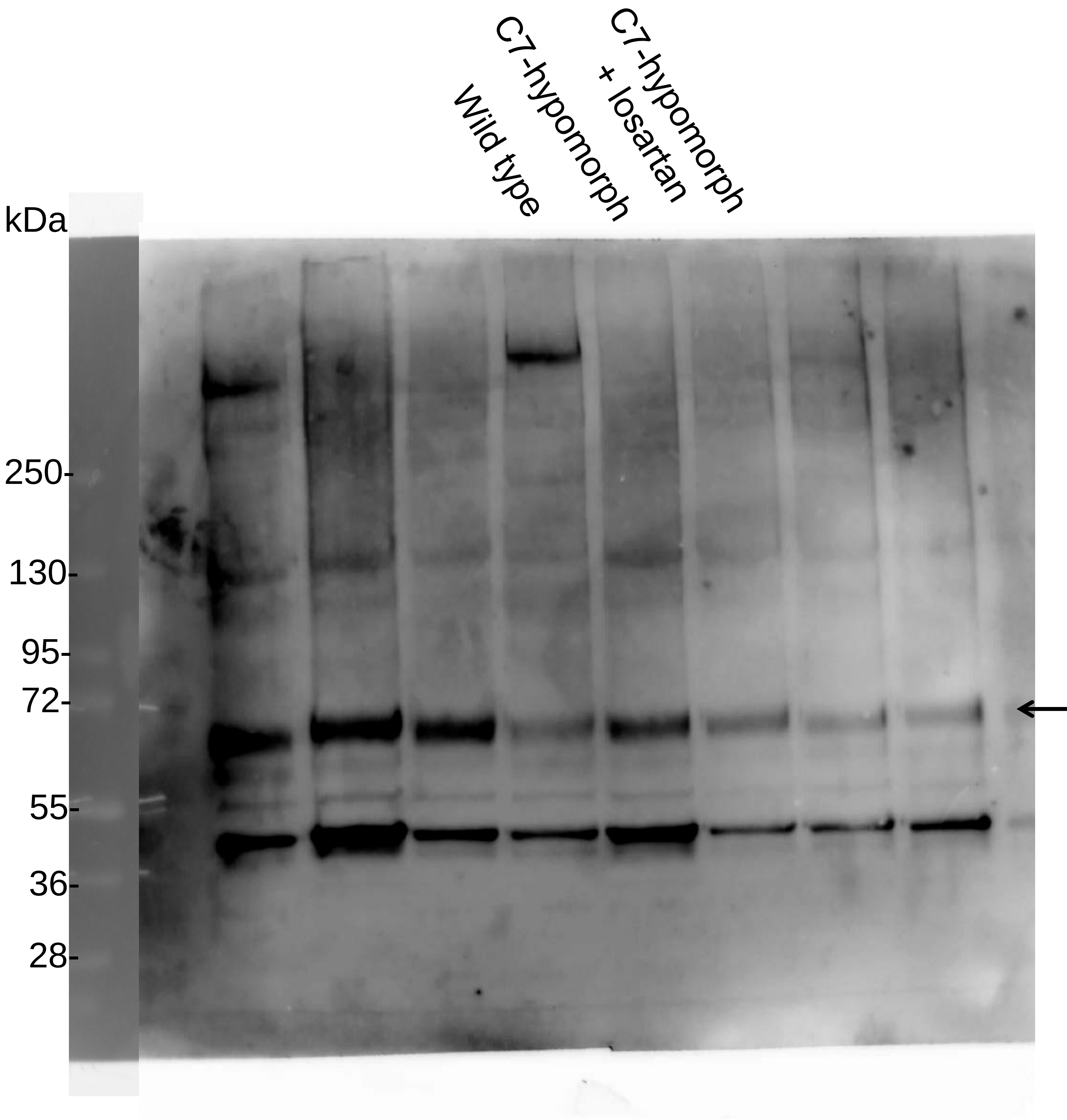

Figure 5A. Tgfbr2

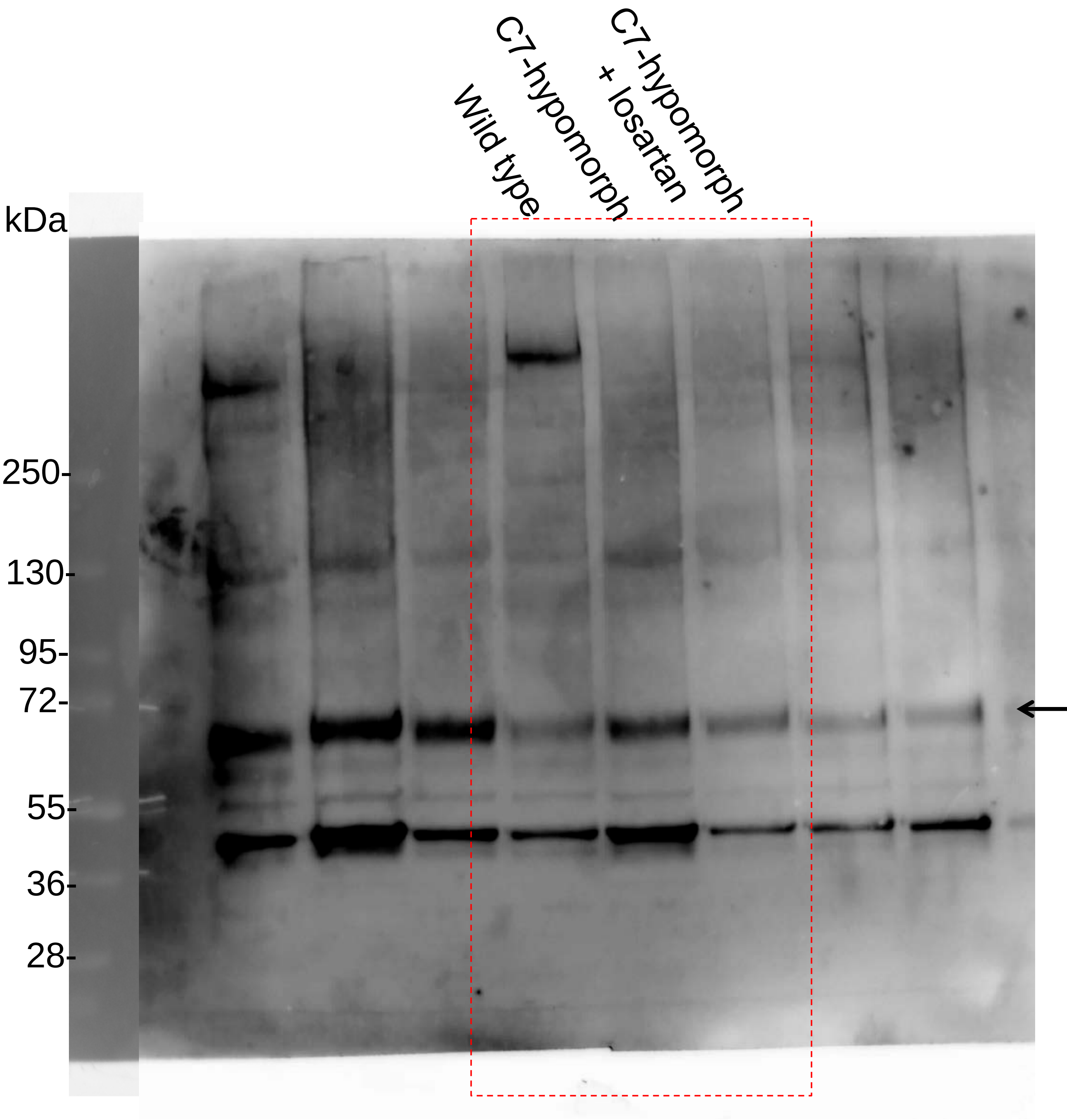

Figure 5A. Tgfbr2

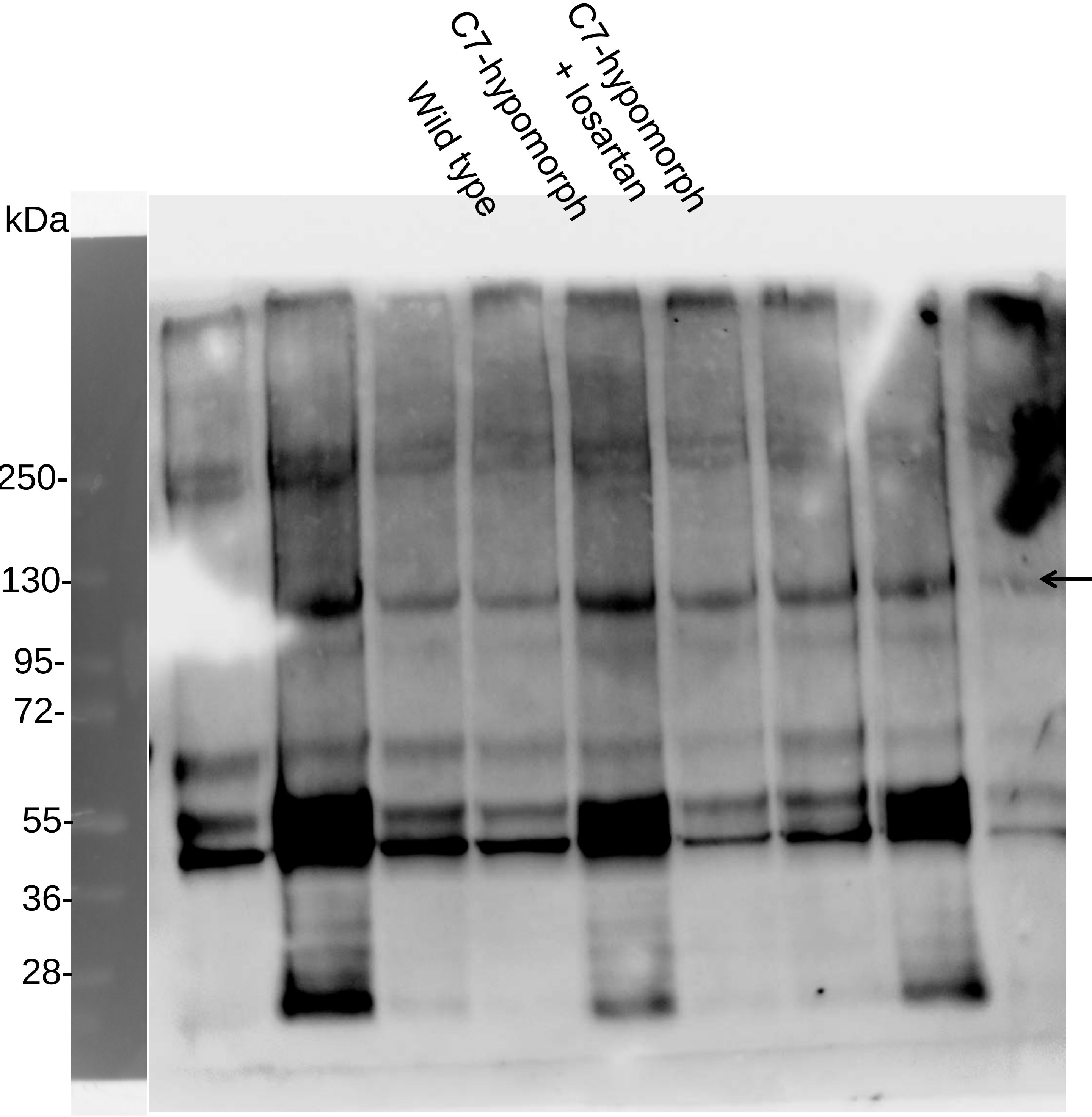

Figure 5A. Tsp1

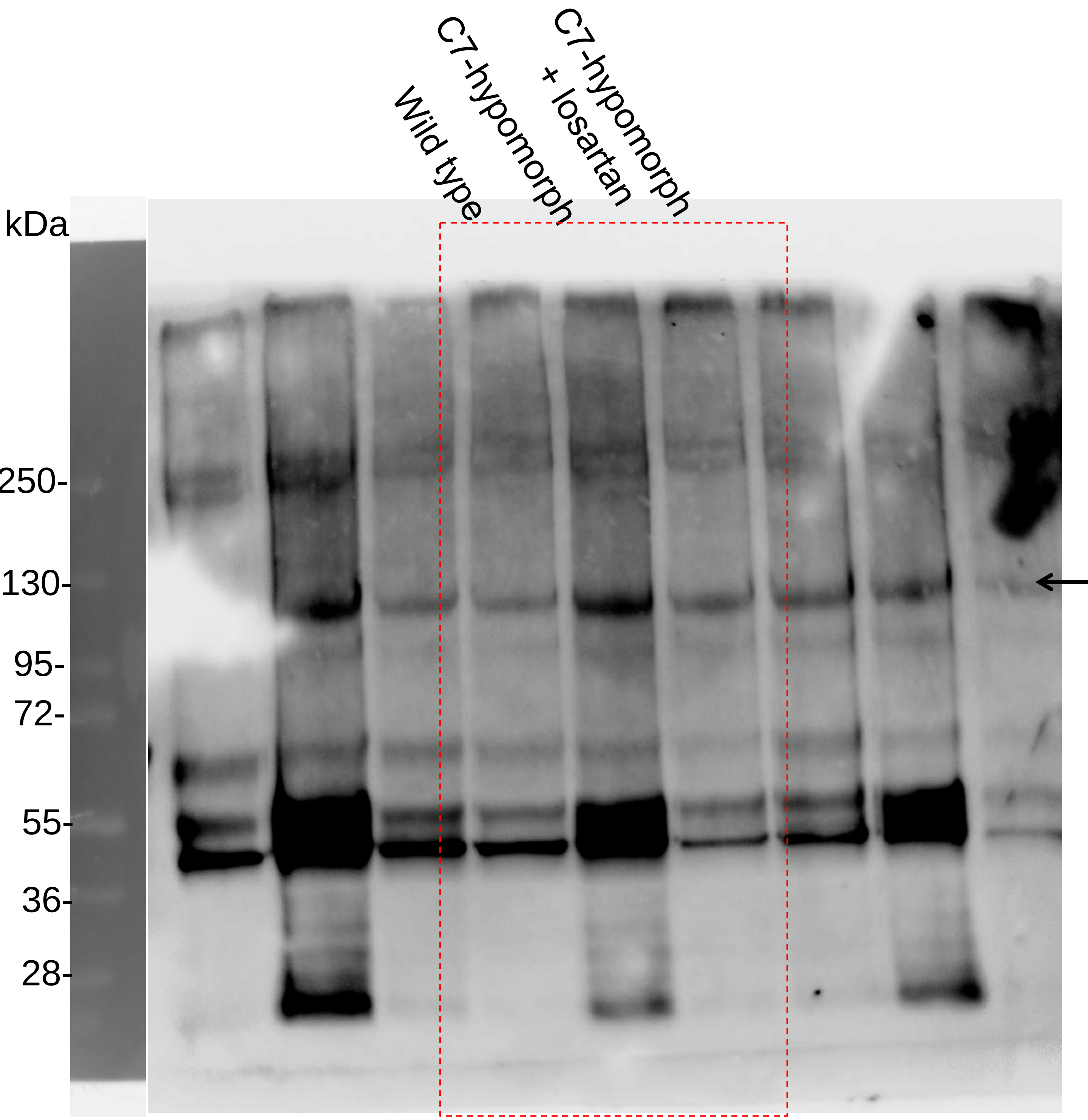

Figure 5A. Tsp1

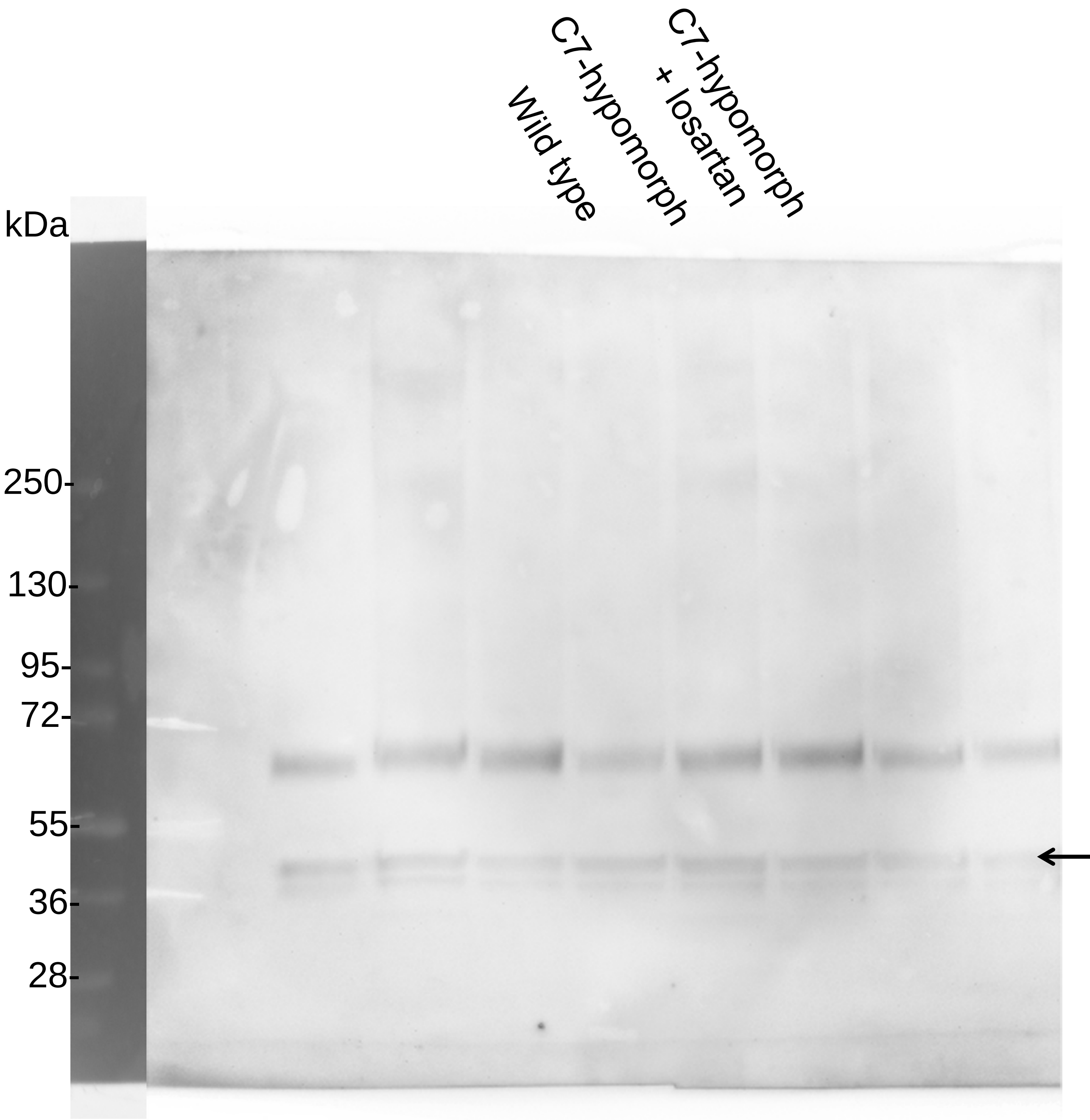

Figure 5A. Erk1/2

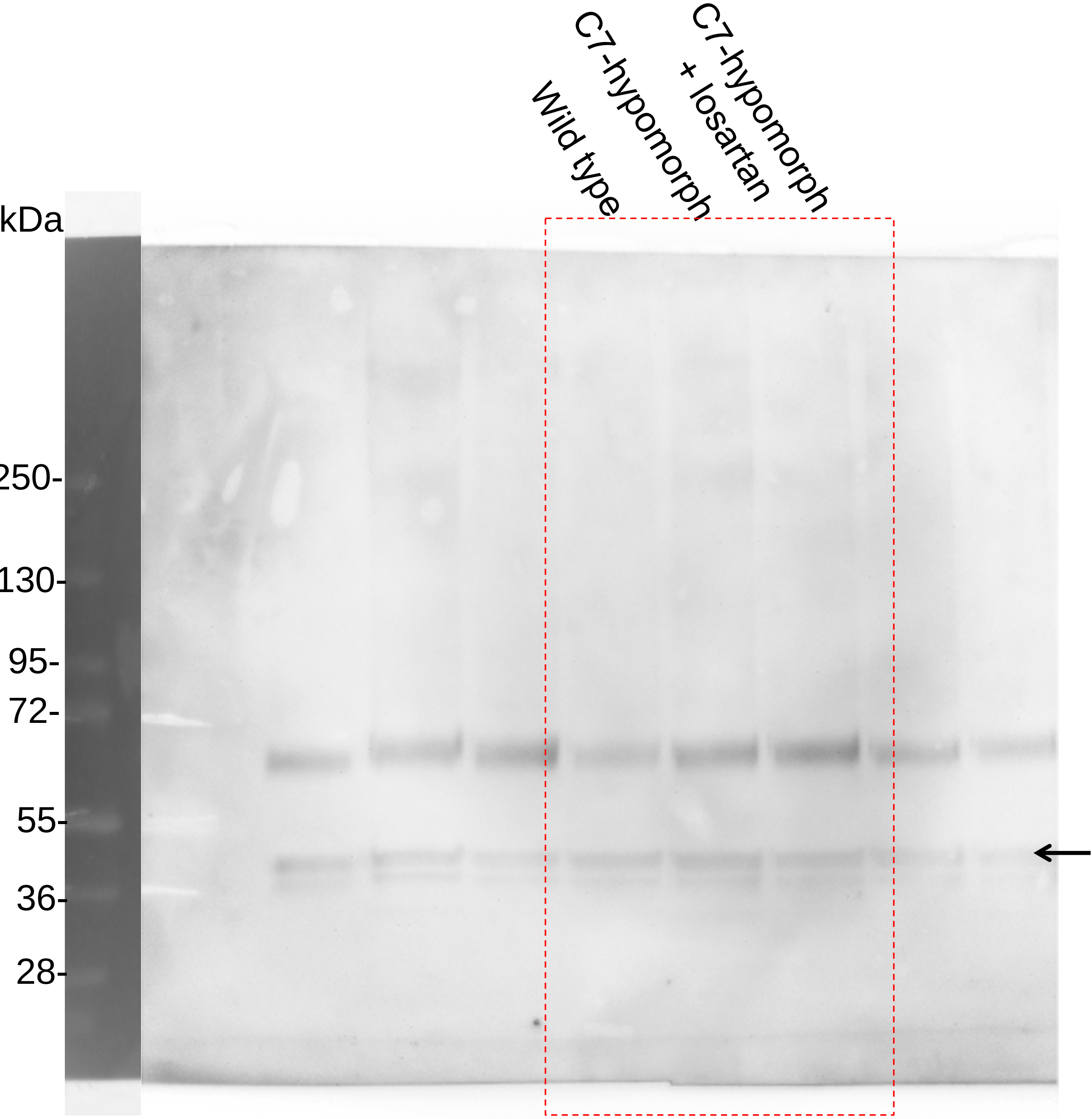

Figure 5A. Erk1/2

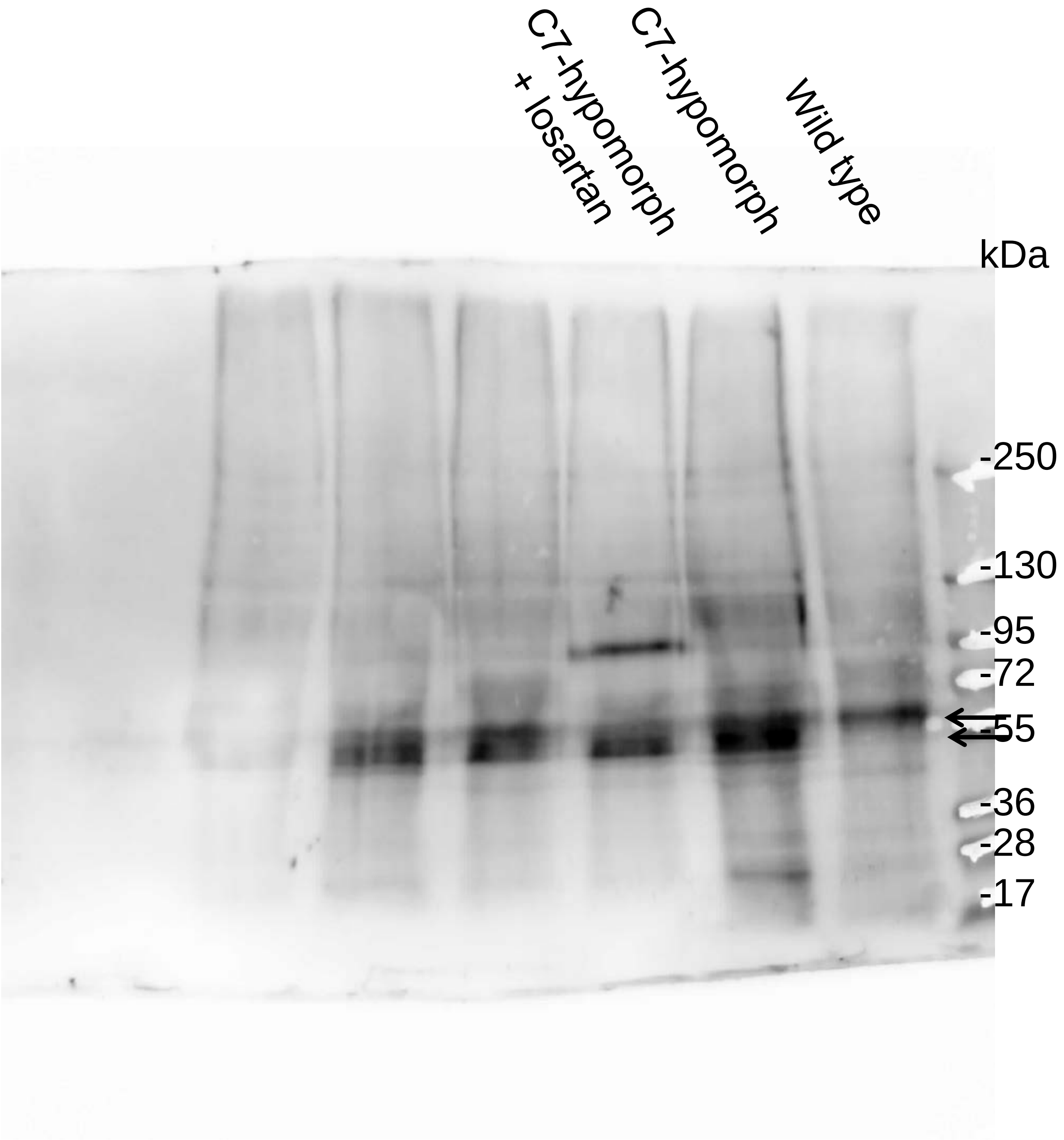

Figure 5A. P-Smad2/3

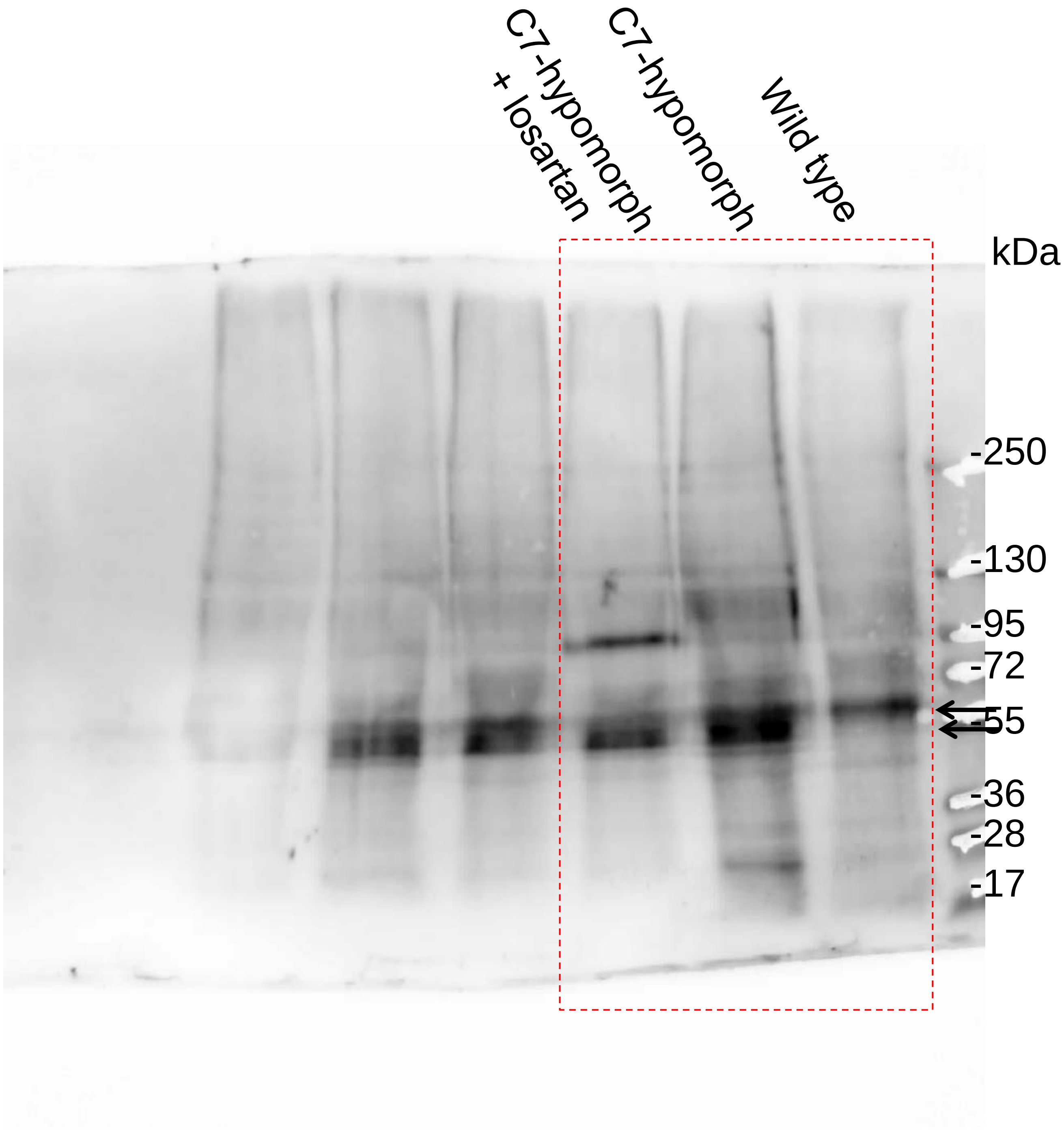

Figure 5A. P-Smad2/3

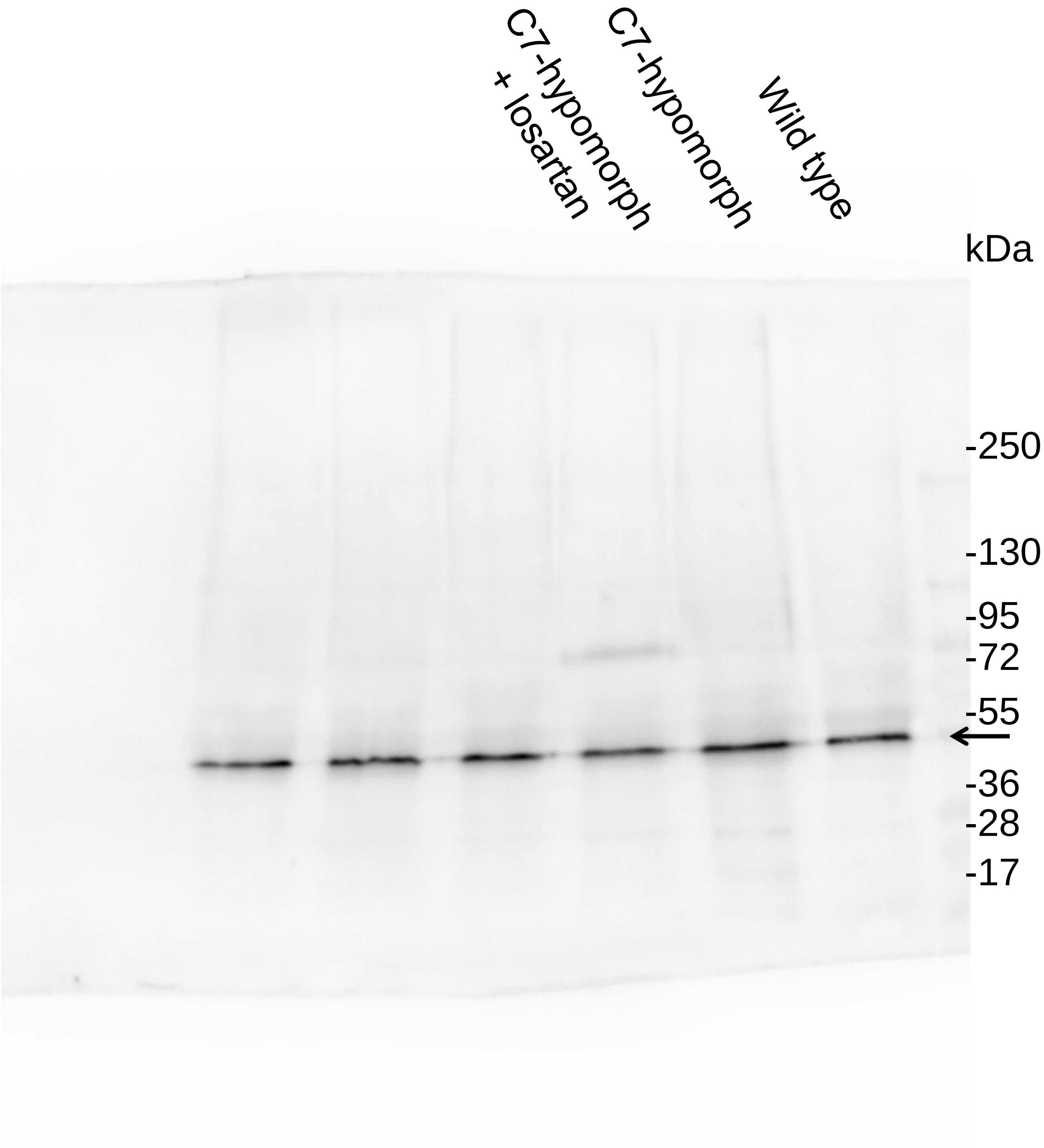

Figure 5A. β-tubulin

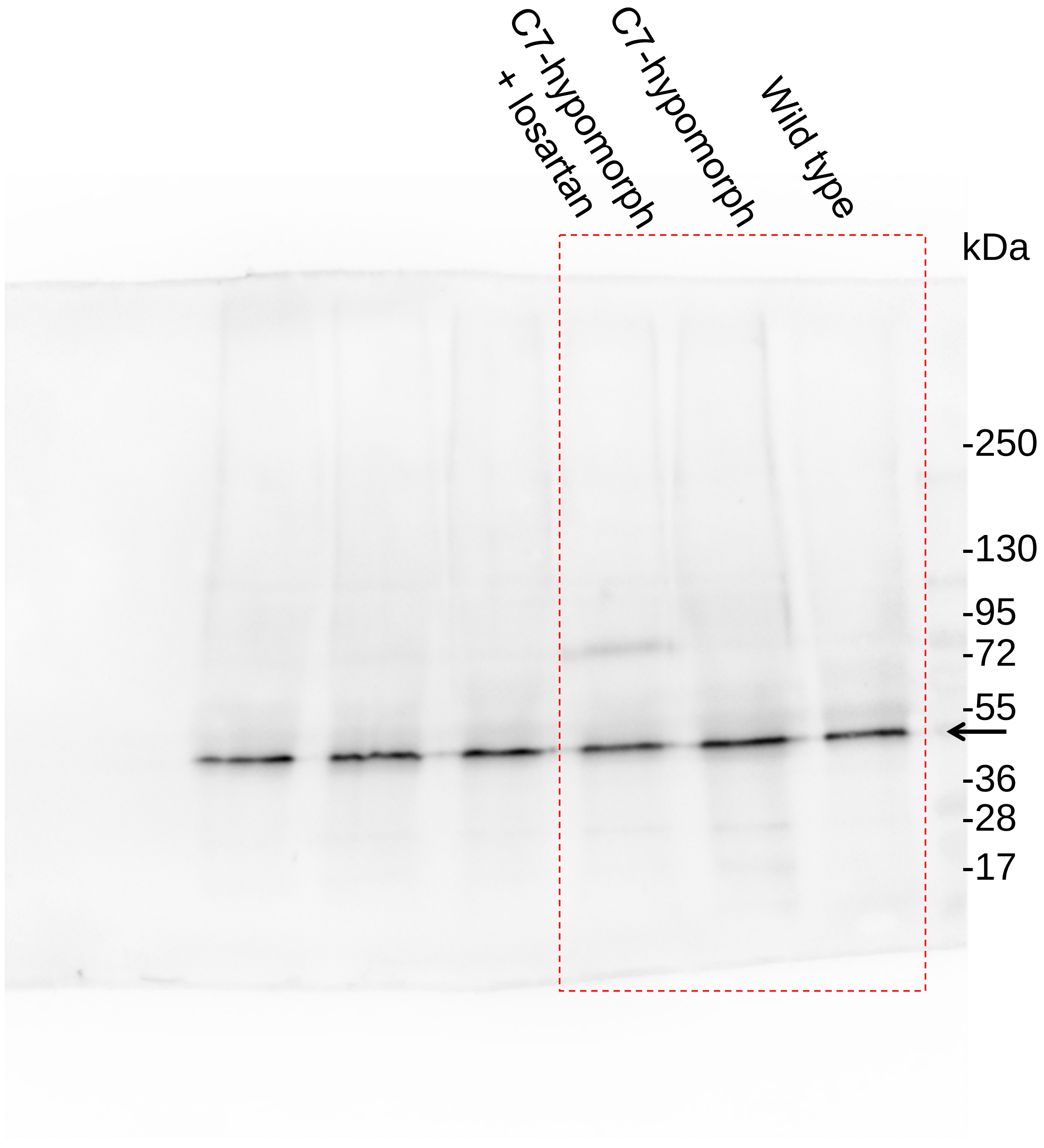

Figure 5A. β-tubulin

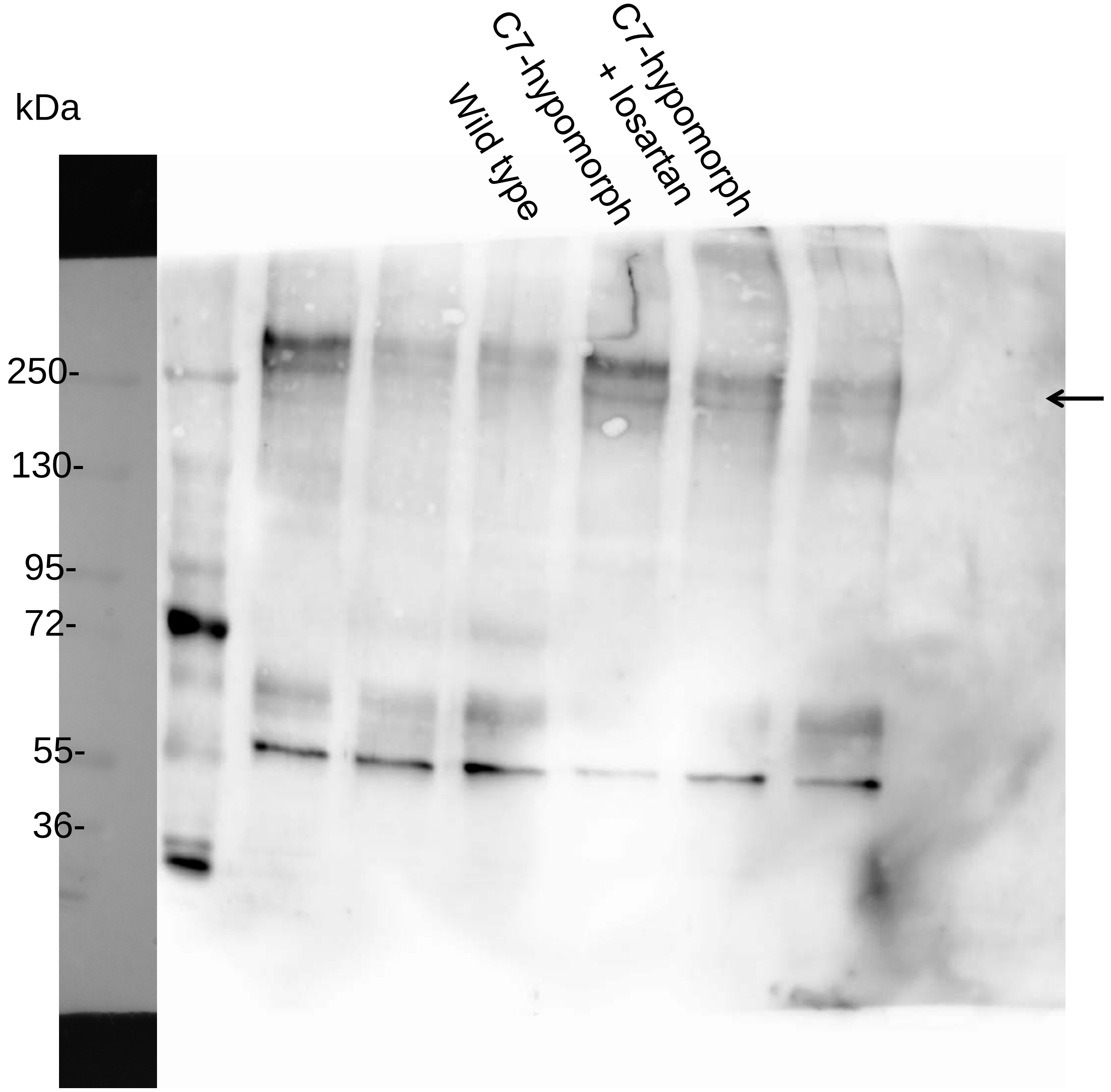

Figure 5A. Fibronectin

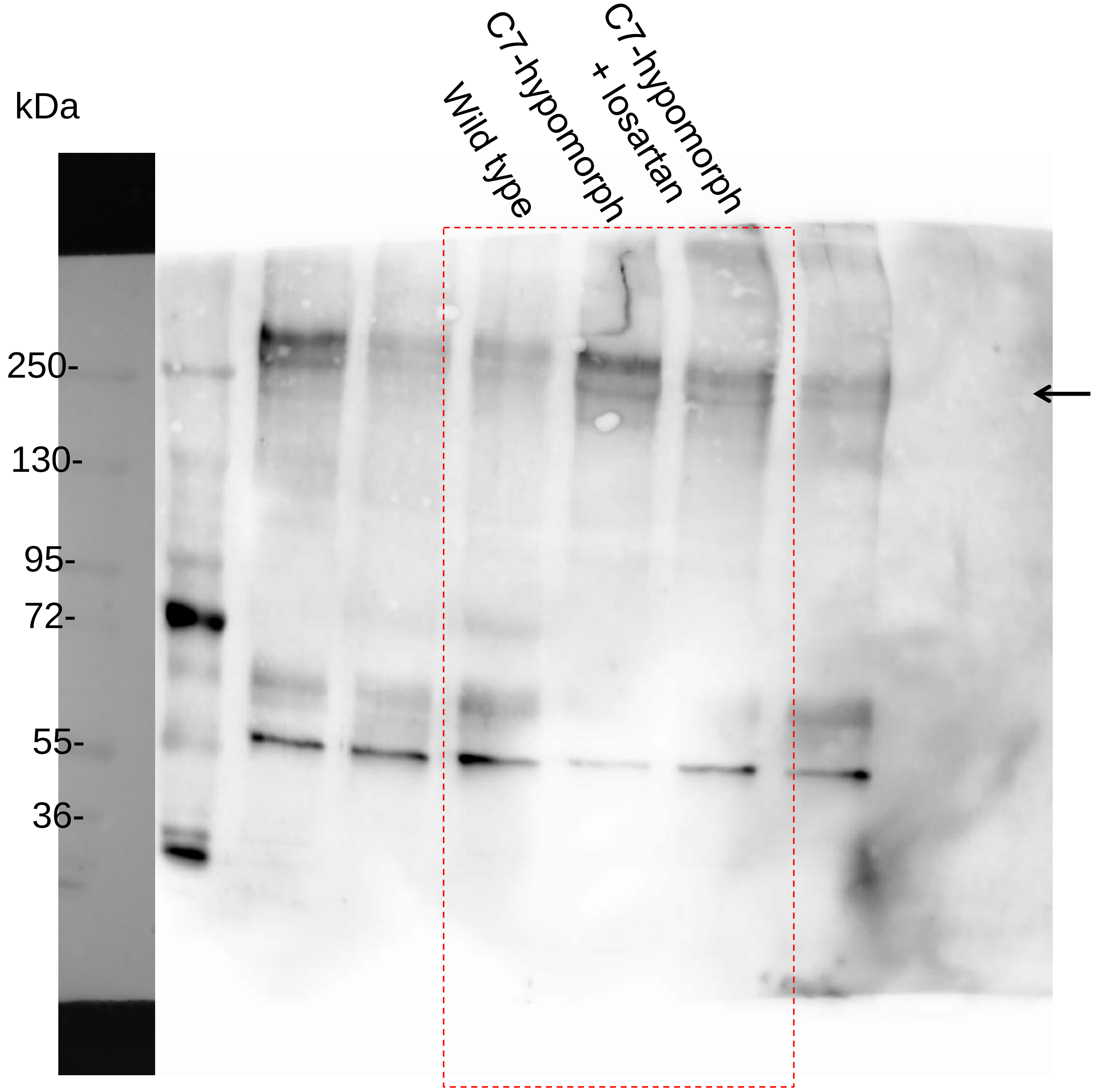

Figure 5A. Fibronectin

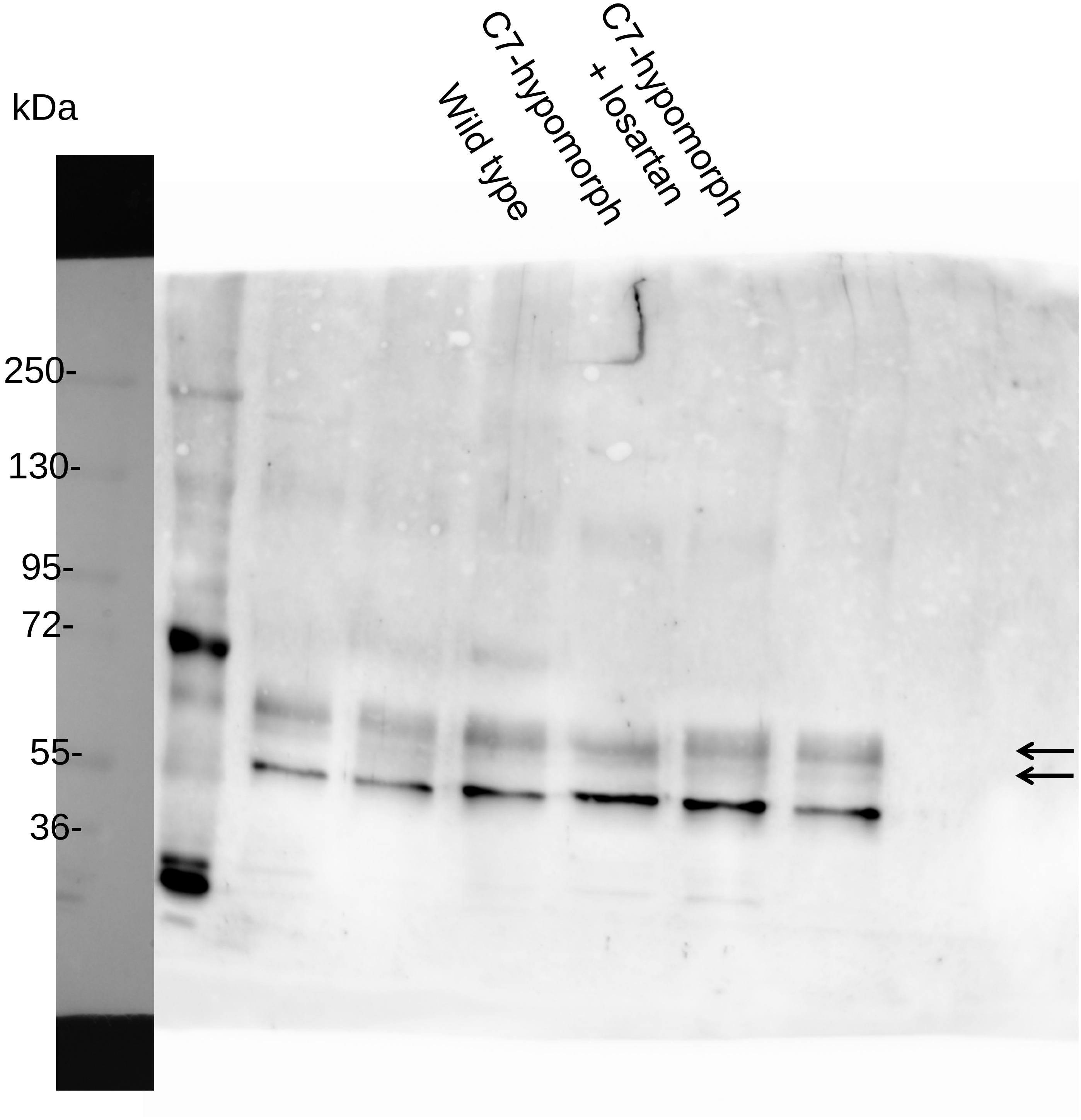

Figure 5A. Smad2/3

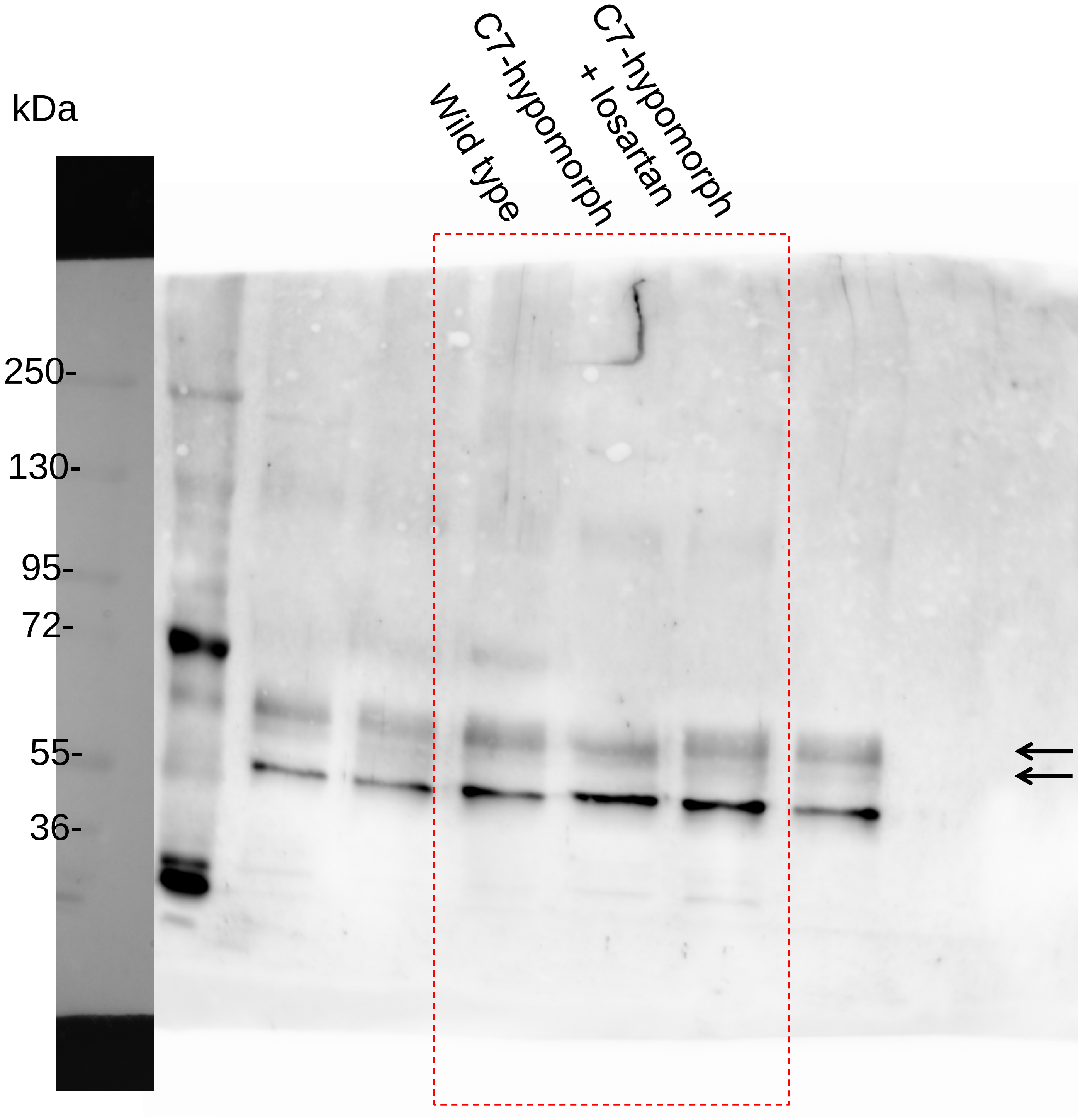

Figure 5A. Smad2/3

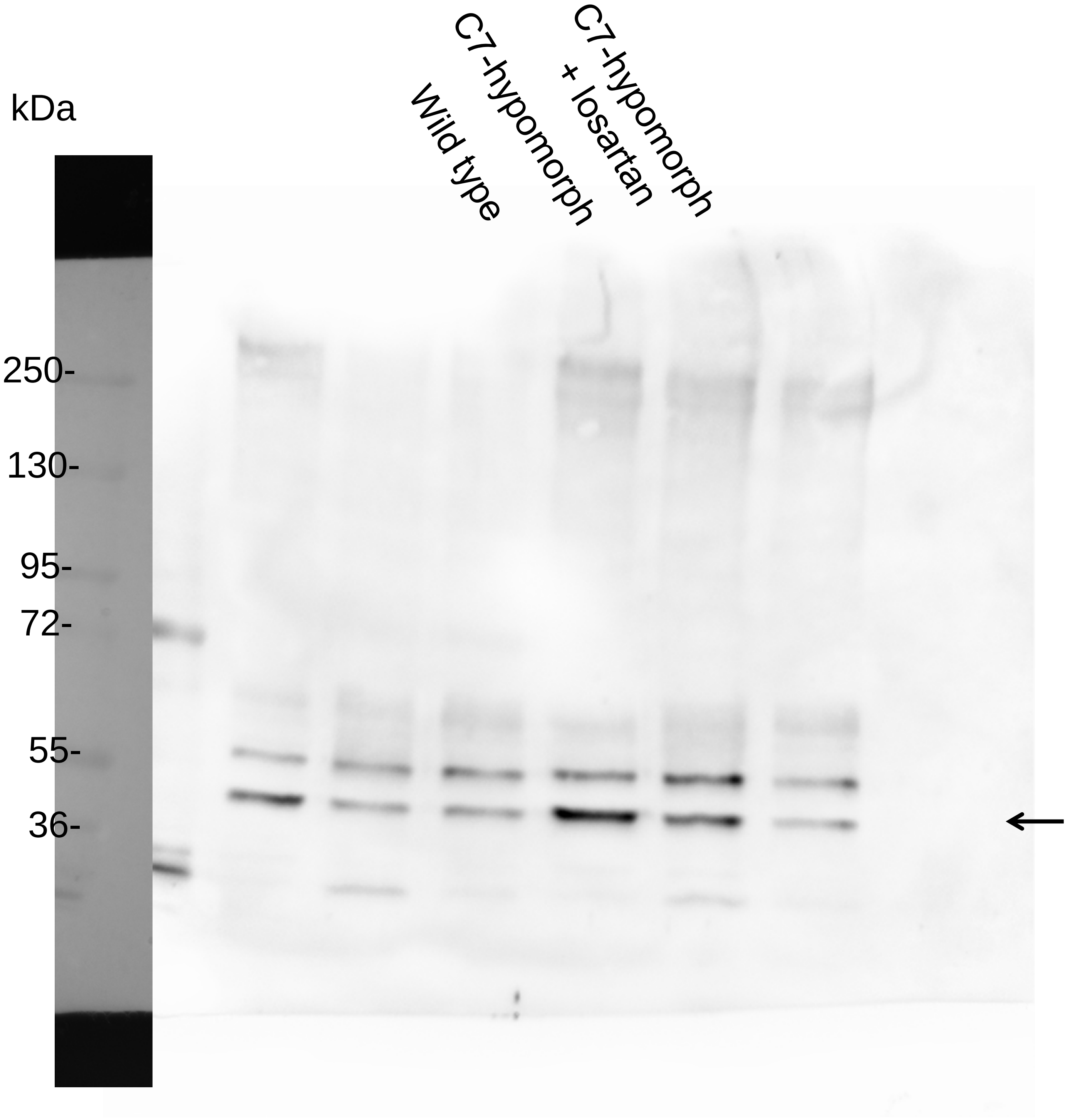

Figure 5A. αSma

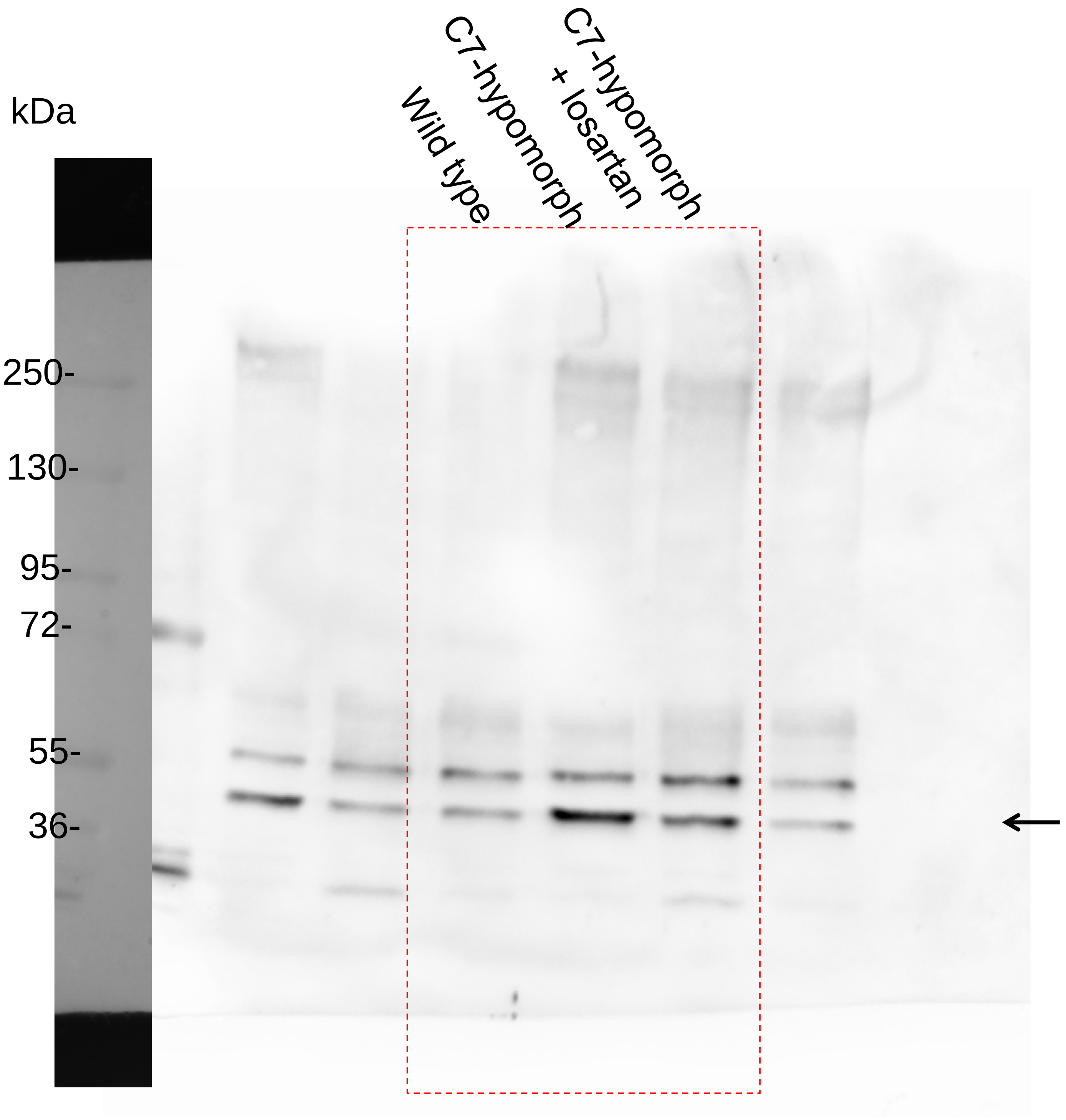

Figure 5A. αSma

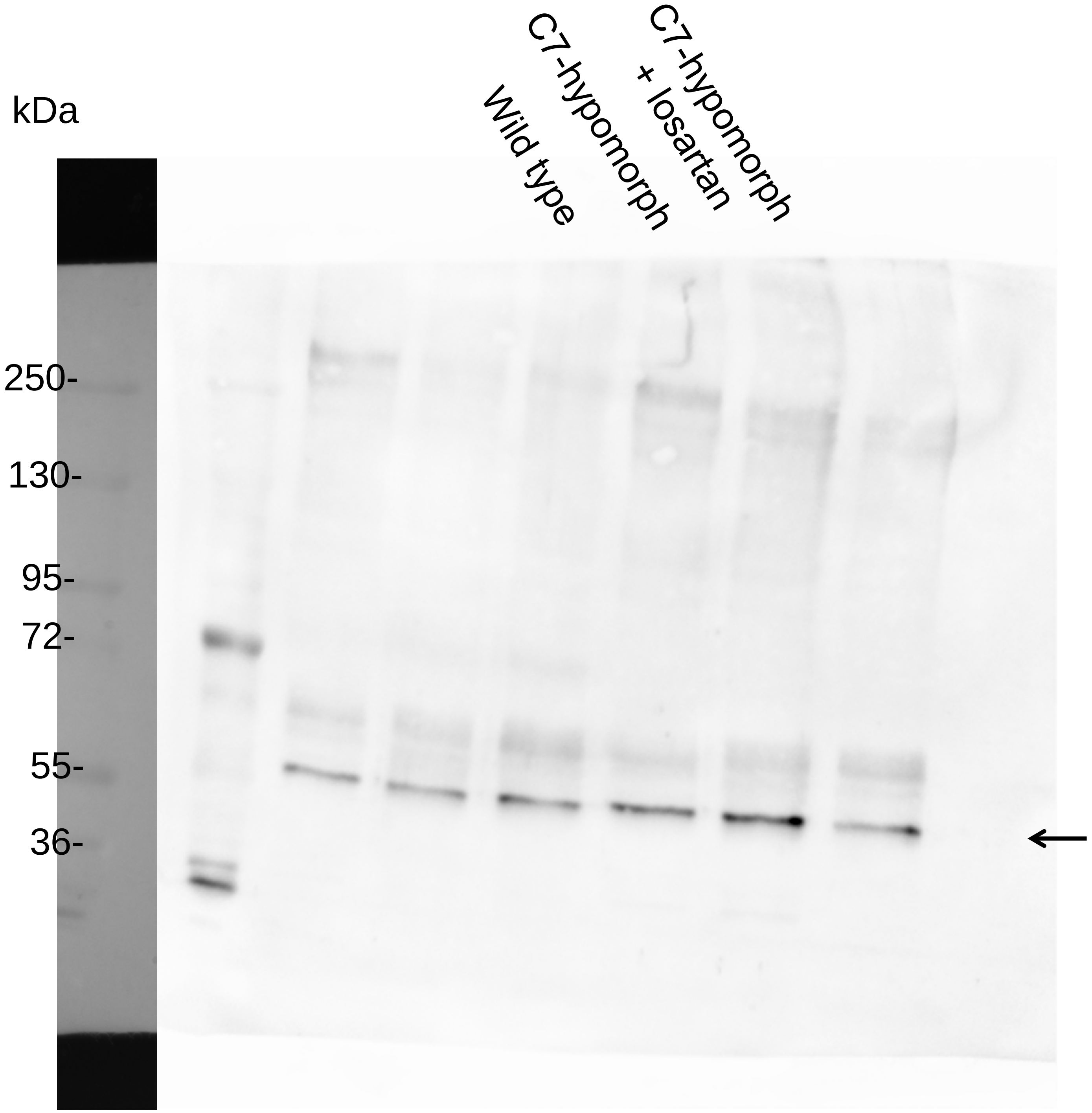

Figure 5A. β-tubulin

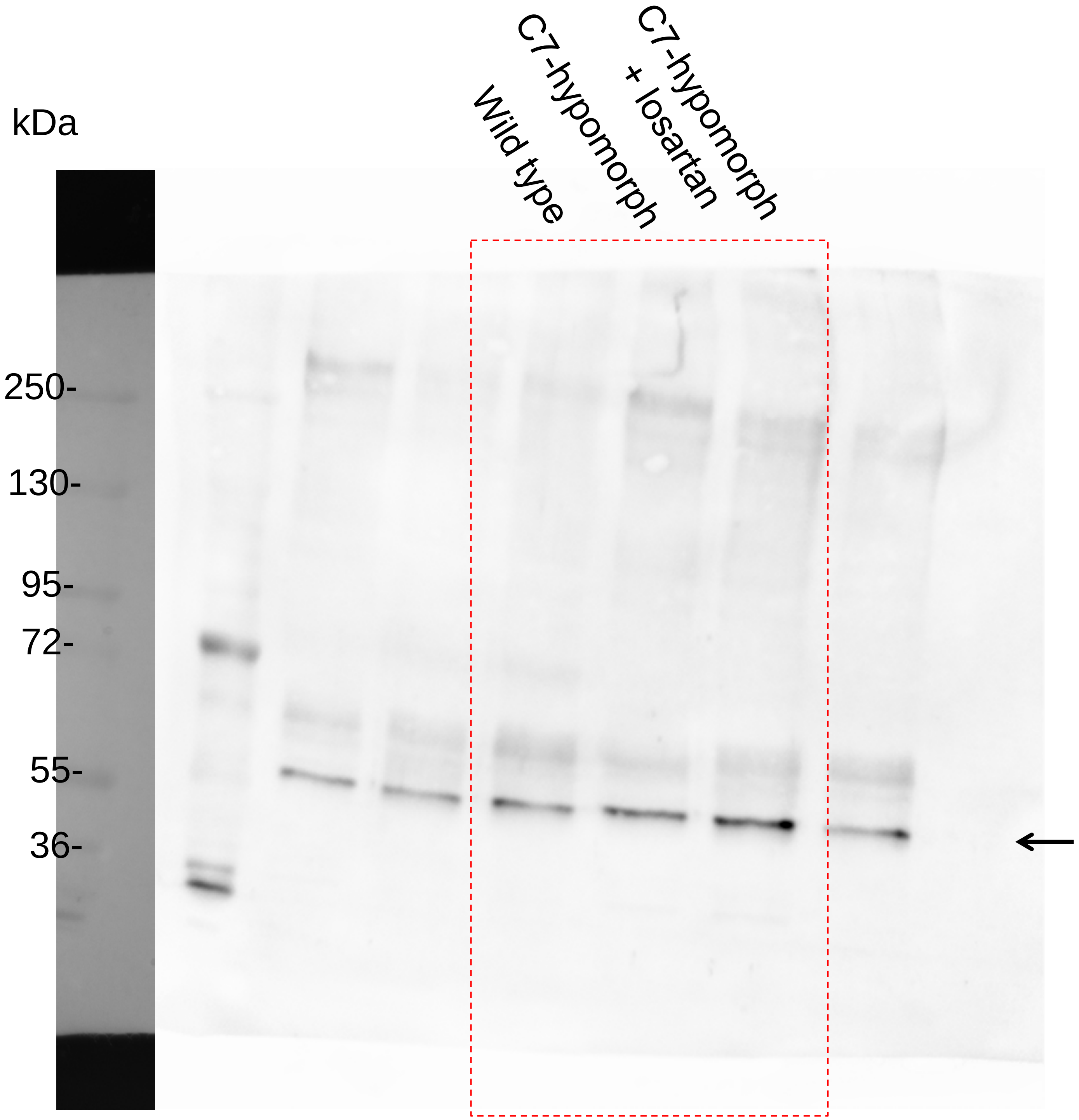

Figure 5A. β-tubulin

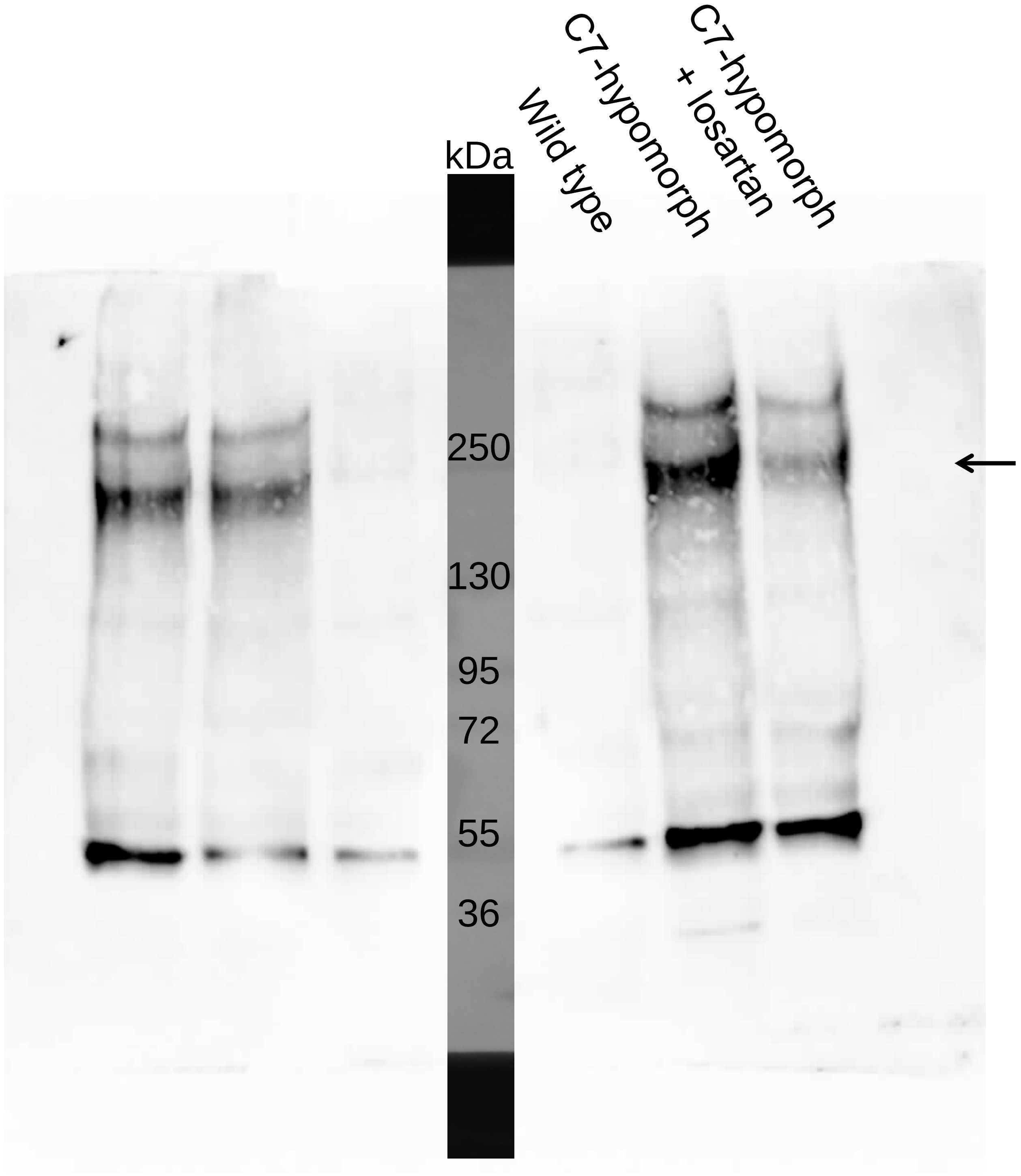

Figure 5A. Tenascin-C

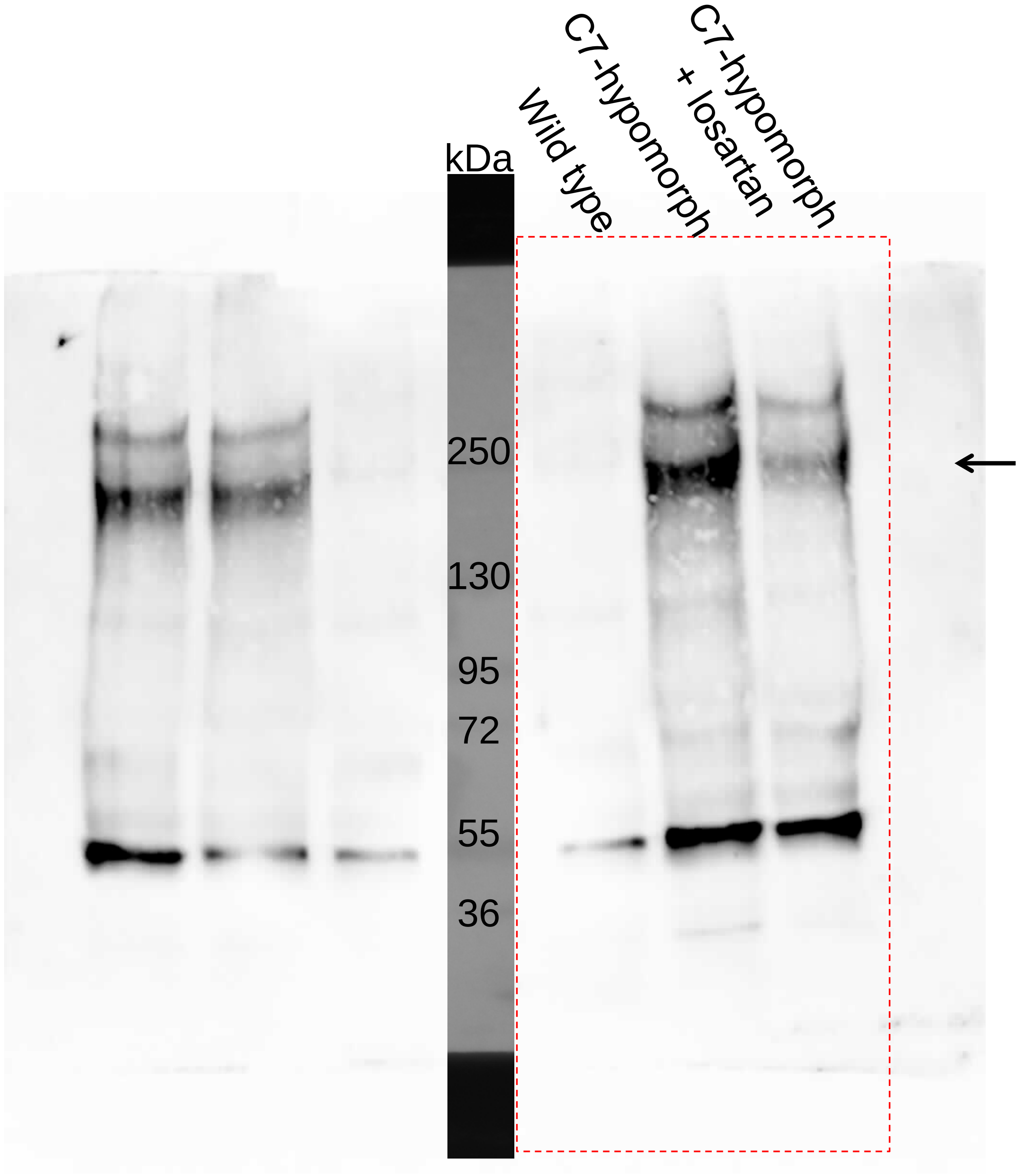

Figure 5A. Tenascin-C

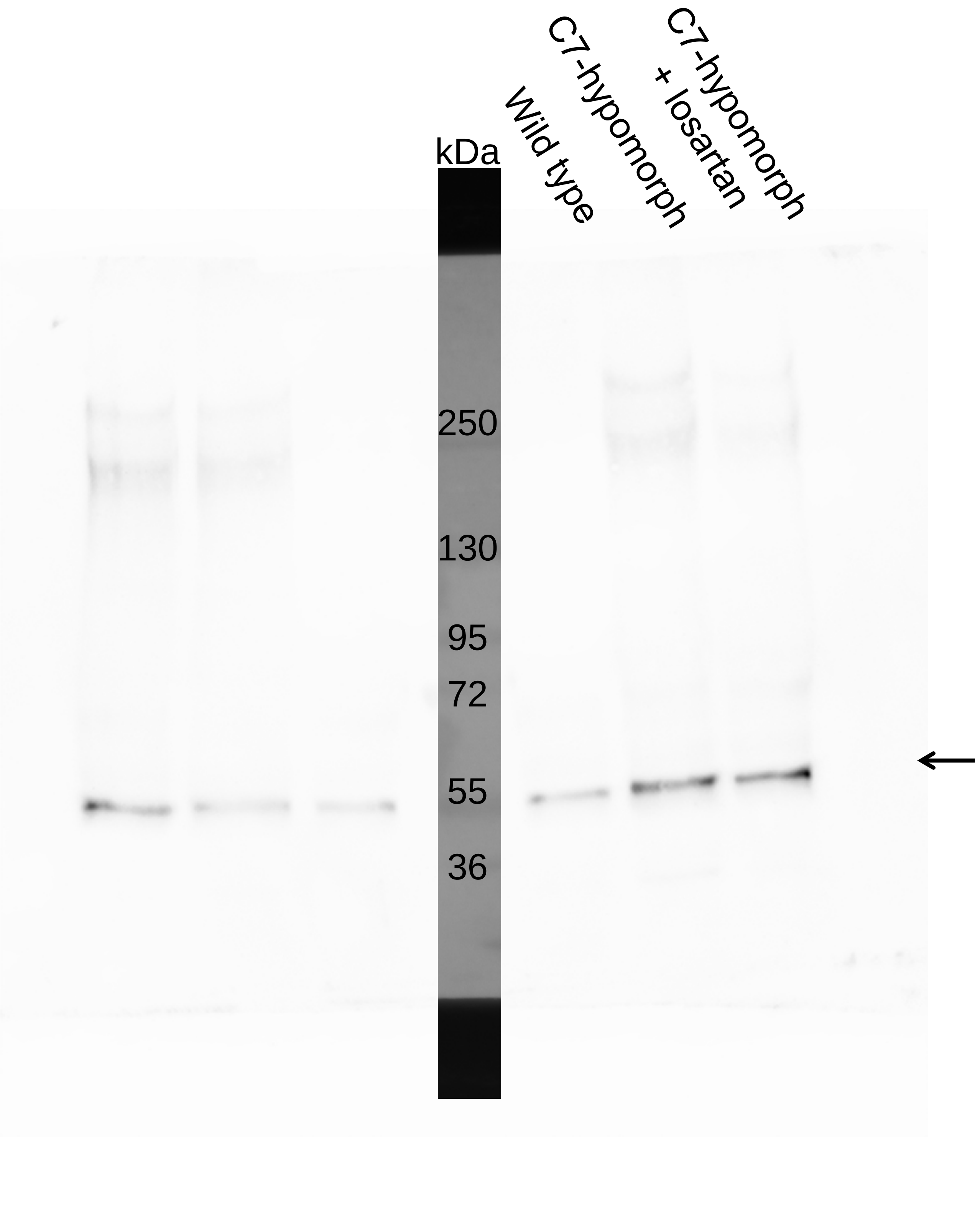

Figure 5A. β-tubulin

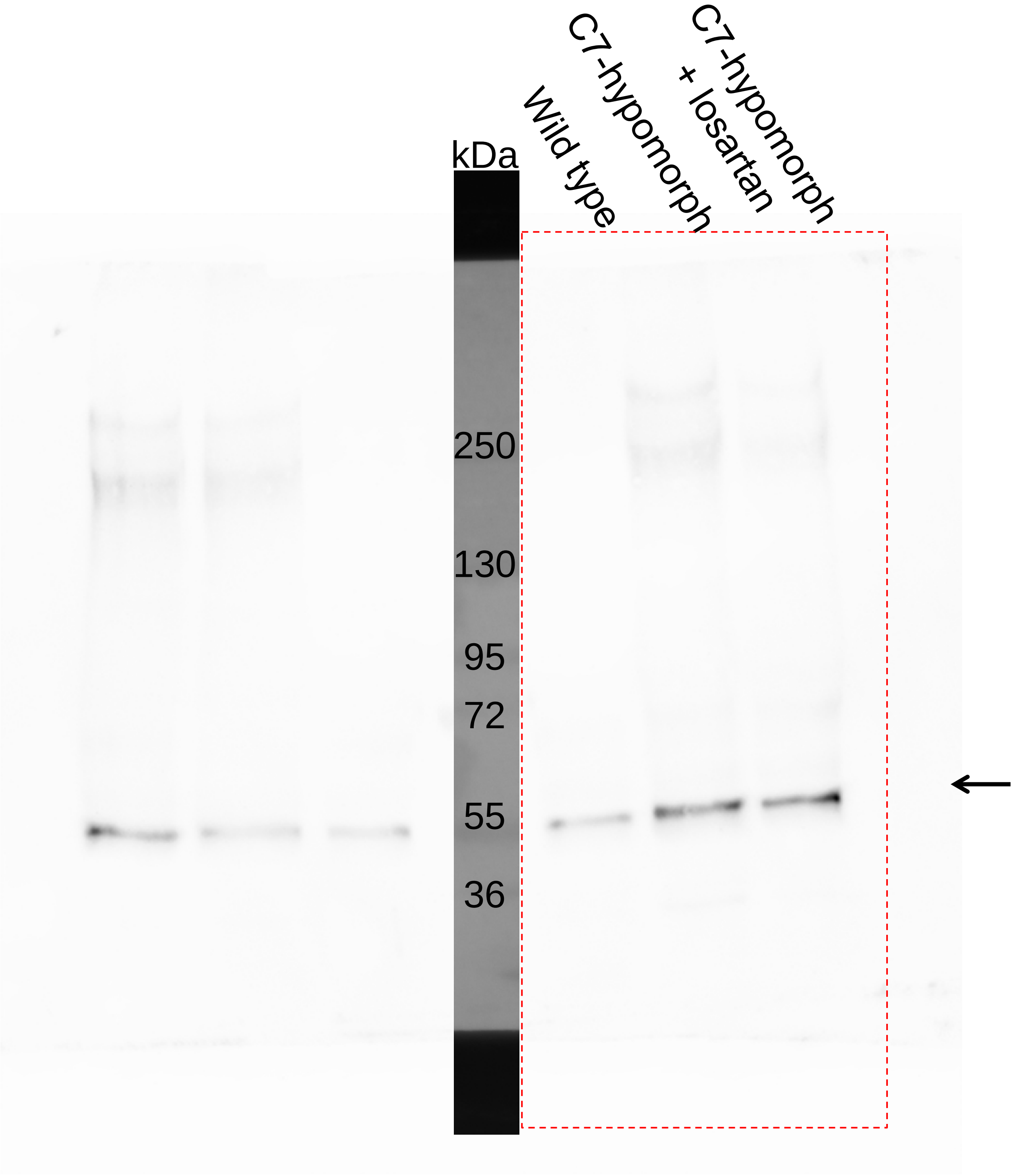

Figure 5A. β-tubulin
