# Supplementary material for: Losartan ameliorates dystrophic epidermolysis bullosa and uncovers new disease mechanisms
Source: EMBO Mol Med. 2015 Jul 20;7(9):1211–28. doi: 10.15252/emmm.201505061 (PMC4568953; doi:10.15252/emmm.201505061)
Supplement: Supplementary file 6 [file emmm0007-1211-sd6.pdf]

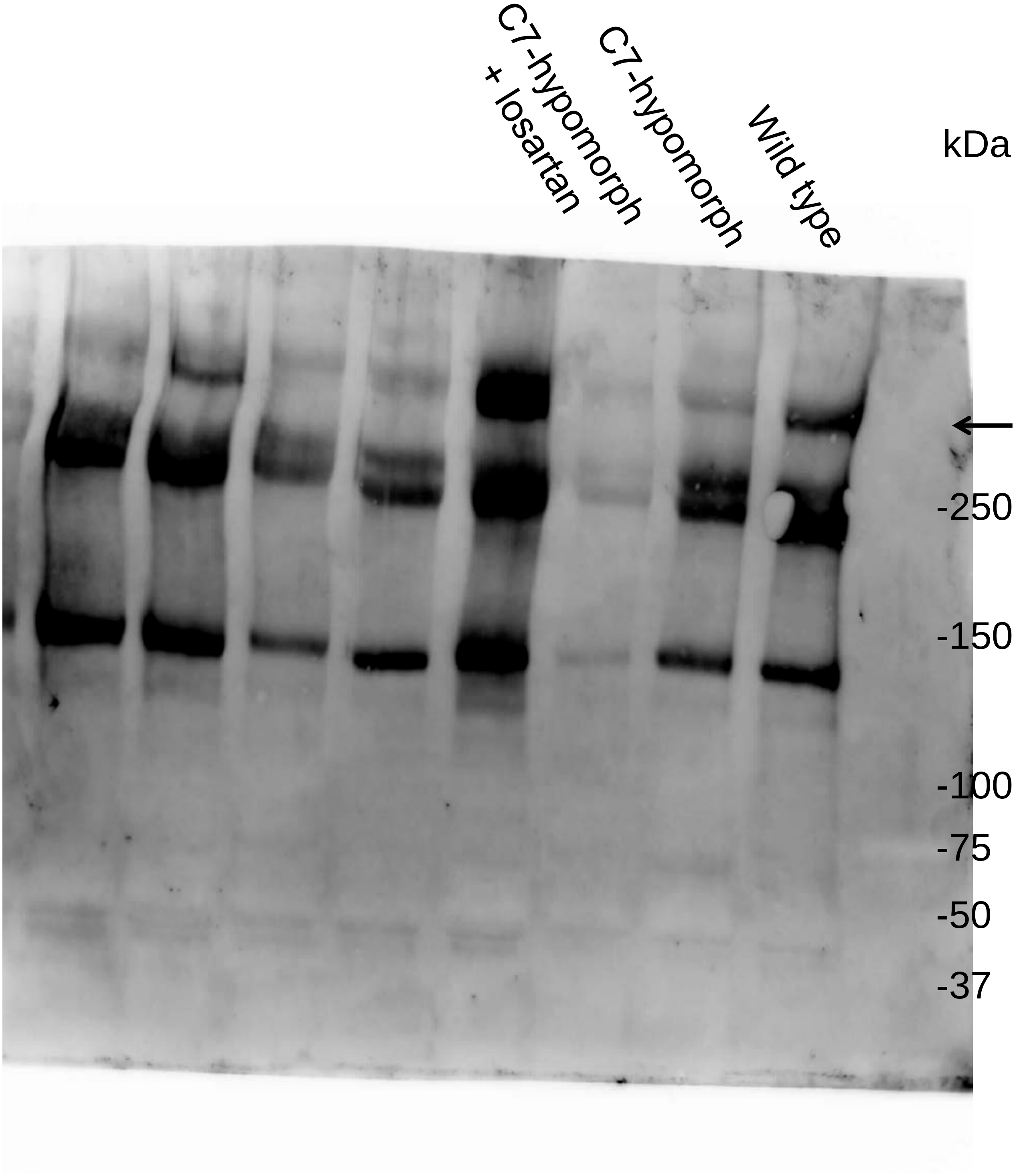
C7-hypomorph + losartan
C7-hypomorph
Wild type
kDa
-250
-150
-100
-75
-50
-37

Figure 6C. C7

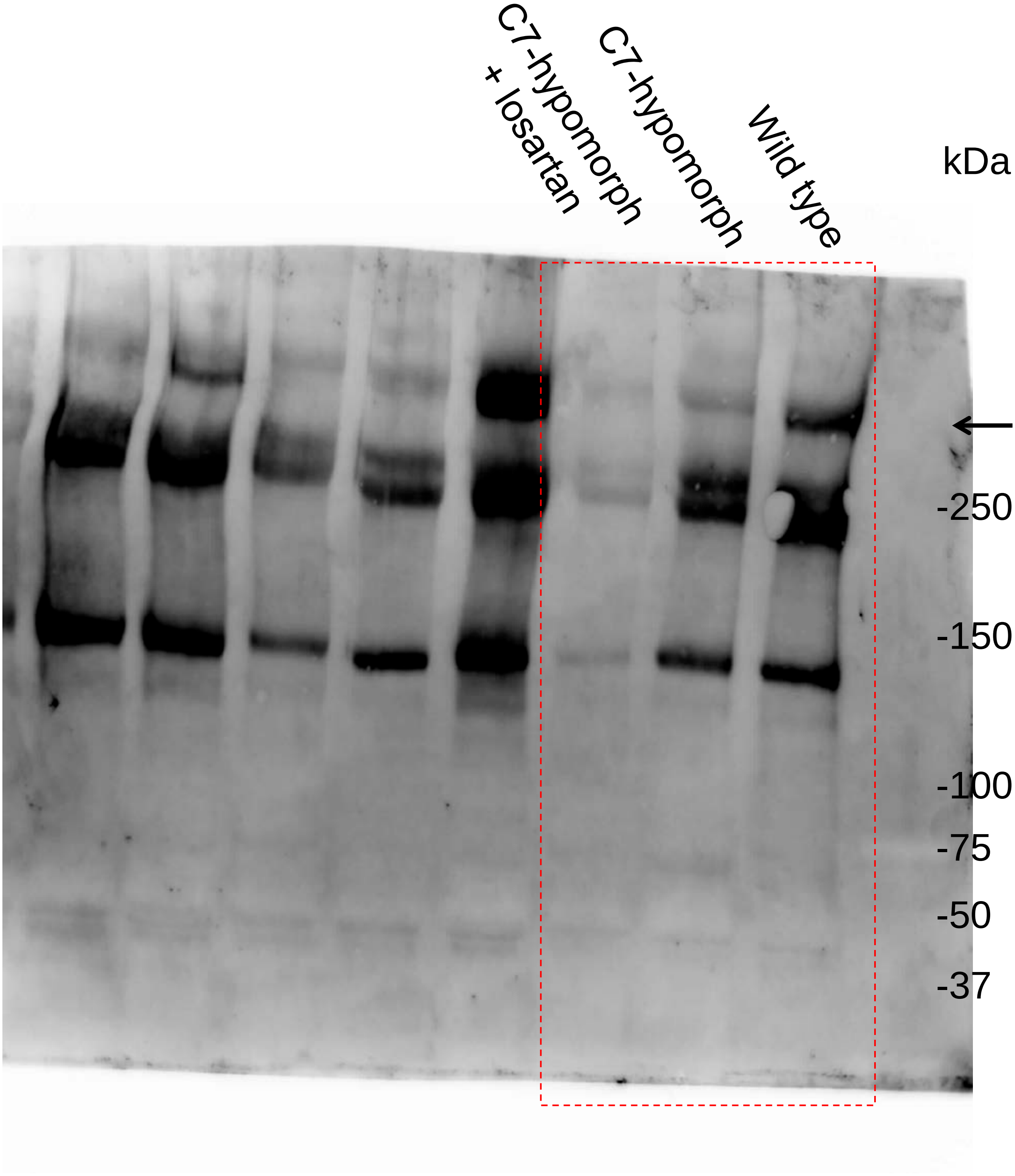
C7-hypomorph
+ losartan
C7-hypomorph
Wild type
kDa
-250
-150
-100
-75
-50
-37

Figure 6C. C7

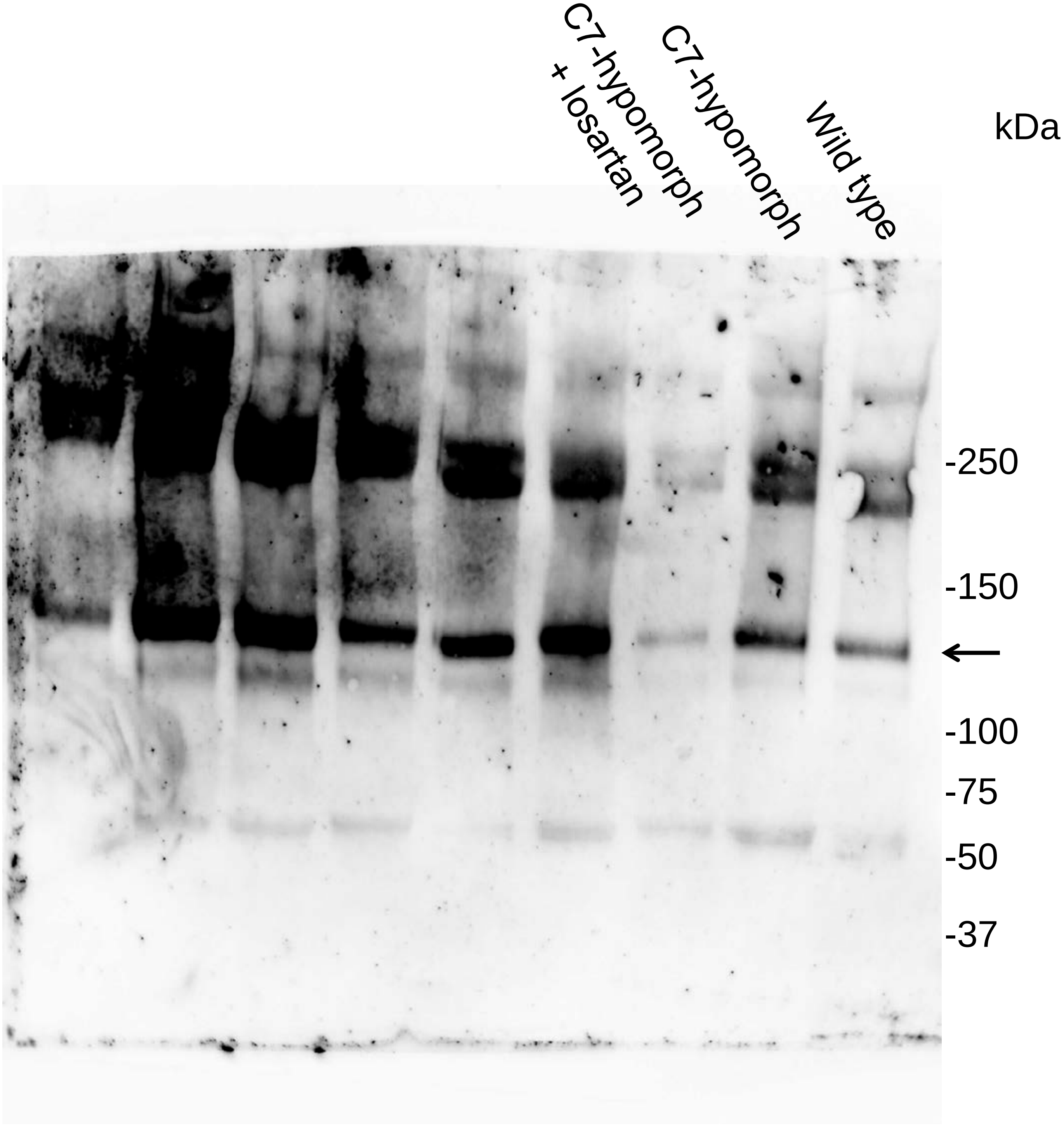

Figure 6C. Lrg1

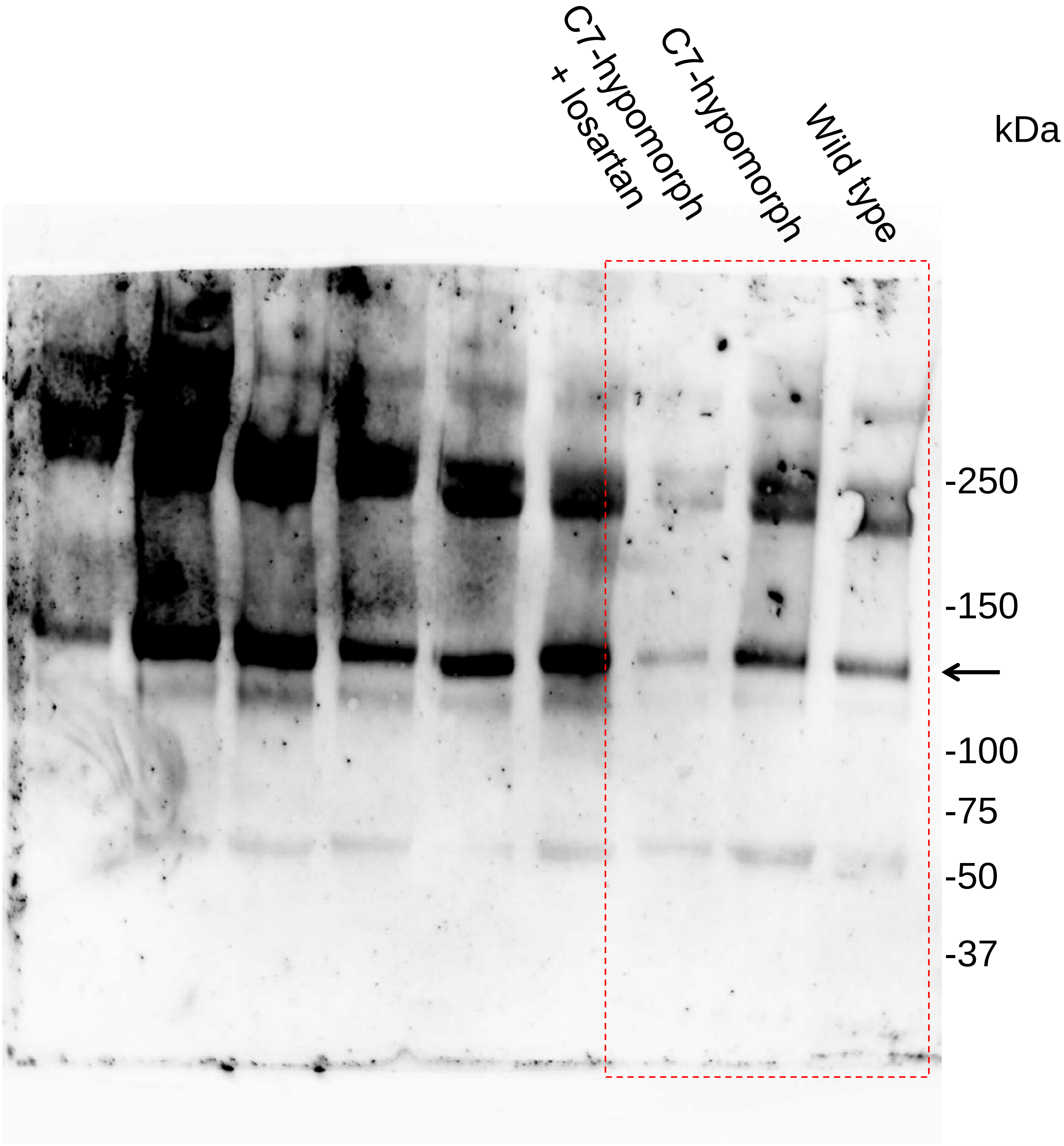

Figure 6C. Lrg1

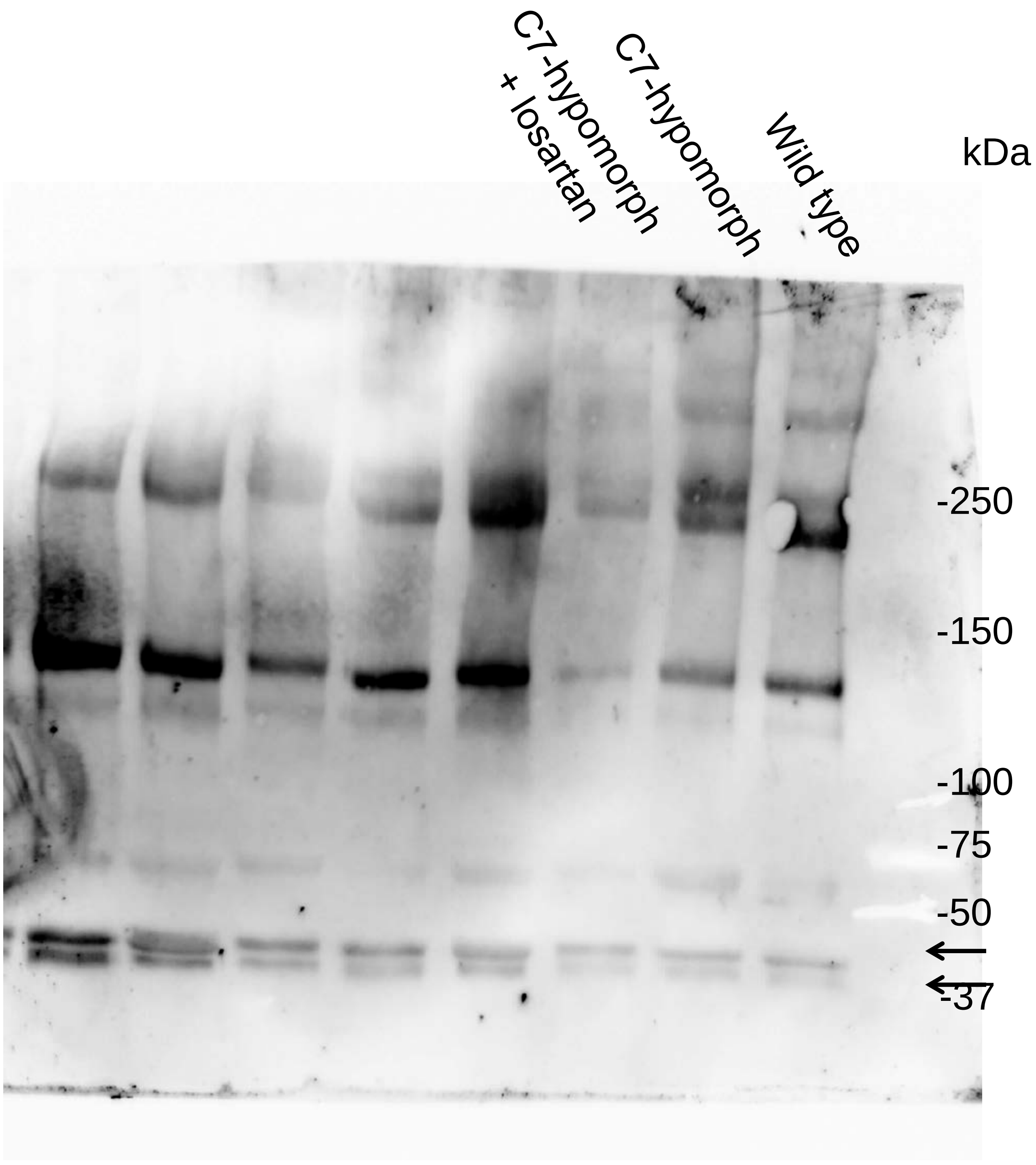

Figure 6C. Erk1/2

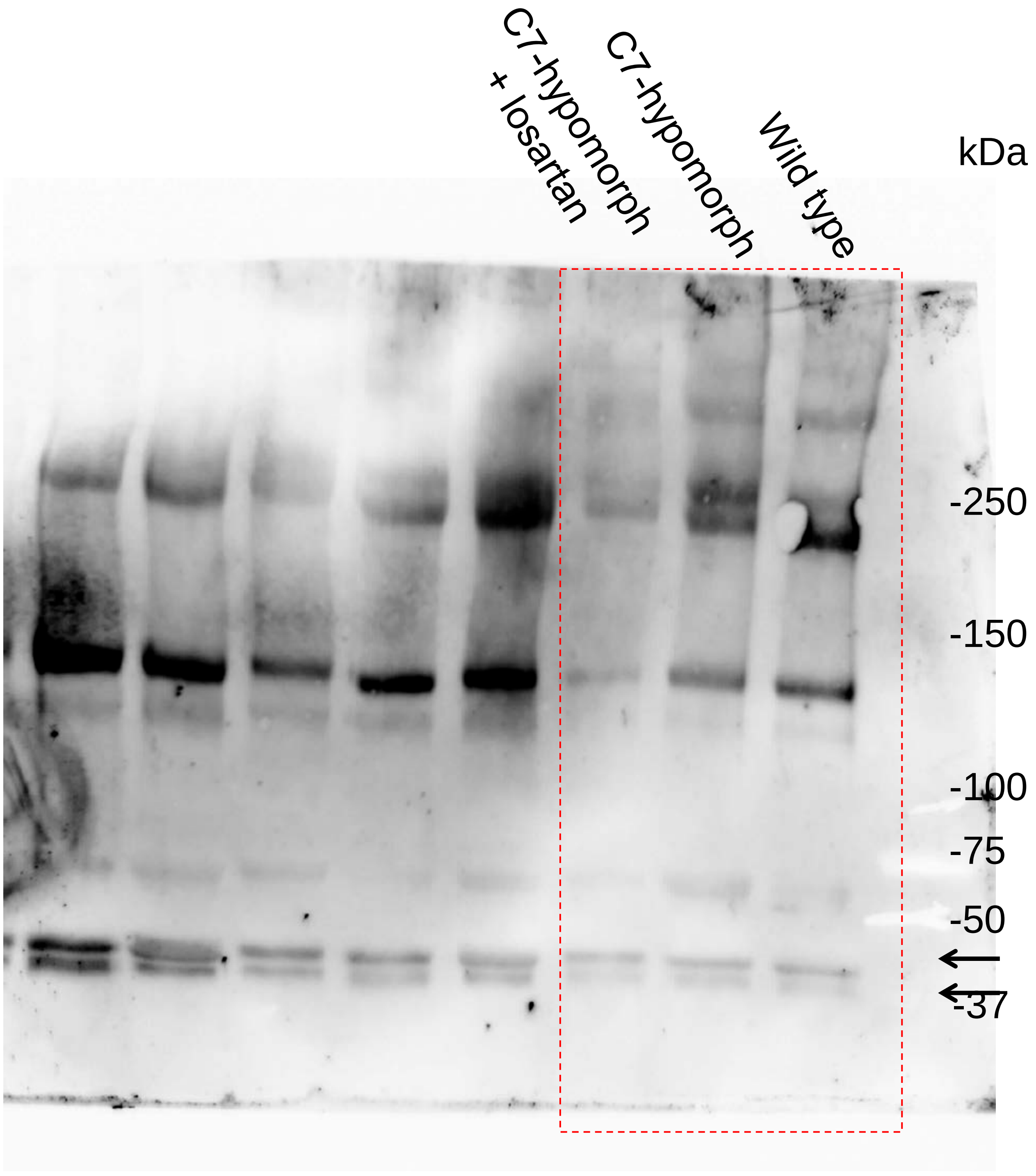

Figure 6C. Erk1/2

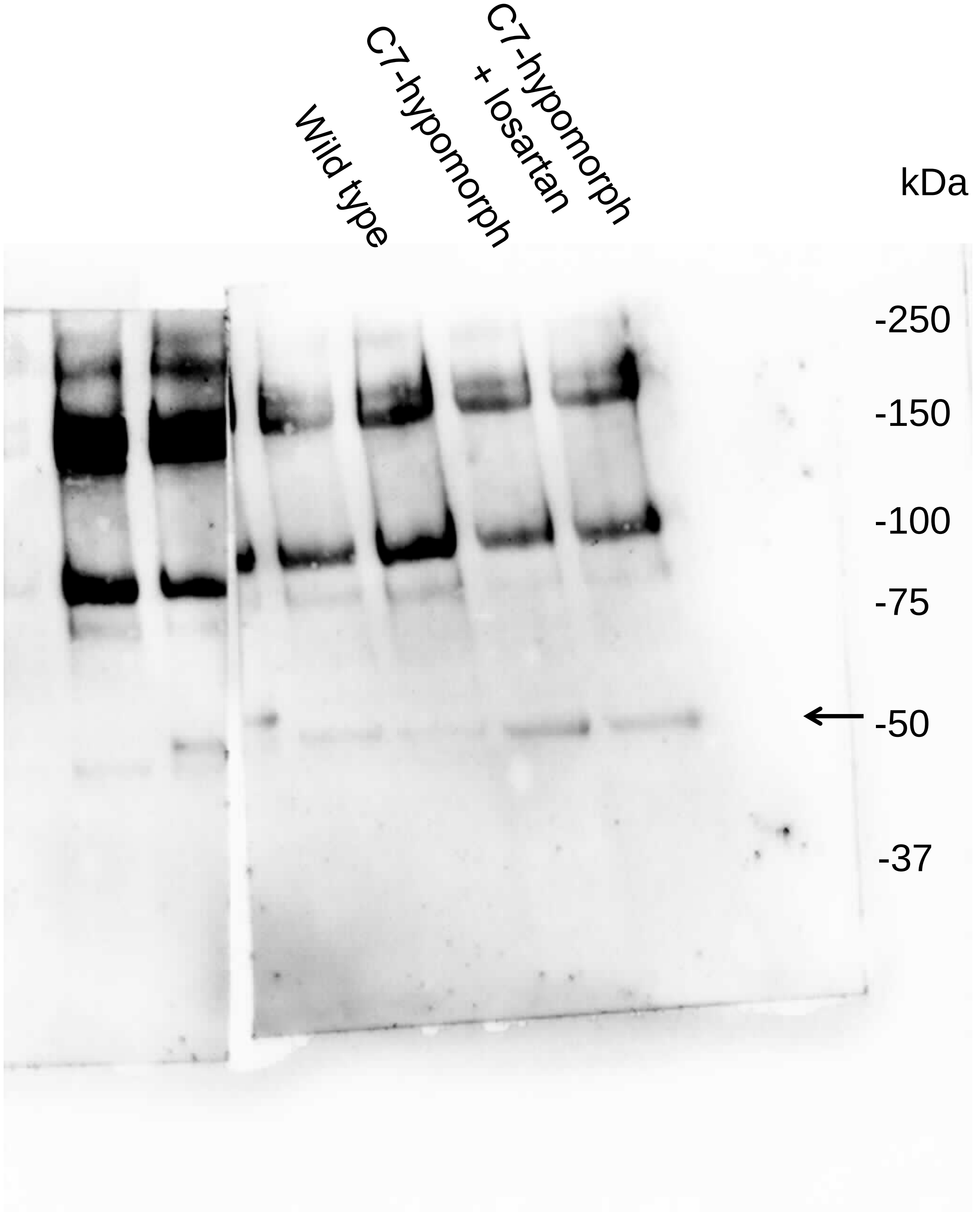

Figure 6C. Serpinf2

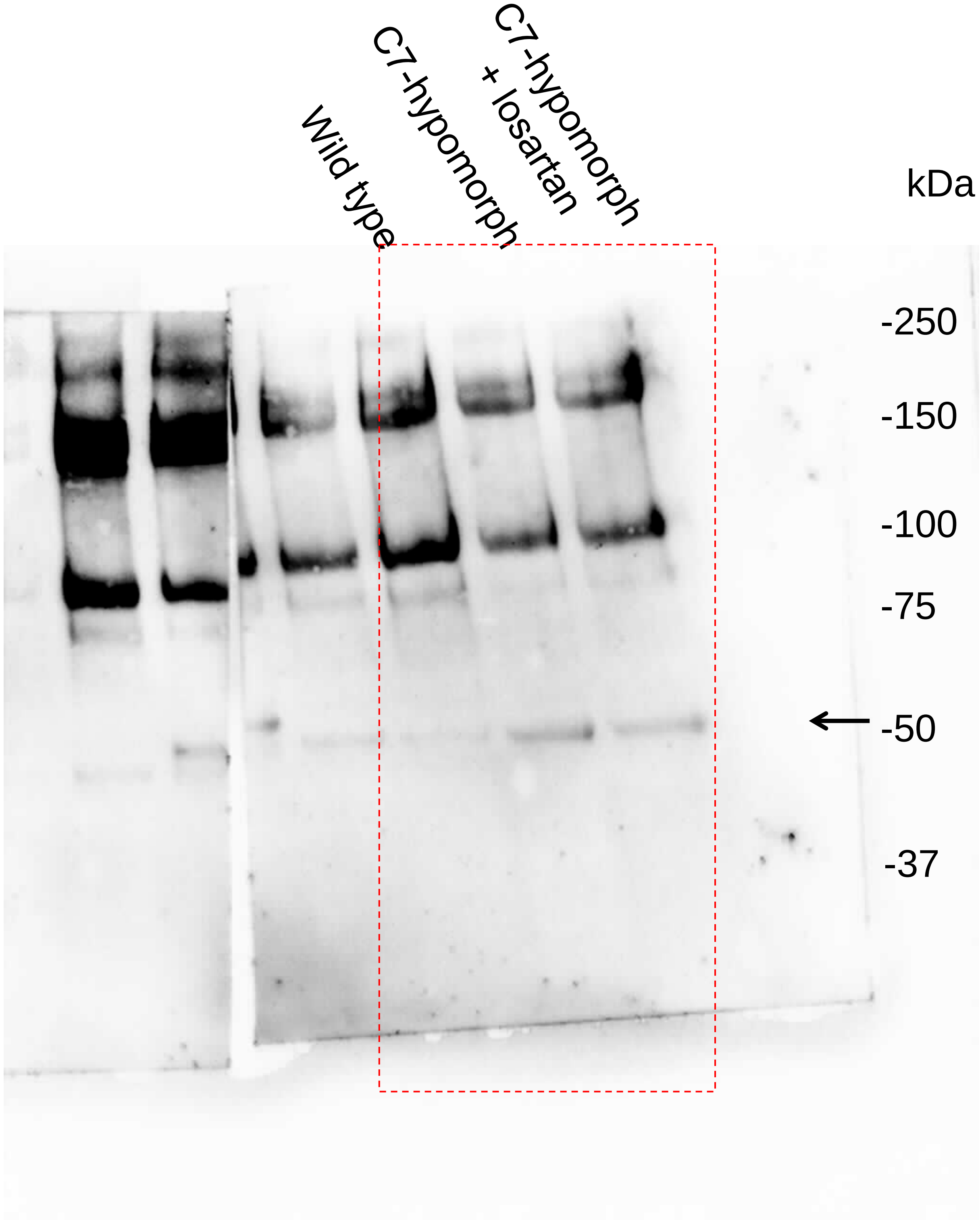

Figure 6C. Serpinf2

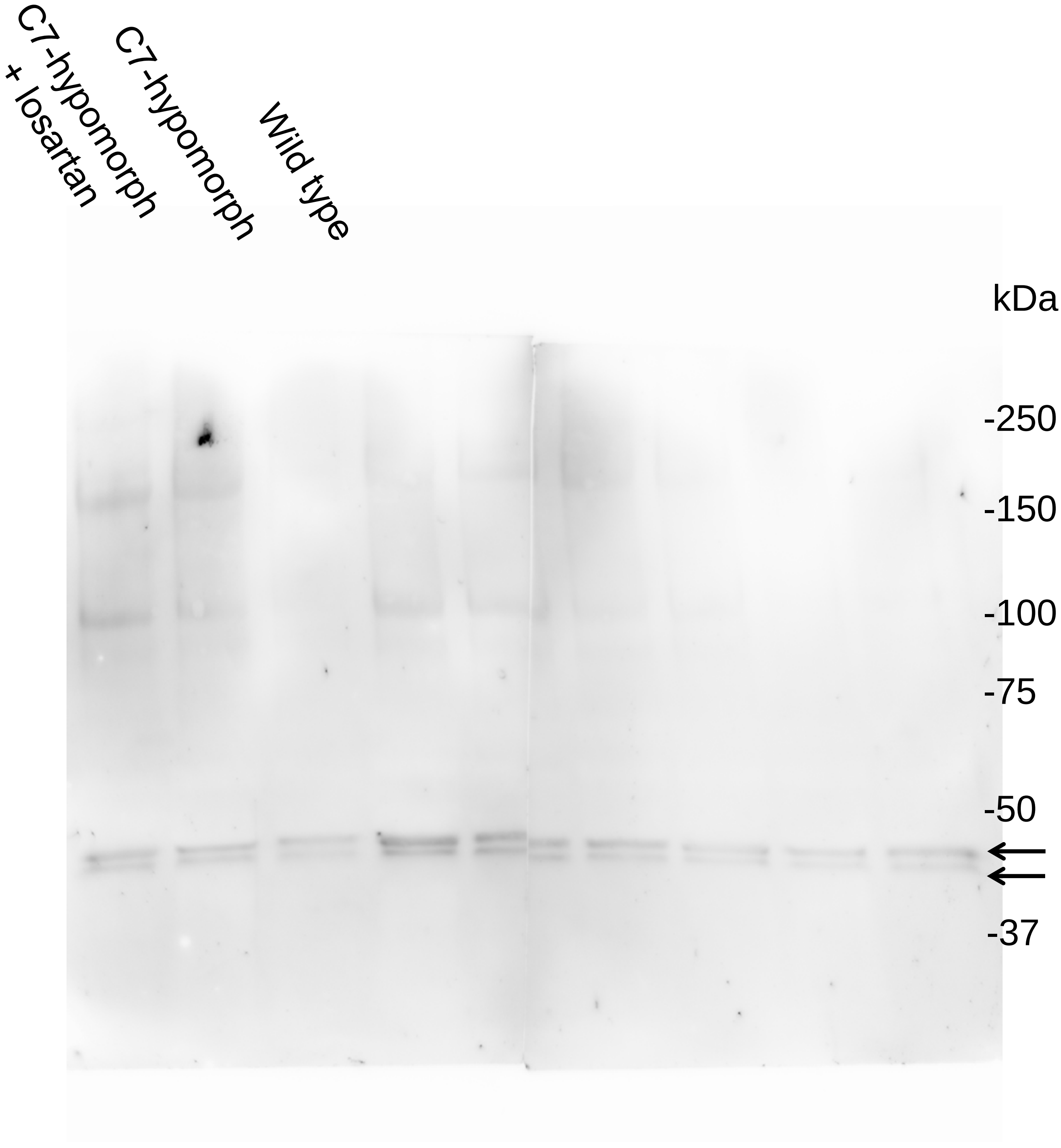

Figure 6C. Erk1/2

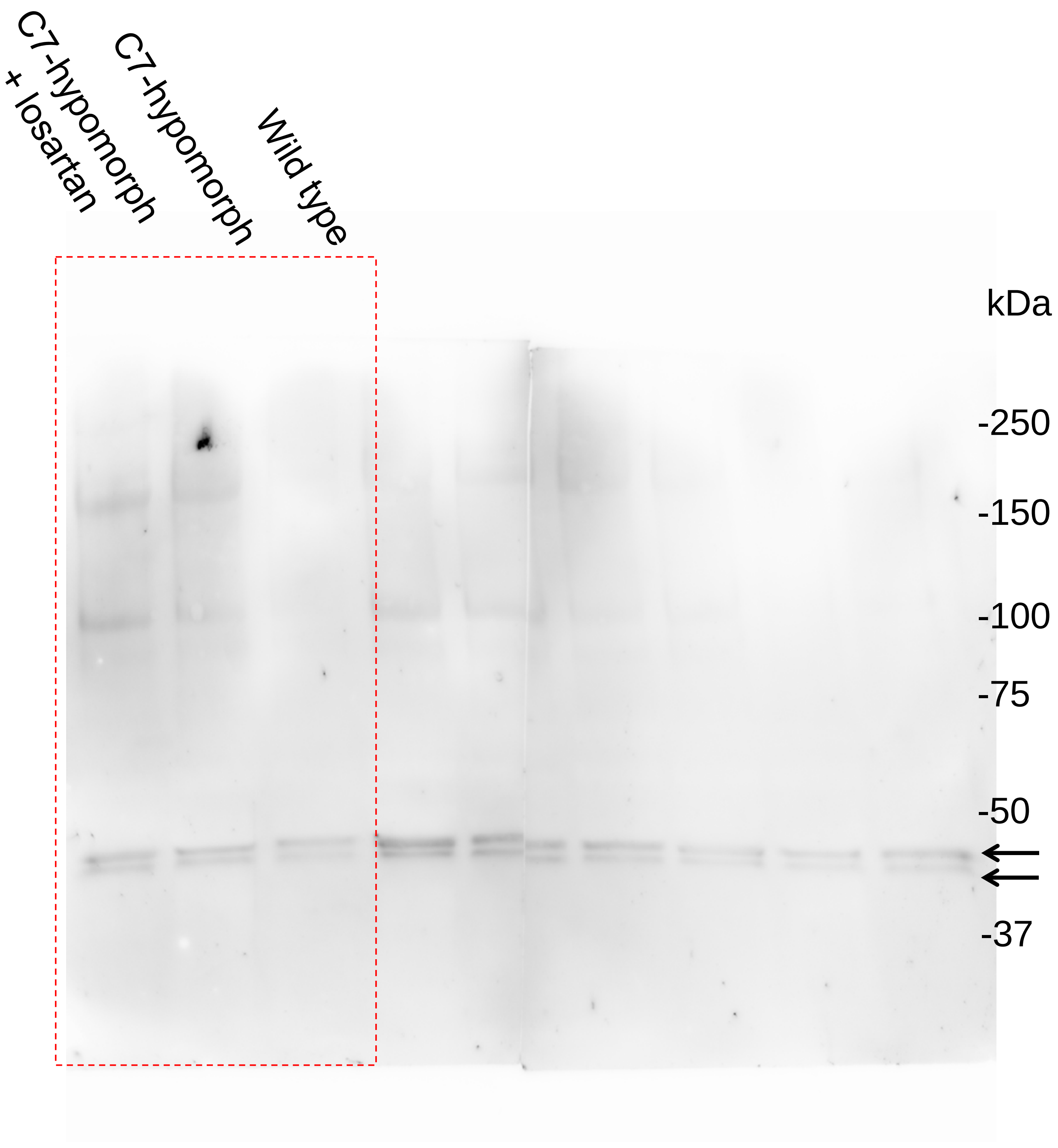

Figure 6C. Erk1/2

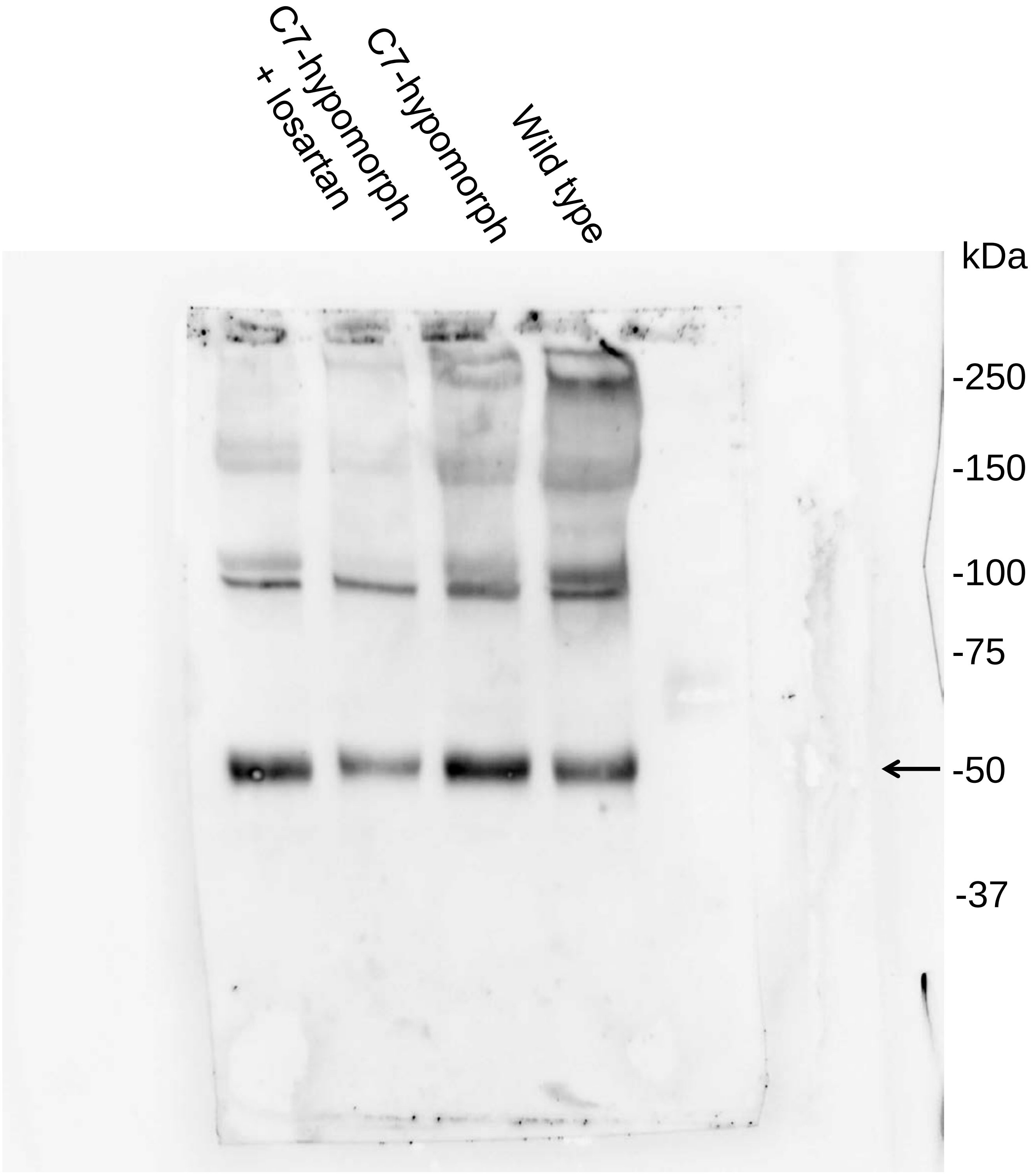

Figure 6C. Vitronectin

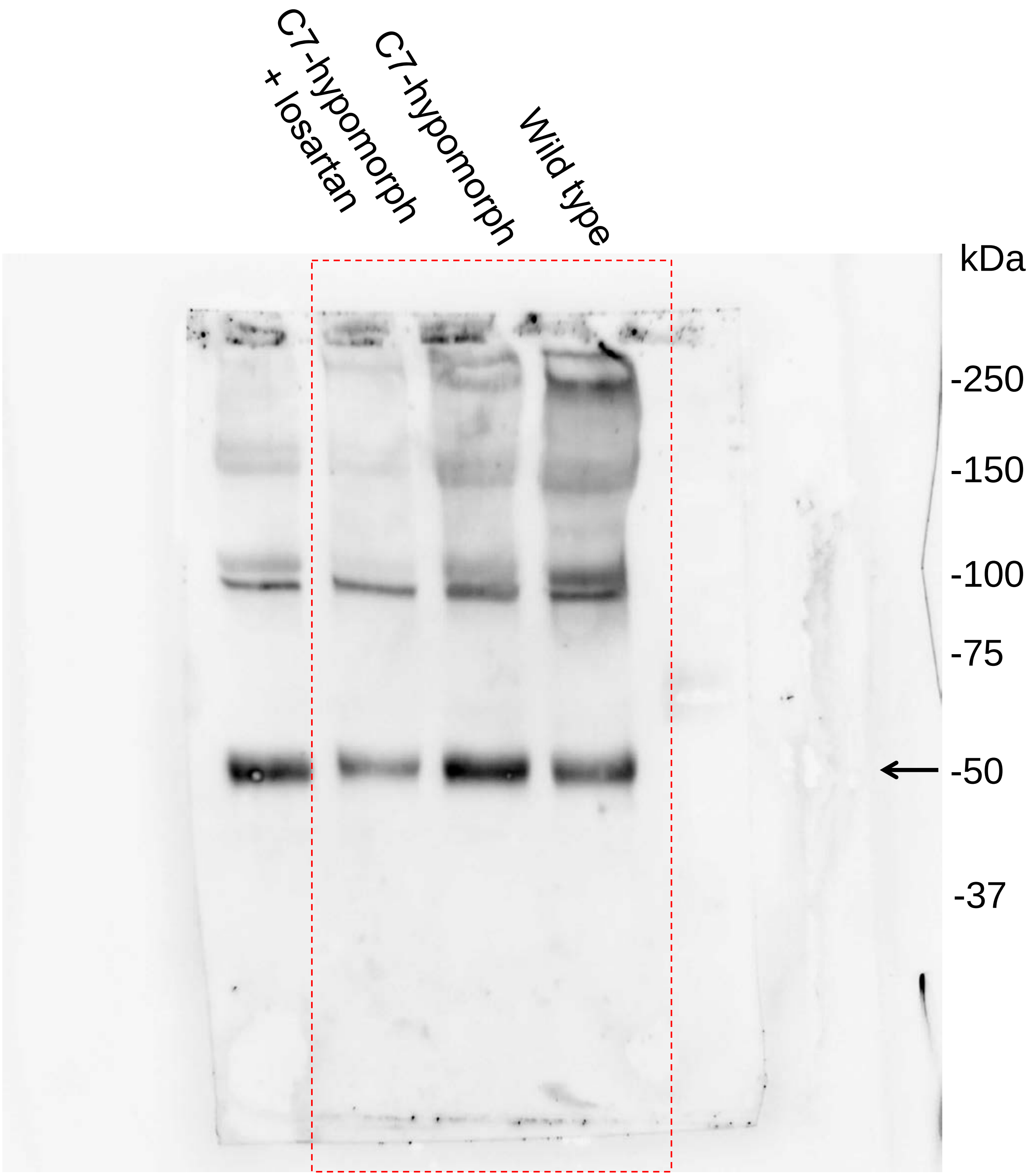

Figure 6C. Vitronectin

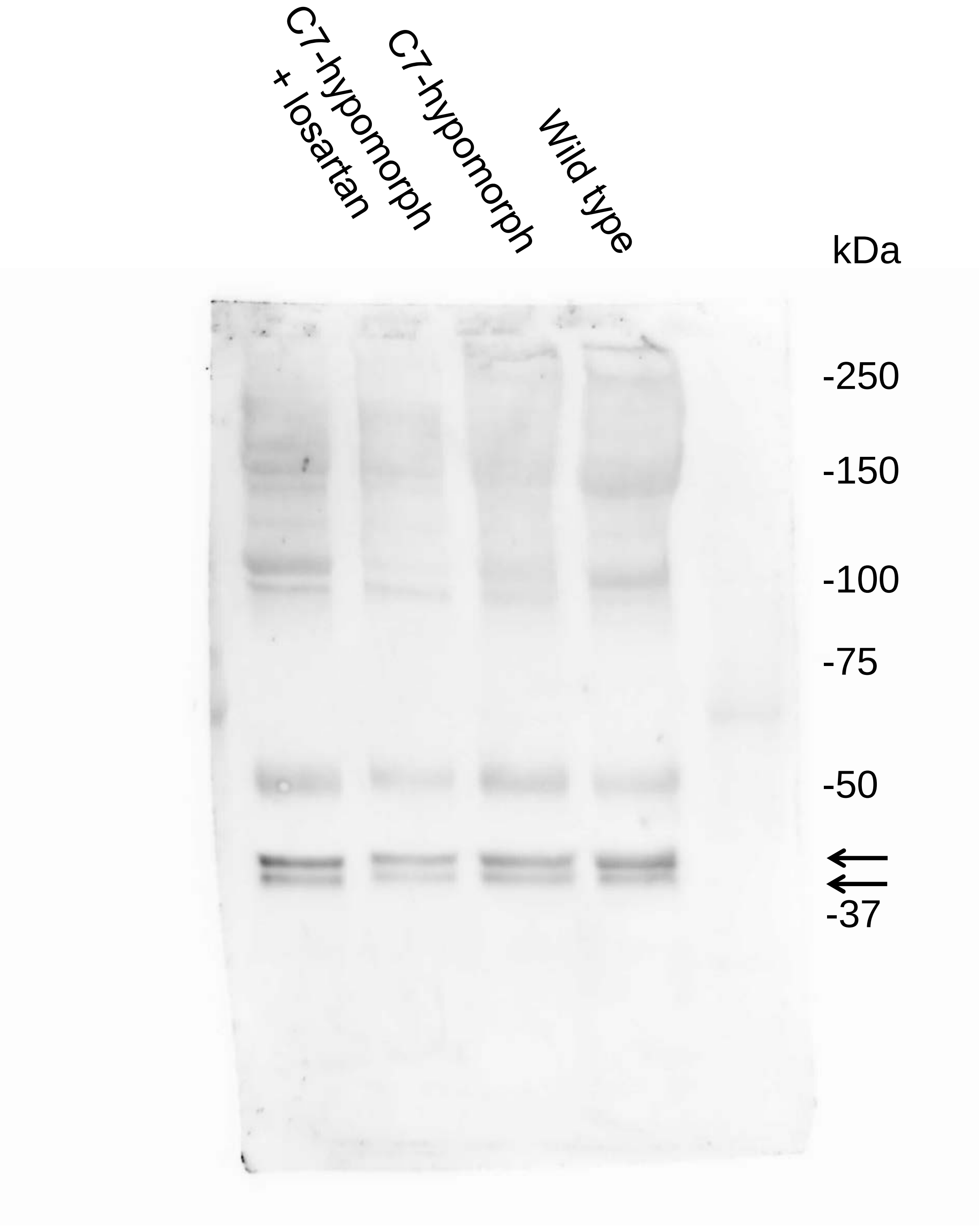

Figure 6C. Erk1/2

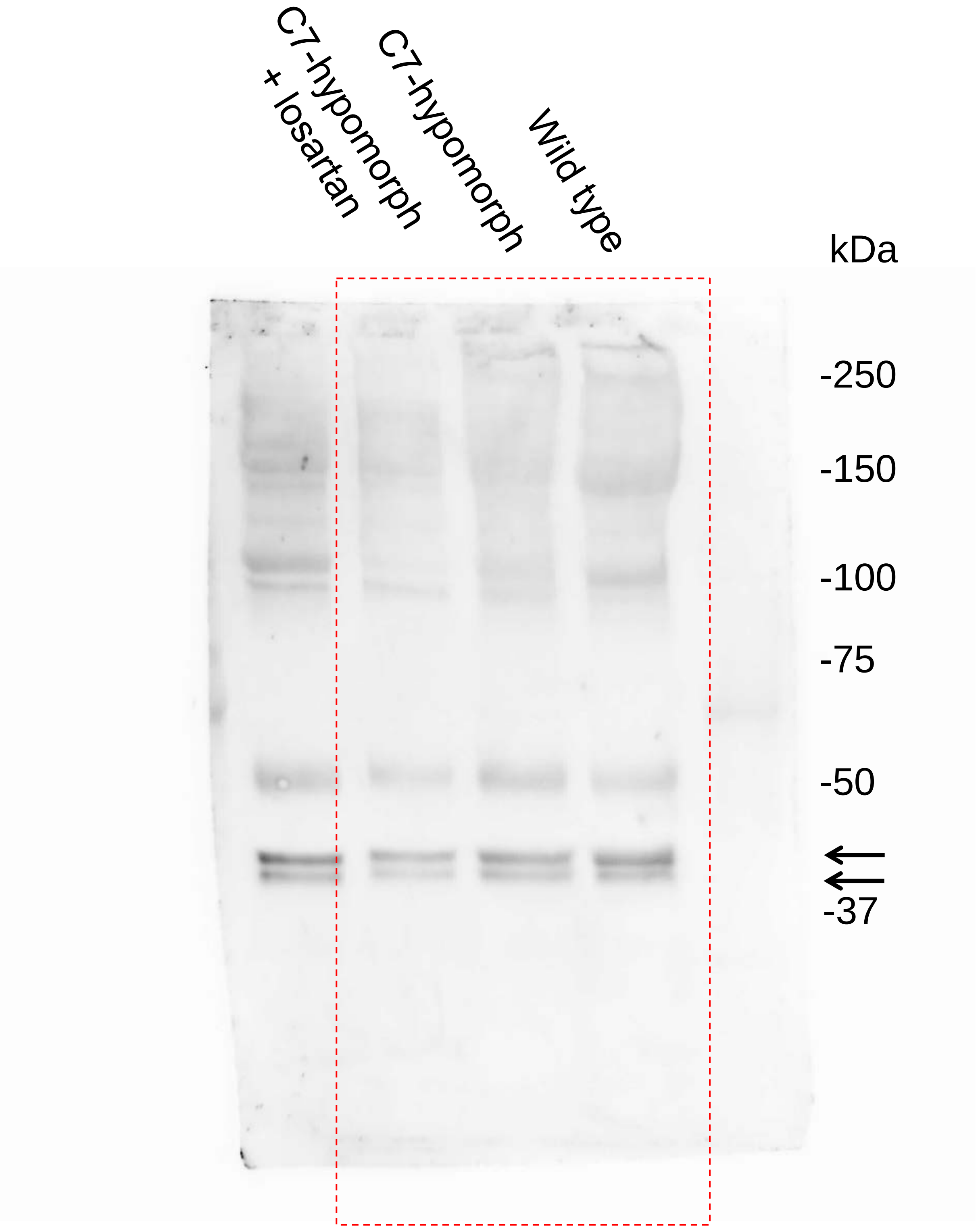

Figure 6C. Erk1/2
