# Supplementary material for: Losartan ameliorates dystrophic epidermolysis bullosa and uncovers new disease mechanisms
Source: EMBO Mol Med. 2015 Jul 20;7(9):1211–28. doi: 10.15252/emmm.201505061 (PMC4568953; doi:10.15252/emmm.201505061)
Supplement: Supplementary file 7 [file emmm0007-1211-sd7.pdf]

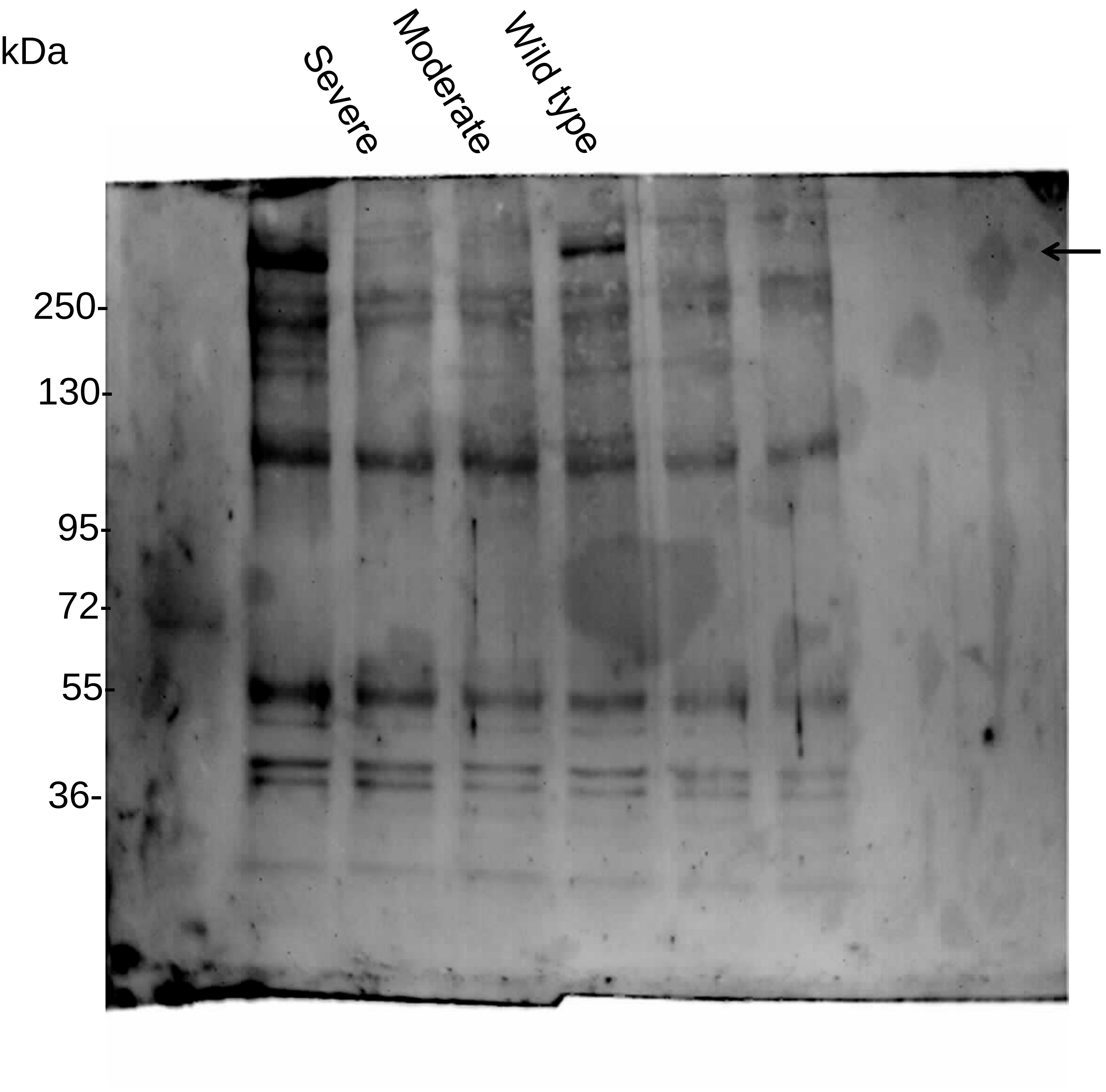

Figure 7E. C7

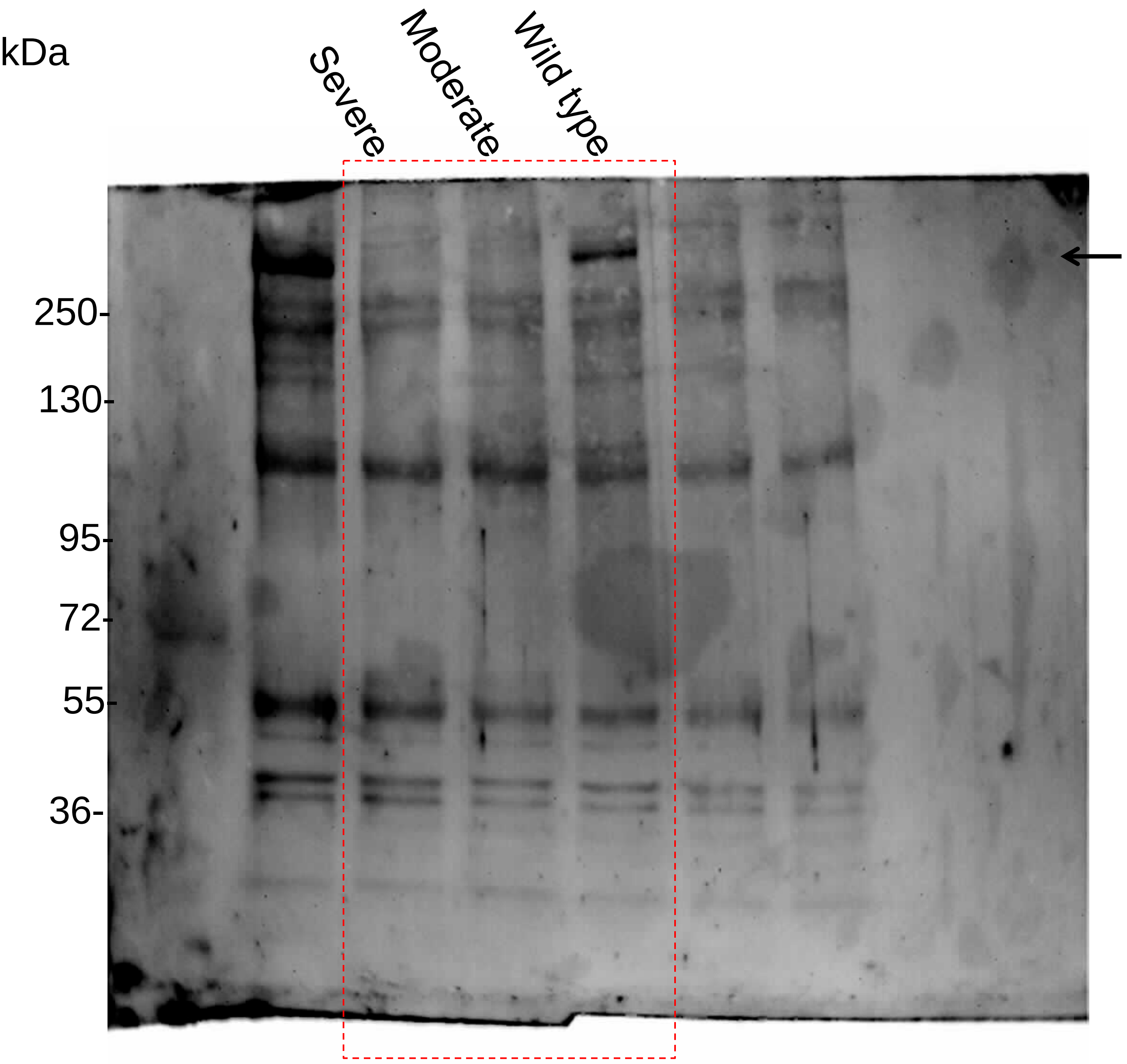

Figure 7E. C7

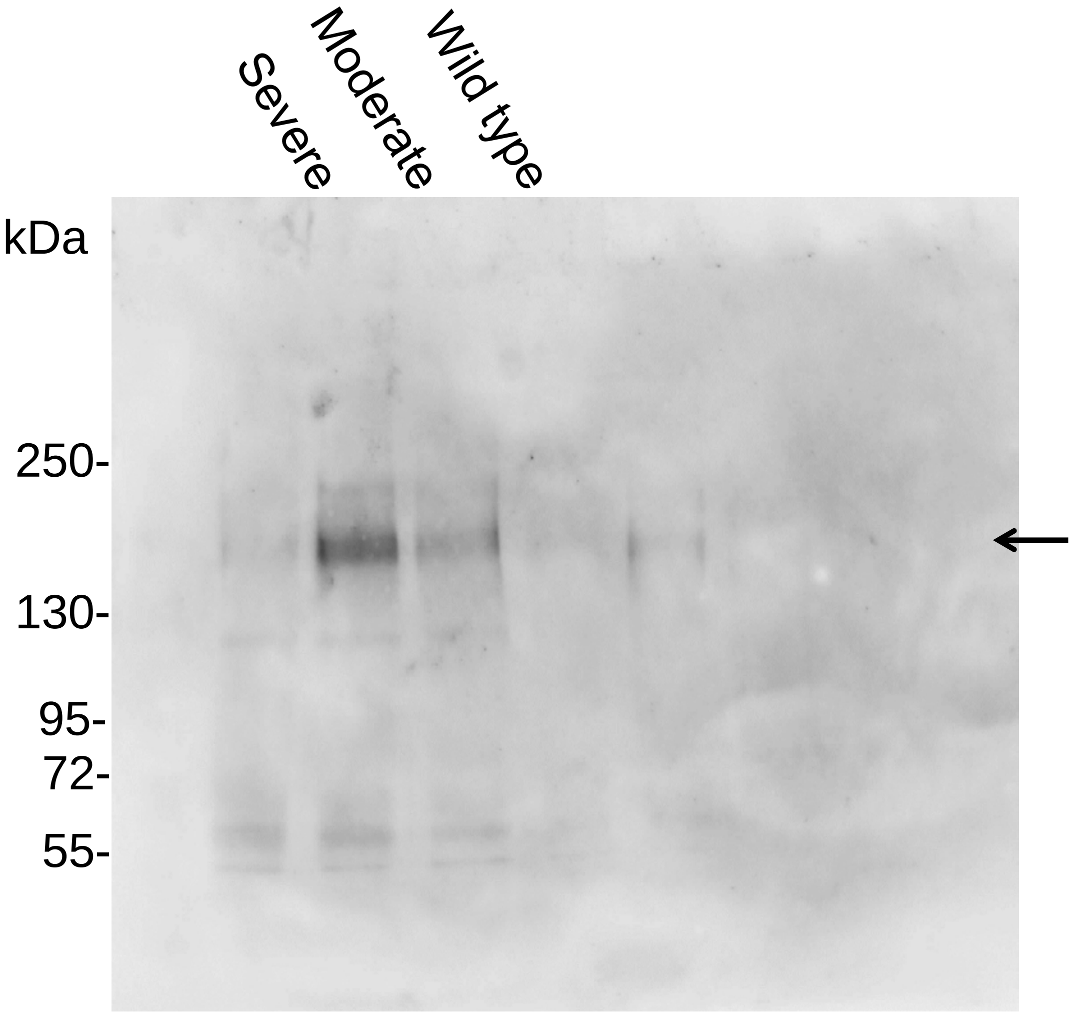

Figure 7E. Tenascin-C

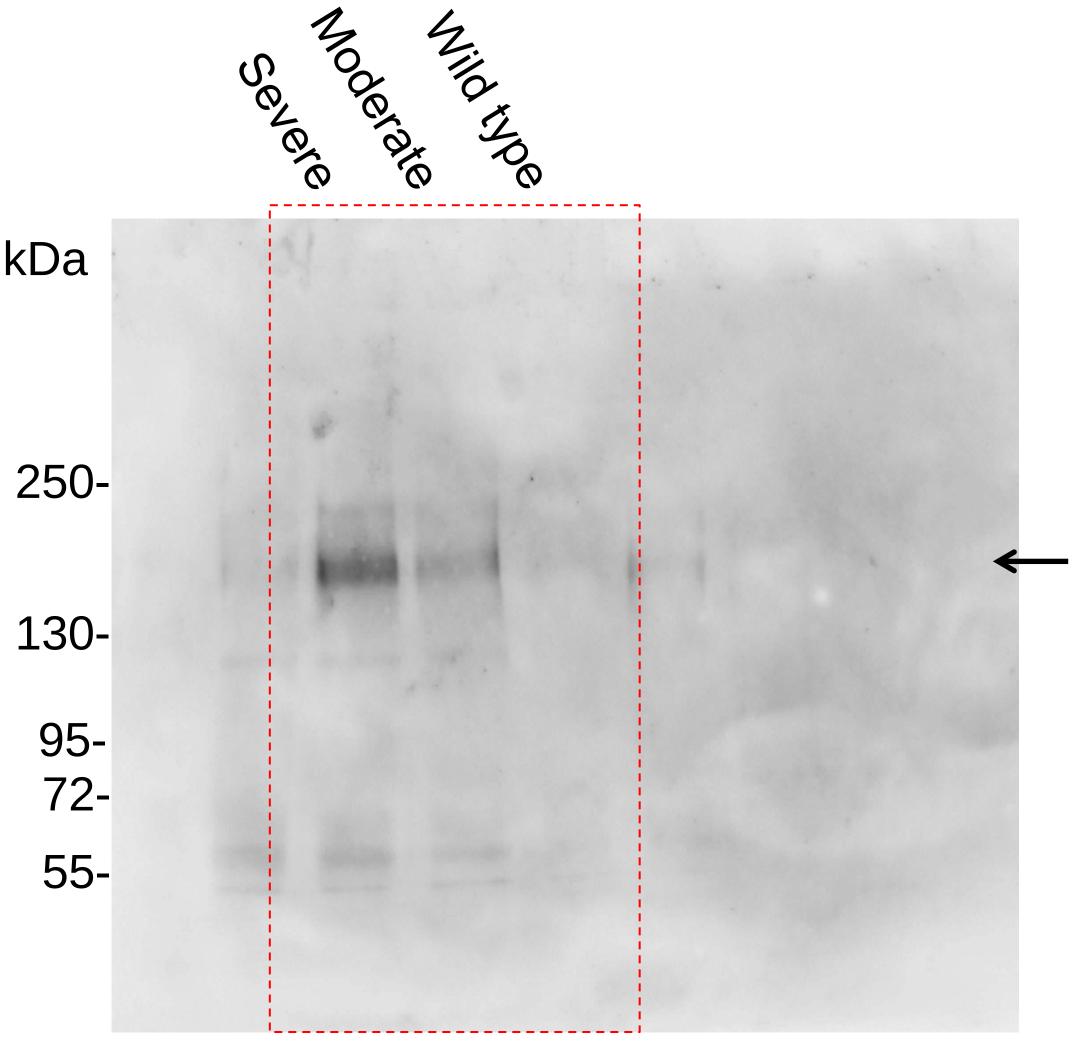

Figure 7E. Tenascin-C

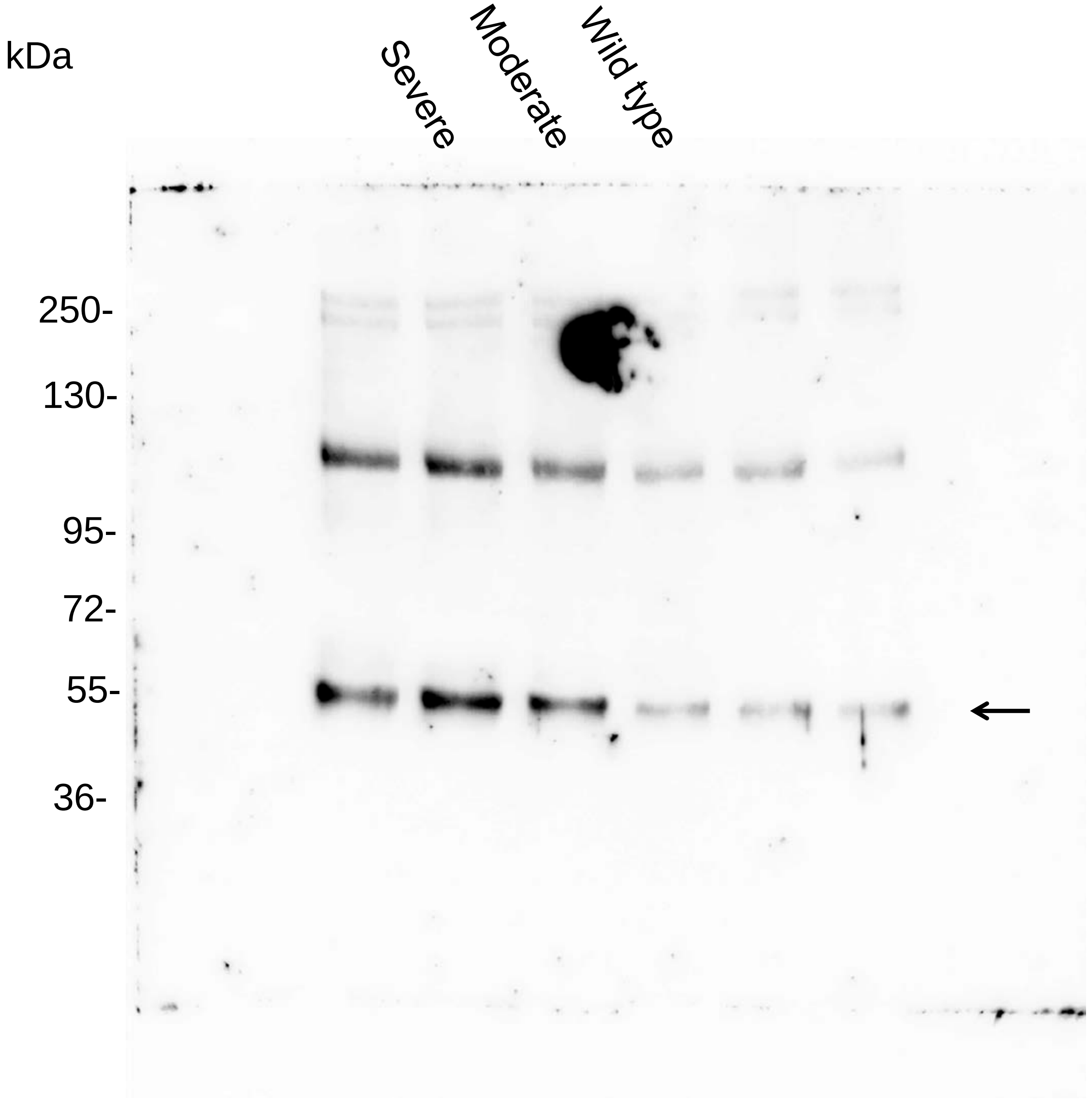

Figure 7E. C1q dimer

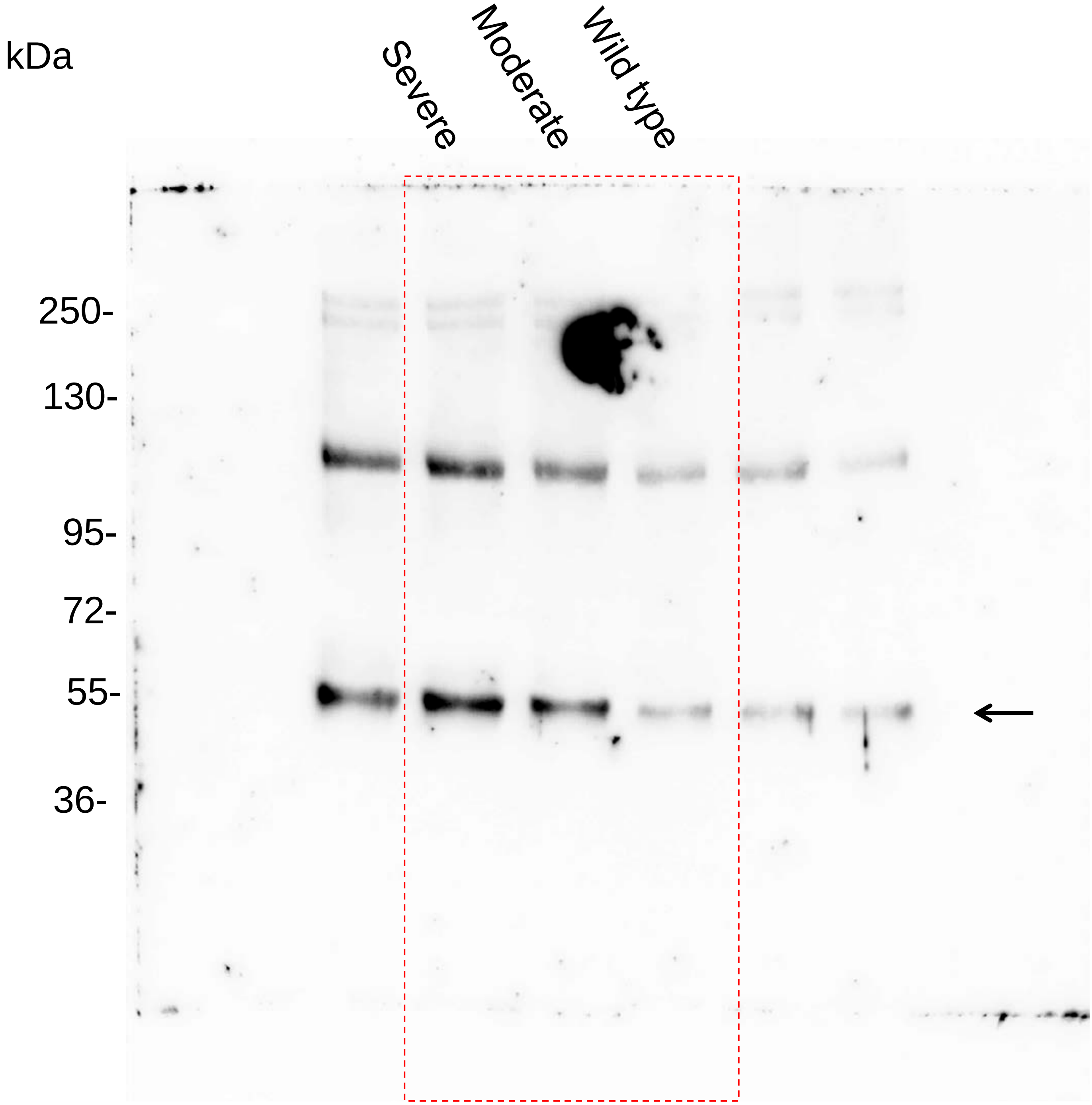

Figure 7E. C1q dimer

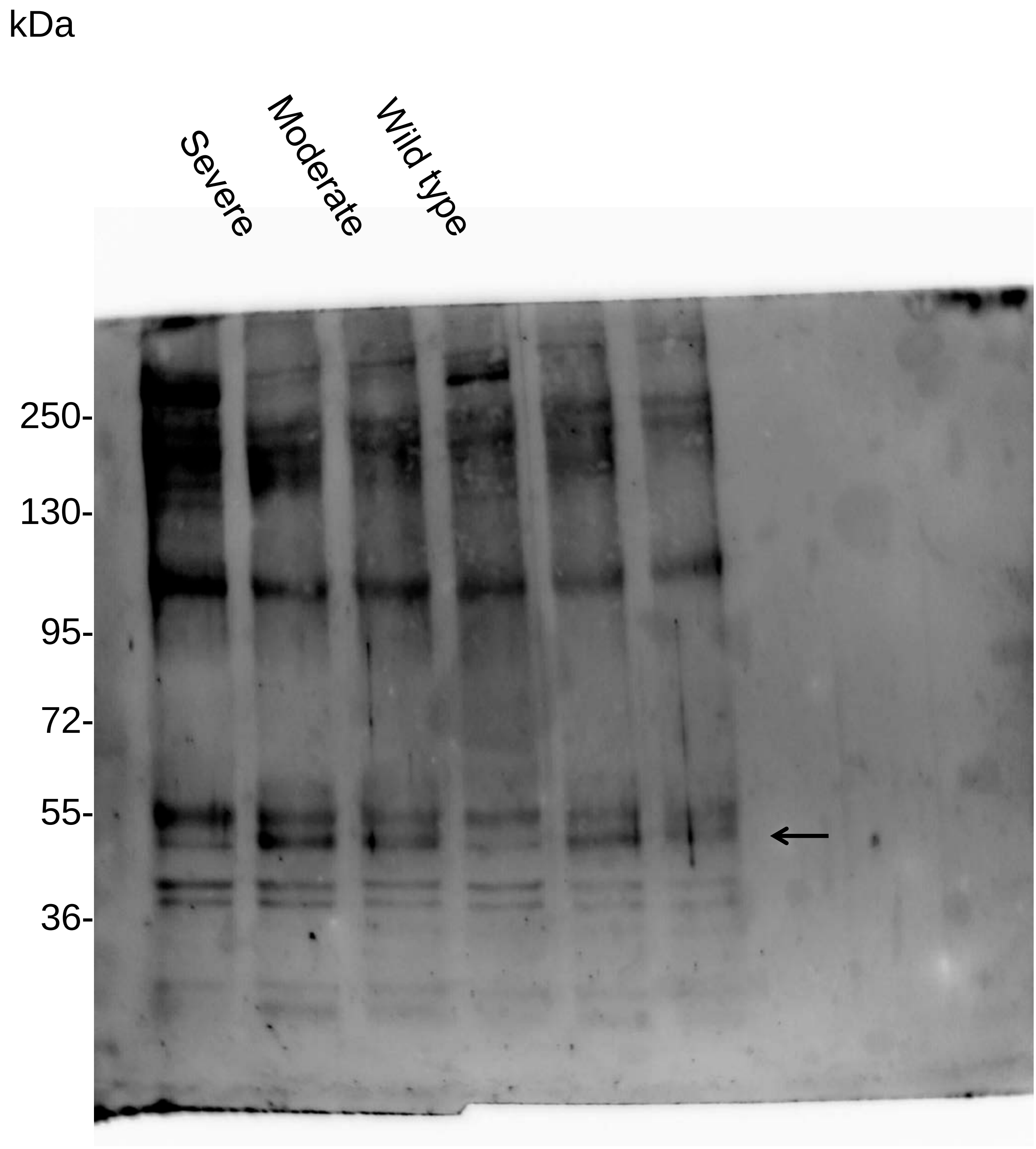

Figure 7E. IgG

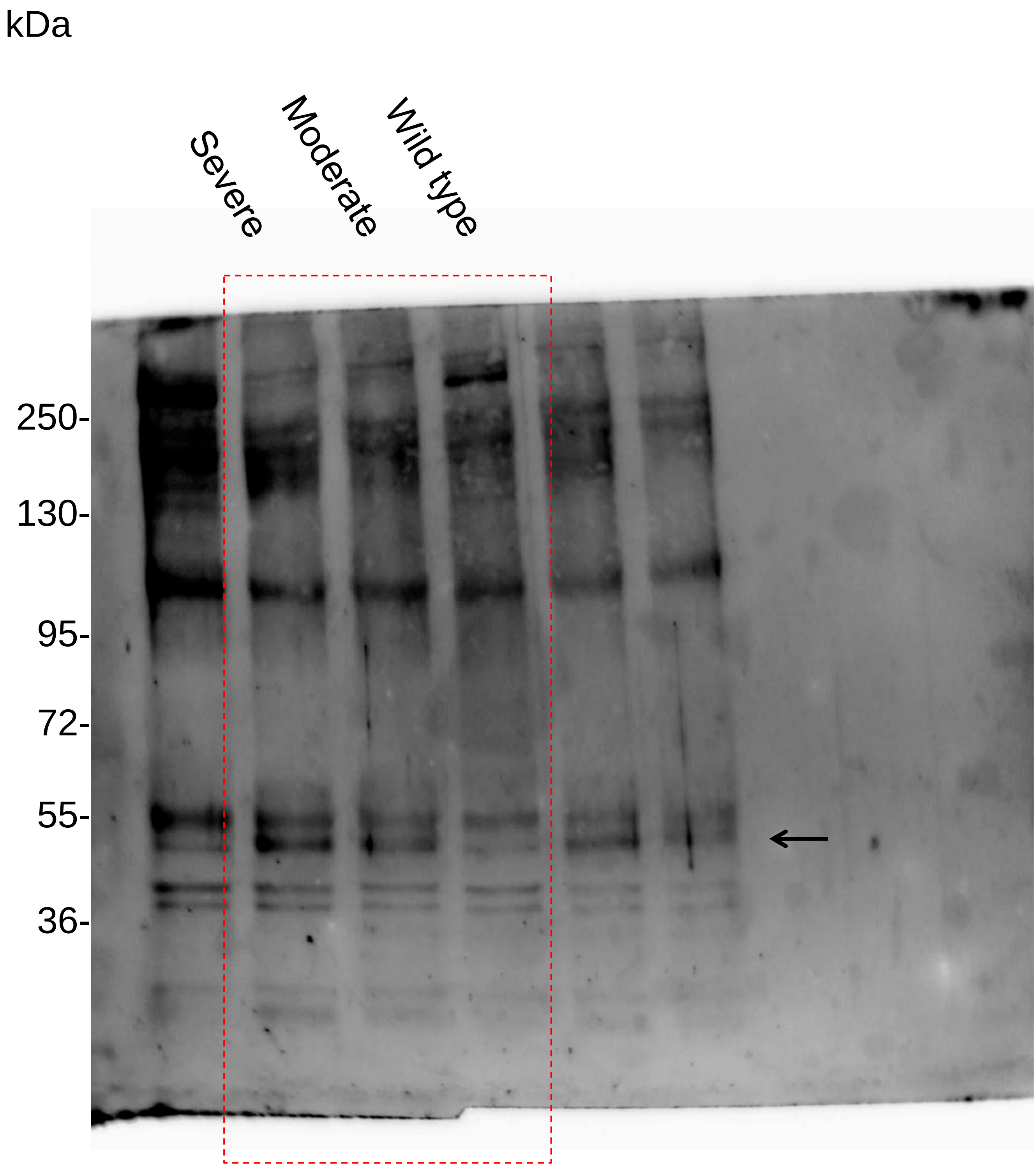

Figure 7E. IgG

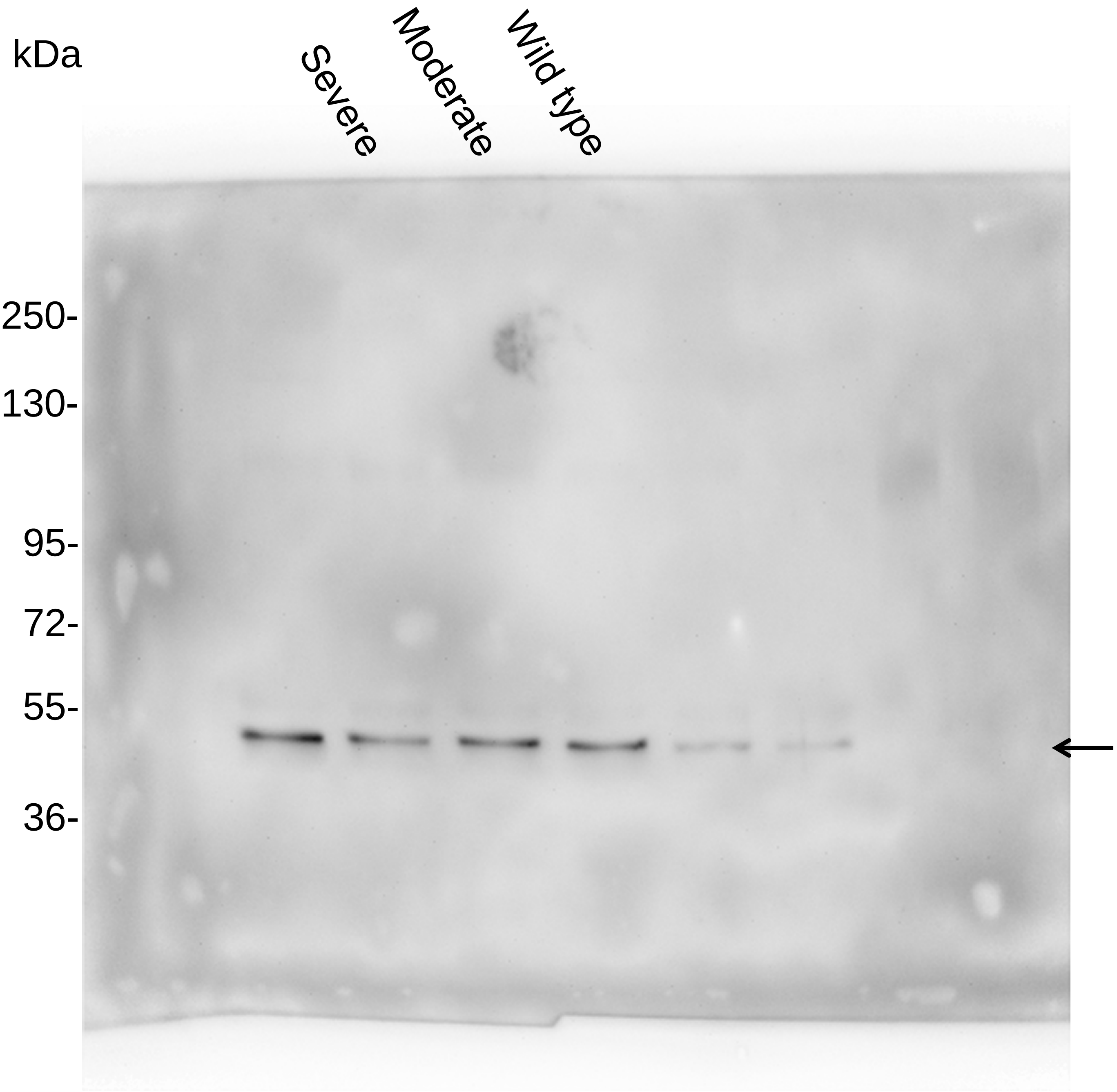

Figure 7E. β-tubulin

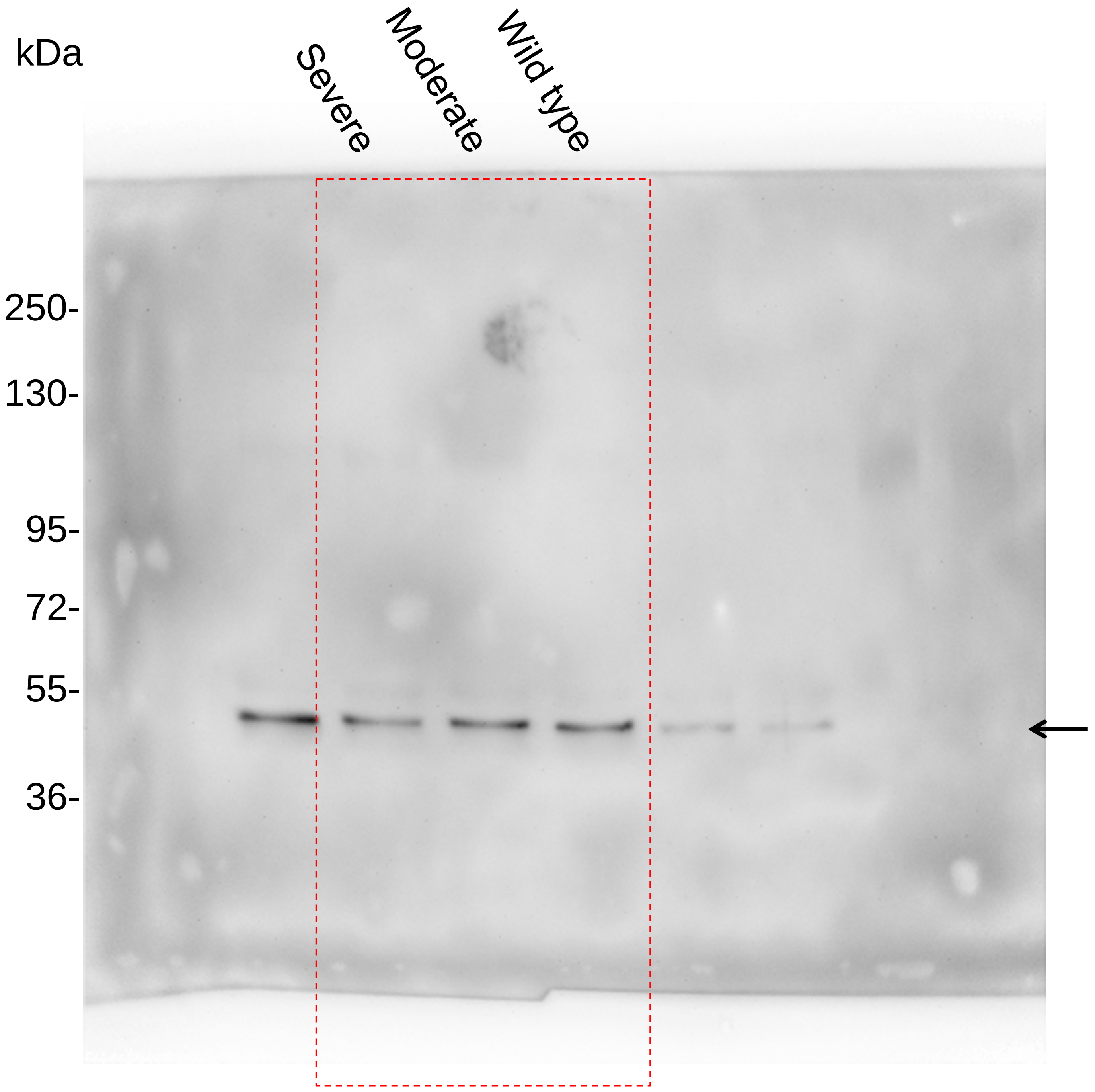

Figure 7E. β-tubulin
